# Sterol regulatory element binding protein-dependent regulation of lipid synthesis supports cell survival and tumor growth

**DOI:** 10.1186/2049-3002-1-3

**Published:** 2013-01-23

**Authors:** Beatrice Griffiths, Caroline A Lewis, Karim Bensaad, Susana Ros, Qifeng Zhang, Emma C Ferber, Sofia Konisti, Barrie Peck, Heike Miess, Philip East, Michael Wakelam, Adrian L Harris, Almut Schulze

**Affiliations:** 1 Gene Expression Analysis Laboratory, Cancer Research UK London Research Institute, 44 Lincoln's Inn Fields, London, WC2A 3LY, UK; 2 CRUK Growth Factor Group, The Weatherall Institute of Molecular Medicine, University of Oxford, John Radcliffe Hospital, Headington, Oxford, OX3 9DS, UK; 3 The Babraham Institute, Babraham Research Campus, Cambridge, CB22 3AT, UK; 4 Bioinformatics and Biostatistics Service, Cancer Research UK London Research Institute, 44 Lincoln's Inn Fields, London, WC2A 3LY, UK; 5 Present address: Koch Institute for Cancer Research, Massachusetts Institute of Technology, Cambridge, Massachusetts, 02139, USA; 6 Present address: Kennedy Institute of Rheumatology, Imperial College, 65 Aspenlea Road, London, Hammersmith, W6 8LH, UK

**Keywords:** SREBP, ER-stress, Akt, Fatty acids, Cancer

## Abstract

**Background:**

Regulation of lipid metabolism via activation of sterol regulatory element binding proteins (SREBPs) has emerged as an important function of the Akt/mTORC1 signaling axis. Although the contribution of dysregulated Akt/mTORC1 signaling to cancer has been investigated extensively and altered lipid metabolism is observed in many tumors, the exact role of SREBPs in the control of biosynthetic processes required for Akt-dependent cell growth and their contribution to tumorigenesis remains unclear.

**Results:**

We first investigated the effects of loss of SREBP function in non-transformed cells. Combined ablation of SREBP1 and SREBP2 by siRNA-mediated gene silencing or chemical inhibition of SREBP activation induced endoplasmic reticulum (ER)-stress and engaged the unfolded protein response (UPR) pathway, specifically under lipoprotein-deplete conditions in human retinal pigment epithelial cells. Induction of ER-stress led to inhibition of protein synthesis through increased phosphorylation of eIF2α. This demonstrates for the first time the importance of SREBP in the coordination of lipid and protein biosynthesis, two processes that are essential for cell growth and proliferation. SREBP ablation caused major changes in lipid composition characterized by a loss of mono- and poly-unsaturated lipids and induced accumulation of reactive oxygen species (ROS) and apoptosis. Alterations in lipid composition and increased ROS levels, rather than overall changes to lipid synthesis rate, were required for ER-stress induction.

Next, we analyzed the effect of SREBP ablation in a panel of cancer cell lines. Importantly, induction of apoptosis following SREBP depletion was restricted to lipoprotein-deplete conditions. U87 glioblastoma cells were highly susceptible to silencing of either SREBP isoform, and apoptosis induced by SREBP1 depletion in these cells was rescued by antioxidants or by restoring the levels of mono-unsaturated fatty acids. Moreover, silencing of SREBP1 induced ER-stress in U87 cells in lipoprotein-deplete conditions and prevented tumor growth in a xenograft model.

**Conclusions:**

Taken together, these results demonstrate that regulation of lipid composition by SREBP is essential to maintain the balance between protein and lipid biosynthesis downstream of Akt and to prevent resultant ER-stress and cell death. Regulation of lipid metabolism by the Akt/mTORC1 signaling axis is required for the growth and survival of cancer cells.

## Background

Cell growth requires the coordinated synthesis of macromolecules including proteins and lipids. Induction of protein synthesis is regulated by the activity of the mammalian target of rapamycin complex 1 (mTORC1), a kinase complex activated in response to growth factor signaling [1]. mTORC1 phosphorylates the ribosomal protein S6 kinases 1 and 2 (S6K1 and S6K2) and eukaryotic translation initiation factor 4E binding protein 1 (4E-BP1). The role of mTORC1 in the regulation of lipid synthesis has emerged recently [2,3]. It has been shown that mTORC1 regulates the activity of the sterol regulatory element binding proteins (SREBPs), a small family of lipogenic transcription factors. SREBPs regulate the expression of genes required for the synthesis of fatty acids and cholesterol [4]. SREBPs are expressed as inactive precursors and reside as integral trans-membrane proteins within the ER membrane where they bind to the SREBP cleavage activating protein (SCAP). When intracellular sterol concentrations are low, SREBP/SCAP complexes translocate to the Golgi where the SREBP protein is cleaved in a two-step process. This releases the N-terminal half of the protein, which translocates to the nucleus and binds to sterol regulatory element (SRE)-sequences in the promoters of its target genes [5]. Three SREBP isoforms, SREBP1a, SREBP1c and SREBP2, have been identified in mammalian cells [6].

Several lines of evidence indicate the involvement of the Akt/mTORC1 signaling axis in the regulation of SREBP. We have shown that mTORC1 is required for the nuclear accumulation of mature SREBP1 in response to Akt activation [7]. Crucially, depletion of all SREBP isoforms in immortalized human epithelial cells blocked the Akt-dependent increase in cell size, indicating that lipid synthesis is required for cell growth. Furthermore, silencing of the gene coding for SREBP in flies (HLH160/dSREBP) caused a reduction in cell and organ size [7], strongly suggesting a role for SREBP in the regulation of cell growth. mTORC1 is also required for the stimulation of lipogenesis in the liver by regulating expression of the SREBP1c gene [8], and SREBP dependent gene expression was identified as part of a metabolic regulatory network downstream of mTORC1 in cells deficient for the tuberous sclerosis complex 1 or 2 genes (TSC1 or TSC2) [9]. Interestingly, activation of SREBP1 and enhanced expression of lipogenic genes have been observed in human glioblastoma multiforme (GBM) carrying activating mutations in the epidermal growth factor receptor (EGFR) and inhibition of lipid synthesis blocked xenograft growth of glioblastoma cells expressing mutant EGFR [10]. It seems likely that cancer cells require SREBP to fulfill the increased lipid demand for rapid proliferation. However, it has not yet been investigated whether inhibition of SREBP function could affect other biosynthetic processes required for cell growth.

The unfolded protein response (UPR) is a stress pathway that is activated in response to the accumulation of misfolded proteins in the ER (also referred to as ER-stress). UPR engagement inhibits general protein translation and triggers the expression of genes required to resolve the folding defect, including ER-resident chaperones and proteases. Prolonged ER-stress or failure to repair the damage leads to the induction of apoptosis. The ER-stress response consists of three main pathways with partially overlapping functions [11]. Accumulation of unfolded proteins in the ER induces activation of the inositol-requiring protein-1 (IRE1), an ER-resident endonuclease [12]. IRE1-mediated splicing of X-box binding protein 1 (*XBP-1*) mRNA allows translation of this transcription factor and leads to expression of genes involved in degradation of misfolded proteins within the ER lumen [13]. Interestingly, XBP-1 also regulates the expression of genes involved in the synthesis of membrane phospholipids, thereby connecting ER-stress to membrane biogenesis [14]. The second arm of the ER-stress response involves the proteolytic activation of the activating transcription factor-6 (ATF6) [15] and controls the expression of chaperones and other factors involved in protein quality control [16]. ER-stress also activates the eukaryotic translation initiation factor 2-alpha kinase 3 (EIF2AK3 also known as PERK), which phosphorylates the α-subunit of the eukaryotic translation initiation factor-2 (eIF2α) on serine 51. This inhibits the guanine nucleotide exchange factor eIF2B, thereby preventing general protein synthesis [17] while specifically facilitating the translation of the activating transcription factor-4 (ATF4). ATF4 induces expression of the C/EBP-homologous protein (CHOP), a transcription factor that regulates the expression of pro-apoptotic genes in response to ER-stress [18]. The complete program of transcriptional and translational changes triggered by eIF2α phosphorylation is known as the integrated stress response (ISR). It induces the expression of genes involved in amino acid metabolism and resistance to oxidative stress and supports the cellular adaptation to conditions of ER-stress [19].

Chemical inhibition of cholesterol biosynthesis has been shown to induce the ISR, while activation of PERK reduced the accumulation of mature SREBP in response to sterol depletion [20]. Another study found that PERK regulates lipogenesis during mouse mammary gland development by inhibiting the translation of the insulin-induced gene 1 (INSIG1), an inhibitor of SREBP processing [21]. Furthermore, activation of eIF2α phosphorylation by the eukaryotic translation initiation factor 2 alpha kinase 4 (GCN2) induced expression of the SREBP1c gene through an unknown mechanism [22].

Since the production of biomass during cell growth requires the synchronized regulation of different biosynthetic processes, we speculated that protein and lipid biosynthesis downstream of the Akt/mTORC1 pathway might be intricately linked. We found that inhibition of SREBP function induced ER-stress when the supply of exogenous lipids was reduced. SREBP inhibition blocked Akt-dependent protein synthesis and caused alterations in cellular lipid composition characterized by a marked reduction in unsaturated fatty acids. Importantly, induction of ER-stress was exacerbated by activation of the Akt/mTORC1 pathway, while the addition of exogenous oleate prevented the induction of the ER-stress response. Inhibition of SREBP also caused increased levels of reactive oxygen species (ROS), and induction of ER-stress could be blocked by anti-oxidant treatment. Silencing of SREBP1 was sufficient to induce ER-stress and apoptosis in U87 human glioblastoma cells under lipoprotein-deplete conditions. Importantly, depletion of SREBP1 also inhibited tumor growth in a xenograft model. These findings indicate that SREBP-dependent lipid synthesis and desaturation are essential to prevent the engagement of the ER-stress response pathway and to allow cell growth and tumor formation.

## Methods

### Cell culture and reagents

RPE myrAkt-ER cells and culture conditions have been described before [23]. U87-GFP cells were grown in DMEM supplemented with 10% FCS and 4 mM glutamine. Breast cancer cell lines were obtained from CRUK LRI Cell Services (London, UK) and grown in DMEM/F12 supplemented with 10% FCS and 2 mM glutamine. Lipoprotein deficient serum was obtained from Intracel (Frederick, MD, USA). Lipid depleted serum was generated using Liposorb™ resin from Calbiochem (Darmstadt, Germany) according to manufacturer’s instructions. The following antibodies were used: SREBP1 (2A4), SREBP2 (1C6) (BD Biosciences (Franklin Lakes, NJ, USA), PERK, eIF2α, phospho-eIF2α, PARP (Cell Signaling Technology, Danvers, MA, USA), phospho-PERK, ATF6, ATF4 (Santa Cruz Biotechnology, Santa Cruz, CA, USA), SCD (Alpha Diagnostic International, San Antonio, TX, USA) and horseradish peroxidase conjugated beta actin (Sigma, Poole, UK). 4-hydroxytamoxifen, C75, cerulenin, compactin, 4-phenyl butyric acid, oleic acid- albumin, N-acetyl-L-cysteine and tunicamycin were from Sigma. Stearic acid (Sigma) was coupled to BSA at a 4:1 molar ratio. Thapsigargin and caspase 3/7 substrate were from Calbiochem. SCD inhibitor (A939572) was from Biovision (Milpitas, CA, USA). Doxycycline hyclate was from BD Biosciences. Fatostatin was from Early Discovery Chemistry (Hove, UK).

### Retroviral transduction

The full-length cDNA for human SCD was amplified by reverse transcriptase PCR (RT-PCR) and cloned into pBabe-blast. Retroviral particles were generated in Phoenix Eco packaging cells, and cells were selected with 10 μg/ml blasticidin (Invitrogen, Carlsbad, CA, USA).

### RNA interference

RPE cells were transfected with 50 nM siRNA oligonucleotides using DharmaFECT™ reagent 1 (Dharmacon, Lafayette, CO, USA) following a reverse transfection protocol. siRNA sequences are provided in Additional file 1 supplemental information.

### Microarray analysis

Total RNA from RPE-myrAkt-ER cells transfected with either control oligonucleotides (Dharmacon siGENOME control 3) or siRNA oligonucleotides targeting SREBP1 or SREBP2 (Dharmacon Smartpools) was used for transcriptome analysis on Illumina human Ref-8 arrays. Data represent three independent experiments. Information on data analysis is provided as Additional file 1 supplemental information.

Gene Set Enrichment Analysis (GSEA) was performed using gene sets derived from published literature. In order to avoid false positives due to multiple testing in GSEA, the false discovery rate (FDR) was used to adjust the *P*-value to give the *Q*-value. A *Q*-value of <0.05 is statistically significant.

### X-box binding protein mRNA splicing assay

*XBP-1* mRNA was amplified from 50 ng cDNA using 0.6 μM primers, 250 mM MgCl_2_, and 0.25 U of Simpler Red Taq DNA polymerase (Applied Biosystems, Foster City, CA, USA) in a final volume of 25 μL, at an annealing temperature of 66°C for 35 cycles. Forward primer: 5’-AAACAGAGTAGCAGCTCAGACGC-3’; reverse primer: 5’-TCCTTCTGGGTAGACCTCTGGGAG-3’. PCR products were digested with PstI and separated on a 3% agarose gel. A 448 base pair amplicon indicates spliced *XBP-1* (XBP-1 s).

### Protein synthesis

Protein synthesis was determined following 92 hours of gene silencing. Cells were washed twice in PBS then incubated for 4 hours in cysteine/methionine-free media containing 0.5% bovine serum albumin (BSA), glutamine and 10 μCi of ^35^S Express Protein Labelling Mix (Perkin Elmer, Waltham, MA, USA), in the presence of either ethanol or 4-OHT, then lysed in RIPA buffer. Soluble proteins were precipitated from cell lysates with 25% final concentration of trichloracetic acid (TCA) and 10 μg BSA. Precipitates were centrifuged, washed twice in 10% TCA and twice in ethanol, prior to scintillation counting. Data were normalized using total protein content determined by sulforhodamine B assay (Sigma) from parallel cultures.

### Determination of ROS levels

Cells were incubated with 3 μM CM-H_2_DCFDA for 30 minutes or with 2.5. μM MitoSOX (both Invitrogen, Carlsbad, CA, USA) for 15 minutes at 37°C, trypsinized and washed twice with PBS, stained with DAPI and analyzed on a LSRII-SORP flow cytometer (Becton Dickinson, Franklin Lakes, NJ, USA).

### Analysis of cellular respiration

Experiments were performed in a 96-well format using a Seahorse Bioscience (North Billerica, MA, USA) XF96 Extracellular Flux Analyser (Software Version 1.4) in Seahorse Bioscience assay medium supplemented with 1 mM sodium pyruvate and 10 mM Glucose and pH was adjusted to 7.4. During the experiment, 1.264 μM oligomycin A (Sigma), 0.4 μM FCCP (Sigma), and a mix of 1 μM rotenone (Sigma) and 1 μM antimycin A (Sigma) were injected. Oxygen consumption rates (OCR) were measured over time and normalized to total protein content determined by sulforhodamine B staining.

### Lipid analysis by mass spectrometry

Lipids were extracted using a methanol/chloroform extraction method and quantified by Liquid chromatography-mass spectrometry (LC-MS) analysis on a Shimadzu (Kyoto, Japan) IT-TOF LC/MS/MS system. Accurate mass (with mass accuracy approximately 5 ppm) and tandem MS were used for molecular species identification and quantification. The identity of lipids was further confirmed by reference to appropriate lipid standards. A detailed description of the procedure is provided in the Additional file 1 supplemental information.

### Cell viability assay

Caspase 3/7 activity was measured using Caspase-3 substrate IX, fluorogenic, (Calbiochem). Cells were fixed with trichloroacetic acid and normalized to total protein content determined by sulforhodamine B staining.

### Lipid synthesis

Cells were incubated in medium containing 10 μCi/ml [1-^14^C] acetate (85 μM final concentration, Perkin Elmer) for 4 hours. After washing twice in PBS cells were trypsinized and lysed in 0.5% Triton X-100/PBS. Lipids were extracted by successive addition of 2 ml methanol, 2 ml chloroform, and 1 ml dH_2_O. Phases were separated by centrifugation before the organic phase was dried and used for scintillation counting. Results were normalized to total protein content as determined by sulforhodamine B staining.

### Xenograft experiments

Male nude mice (nu/nu) aged 4 to 6 weeks were injected subcutaneously with 10^5^ U87-GFP-Tet-pLKO-SREBP1 cells into the dorsal flank. After 8 days, animals were subdivided into two experimental groups, a doxycycline treated group and a non-treated group. For induction of shRNA expression, mice were treated with 0.2 g/kg doxycycline in food pellet (Doxycycline diet, D.98186, Harlan Laboratories, Wyton, UK) and tumor growth was followed over 30 days. Tumor volume was determined using the ellipsoidal volume formula: 1/2 x length x width^2^. All animal experiments were performed according to UK Home Office guidelines (license number PPL 80/2330) and have been approved by a local ethics committee.

Additional methods are provided in the Additional file 1 supplemental information.

## Results

### Combined depletion of SREBP1 and SREBP2 induces expression of genes involved in the endoplasmic reticulum-stress response

**Figure 1:**
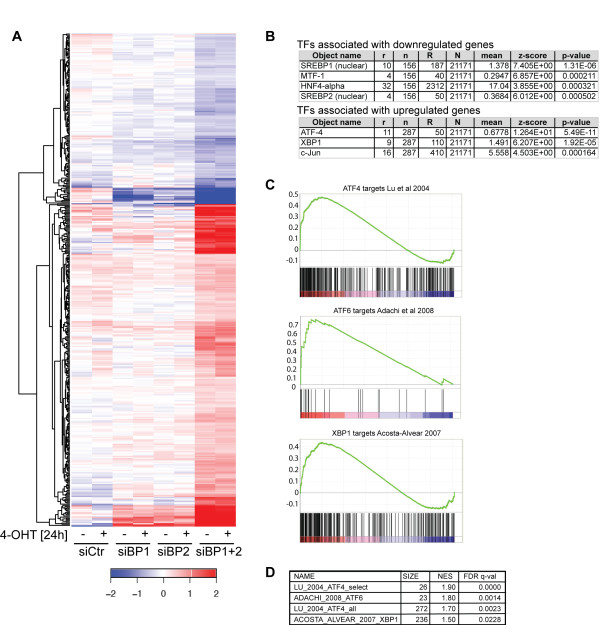
**Combined ablation of SREBP1 and SREBP2 induces a transcriptional program indicative of endoplasmic reticulum**-**stress activation. **RNA from cells after silencing of control (siCtr), SREBP1 (siBP1), SREBP2 (siBP2) or both (siBP1 + 2) treated with 100 nM 4-OHT or solvent (ethanol) for 24 hours in medium containing 1% lipoprotein deficient serum (LPDS) was used for microarray analysis. Genes regulated in response to combined silencing of SREBP1 and SREBP2 were identified using a false discovery rate (FDR) of 0.01. (**A**) Heat map showing a two-way cluster analysis of the 417 genes regulated in response to silencing of SREBP1 and SREBP2. (**B**) Transcription factors (TFs) associated with genes regulated in response to SREBP1 and SREBP2 silencing. r: number of targets in the dataset regulated by this TF; n: number of network objects in the dataset; R: number of targets in the database regulated by this TF; N: total number of gene-based objects in the database; mean: mean value for hypergeometric distribution (n*R/N); z-score: z-score ((r-mean)/sqrt(variance)); *P*-value: probability to have the given value of r or higher (or lower for negative z-scores). (**C**) Gene set enrichment analysis (GSEA) was used to study association with transcriptional response to endoplasmic reticulum (ER)-stress. Enrichment plot of gene sets of ATF4, XBP-1 and ATF6 target genes from the literature. (**D**) Enrichment scores for gene sets derived from the literature. LU_2004_ATF4_select: Table 1 from Lu *et al*. [24]. ADACHI_2008_ATF6: Table 1 from Adachi *et al*. [16]. LU_2004_ATF4_all: Additional file 2: Table S1 from Lu *et al*. [24]. ACOSTA_ALVEAR_2007_XBP1: Table S5 from Acosta-Alvear *et al*. [25]. SIZE = number of genes within set; NES = Normalized Enrichment Score; q-value = FDR-adjusted *P*-value.

We have shown before that simultaneous ablation of SREBP1 and SREBP2 expression prevents Akt-dependent cell growth [7]. To further investigate the role of SREBPs in Akt-mediated cell growth, we made use of an immortalized human retinal pigment epithelial cell line expressing an inducible version of the Akt kinase (RPE-hTERTmyrAkt-ER). Cells were placed into medium supplemented with 1% lipoprotein-deficient serum (lipoprotein-deplete conditions) for 24 hours. This condition has been optimized to study Akt-dependent SREBP activation in these cells [7,23]. We analyzed global changes in gene expression in response to single or combined depletion of SREBP1 and SREBP2 using microarrays. We identified approximately 400 genes that were regulated by SREBP1 and SREBP2 in a cooperative manner (Figure 1A; Additional file 2: Table S1). Genes that were regulated more than two-fold in response to combined SREBP1 and 2 silencing are listed in Table 1. We confirmed the differential expression of selected upregulated and downregulated genes by quantitative reverse transcriptase PCR (qRT-PCR) (Additional file 3: Figure S1). Notably, the majority of genes repressed in response to SREBP depletion correspond to established SREBP target genes, including stearoyl-CoA desaturase (SCD), low-density lipoprotein receptor (LDLR), fatty acid synthase (FASN) and ATP-citrate lyase (ACLY) (Table 1). Pathway analysis (GeneGo, Metacore, Thomson Reuters Scientific Inc, Philadelphia, PA, USA) confirmed that the downregulated genes are strongly associated with SREBP transcription factors (Figure 1B).

**Table 1:** Genes regulated in response to combined silencing of SREBP1 and SREBP2

**symbol**	**siBP1 EtOH**	**siBP1 4-OHT**	**siBP2 EtOH**	**siBP2 4-OHT**	**siBP1+2 EtOH**	**siBP1+2 4-OHT**	**genename**
PTGS2	1.41	1.77	1.40	1.24	49.64	27.66	prostaglandin-endoperoxide synthase 2 (prostaglandin G/H synthase and cyclooxygenase)
SERPINE1	2.71	2.36	2.48	3.90	7.18	11.42	serpin peptidase inhibitor, clade E (nexin, plasminogen activator inhibitor type 1), member 1
DDIT3	−1.38	−1.25	1.14	1.11	7.34	10.51	DNA-damage-inducible transcript 3
FOSB	−1.12	1.03	1.57	1.59	8.98	9.31	FBJ murine osteosarcoma viral oncogene homolog B
BMP2	1.83	1.68	2.52	2.20	13.38	8.41	bone morphogenetic protein 2
PPP1R15A	−1.06	1.26	1.58	1.56	5.90	7.54	protein phosphatase 1, regulatory (inhibitor) subunit 15A
SGK1	3.05	2.82	3.25	2.47	9.88	7.18	serum/glucocorticoid regulated kinase 1
IL11	1.29	1.68	1.65	2.52	5.43	6.94	interleukin 11
DUSP1	1.03	1.09	1.07	1.29	4.69	6.73	dual specificity phosphatase 1
RCAN1	1.23	1.43	−1.02	1.10	6.34	6.71	regulator of calcineurin 1
E2F7	1.92	1.38	2.62	2.23	6.93	6.49	E2F transcription factor 7
VEGFA	1.89	2.60	1.96	2.75	5.28	6.44	vascular endothelial growth factor A
ITPRIP	1.80	1.65	1.38	1.51	6.38	6.23	inositol 1, 4, 5-triphosphate receptor interacting protein
ATF3	−1.46	−1.97	−1.30	−1.93	8.09	6.23	activating transcription factor 3
TRIB3	1.09	1.08	1.09	−1.09	4.61	6.11	tribbles homolog 3 (Drosophila)
PLEKHF1	1.04	1.39	1.28	1.37	3.67	6.01	pleckstrin homology domain containing, family F (with FYVE domain) member 1
PLAT	2.88	1.97	2.38	1.23	10.41	5.50	plasminogen activator, tissue
RCAN1	1.06	1.12	−1.13	−1.03	5.69	5.34	regulator of calcineurin 1
ASNS	−1.10	−1.15	−1.16	−1.32	4.54	5.30	asparagine synthetase
INHBE	1.05	1.07	−1.03	1.05	3.06	5.09	inhibin, beta E
JUN	1.47	1.38	2.00	2.01	4.23	5.08	jun oncogene
CTH	−1.33	−1.02	−1.29	1.21	3.22	4.91	cystathionase (cystathionine gamma-lyase)
HERPUD1	−1.01	1.16	−1.25	−1.12	3.48	4.79	homocysteine-inducible, endoplasmic reticulum stress-inducible
MTHFD2	1.68	1.43	1.52	1.65	4.22	4.75	methylenetetrahydrofolate dehydrogenase (NADP+dependent) 2
IL6	2.02	2.29	1.40	1.71	6.66	4.63	interleukin 6 (interferon, beta 2)
NDRG1	−1.04	−1.05	1.21	1.03	4.07	4.30	N-myc downstream regulated 1
CREB5	1.40	1.35	1.65	1.45	4.56	4.25	cAMP responsive element binding protein 5
ETS2	−1.18	1.43	1.05	1.20	2.82	4.02	v-ets erythroblastosis virus E26 oncogene homolog 2 (avian)
ZNF295	1.03	1.28	1.25	1.41	2.70	3.77	zinc finger protein 295
IL1A	1.17	1.53	1.14	−1.03	3.40	3.75	interleukin 1, alpha
GPT2	−1.18	1.24	1.08	1.24	2.29	3.71	glutamic pyruvate transaminase (alanine aminotransferase) 2
SLC3A2	1.53	1.22	1.06	−1.34	4.21	3.69	solute carrier family 3 (activators of dibasic and neutral amino acid transport) member 2
IRAK2	1.74	1.58	1.55	1.38	5.59	3.62	interleukin-1 receptor-associated kinase 2
CEBPG	1.19	1.12	1.24	1.12	3.65	3.46	CCAAT/enhancer binding protein (C/EBP), gamma
MTHFD2	1.46	1.28	1.32	1.46	4.18	3.42	methylenetetrahydrofolate dehydrogenase (NADP+dependent) 2
IER3	1.42	1.20	1.68	1.29	5.55	3.36	immediate early response 3
ETV5	1.56	1.53	1.41	1.60	3.15	3.34	ets variant 5
ITGA2	1.64	1.40	1.80	1.39	4.53	3.32	integrin, alpha 2 (CD49B, alpha 2 subunit of VLA-2 receptor)
GEM	−1.11	−1.31	−1.02	−1.53	4.81	3.29	GTP binding protein overexpressed in skeletal muscle
DNAJB9	−1.19	1.04	−1.36	−1.27	3.73	3.22	DnaJ (Hsp40) homolog, subfamily B, member 9
NOV	1.06	1.37	−1.04	1.11	2.39	3.17	nephroblastoma overexpressed gene
FICD	1.39	1.34	1.21	1.26	2.63	3.16	FIC domain containing
NOG	1.24	−1.00	1.28	1.50	4.02	3.09	noggin
ST3GAL6	1.06	1.42	1.26	1.34	2.95	3.07	ST3 beta-galactoside alpha-2, 3-sialyltransferase 6
NFIL3	1.25	1.13	1.31	1.12	3.56	3.05	nuclear factor, interleukin 3 regulated
IL1B	1.58	1.35	2.01	1.55	7.94	3.00	interleukin 1, beta
GEM	−1.01	−1.00	1.11	−1.03	1.89	2.88	GTP binding protein overexpressed in skeletal muscle
SLC7A1	1.05	1.05	1.09	1.27	2.37	2.87	solute carrier family 7 (cationic amino acid transporter, y+ system), member 1
SGIP1	−1.13	1.04	1.46	1.50	3.23	2.84	SH3-domain GRB2-like (endophilin) interacting protein 1
SRPK2	1.05	1.11	1.02	1.06	2.37	2.83	SFRS protein kinase 2
CEBPB	−1.30	−1.21	1.04	−1.06	3.21	2.79	CCAAT/enhancer binding protein (C/EBP), beta
DUSP10	−1.02	−1.14	1.32	1.13	2.76	2.76	dual specificity phosphatase 10
C9orf150	1.33	1.48	1.09	−1.15	3.85	2.74	chromosome 9 open reading frame 150
SLC3A2	1.23	−1.13	−1.19	−1.28	3.05	2.66	solute carrier family 3 (activators of dibasic and neutral amino acid transport), member 2
SLC6A15	1.12	1.20	1.21	1.22	2.99	2.56	solute carrier 6 (neutral amino acid transporter), member 15
NCOA7	−1.07	1.13	−1.07	1.01	2.70	2.53	nuclear receptor coactivator 7
TGIF1	−1.28	−1.04	−1.15	−1.01	2.08	2.51	TGFB-induced factor homeobox 1
RND3	−1.45	1.03	−1.34	−1.17	2.49	2.49	Rho family GTPase 3
CBS	1.11	1.04	1.02	1.03	2.11	2.43	cystathionine-beta-synthase
NFKBIZ	−1.15	−1.07	1.00	1.02	2.01	2.43	nuclear factor of kappa light polypeptide gene enhancer in B-cells inhibitor, zeta
STX3	−1.43	−1.43	−1.12	−1.08	1.89	2.42	syntaxin 3
SMOX	1.35	1.14	1.24	1.07	2.78	2.34	spermine oxidase
SAMD4A	1.25	−1.18	1.09	1.05	2.03	2.22	sterile alpha motif domain containing 4A
CLCN7	−1.08	1.23	1.06	1.31	1.72	2.21	chloride channel 7
MXD1	1.20	1.14	1.09	−1.02	2.12	2.20	MAX dimerization protein 1
ADAMTS1	−1.32	−1.24	−1.39	1.14	2.51	2.16	ADAM metallopeptidase with thrombospondin type 1 motif, 1
ASNS	−1.06	−1.02	−1.22	−1.09	2.17	2.15	asparagine synthetase
SIAH2	1.16	1.12	1.04	1.20	1.84	2.13	seven in absentia homolog 2 (Drosophila)
SQSTM1	1.20	−1.13	1.12	1.03	2.00	2.13	sequestosome 1
CCNL1	−1.03	1.04	1.16	1.07	1.87	2.12	cyclin L1
SLC38A1	1.27	1.27	1.47	1.20	1.81	2.12	solute carrier family 38, member 1
HMOX1	1.06	−1.26	−1.27	−1.69	2.22	2.11	heme oxygenase (decycling) 1
SYVN1	1.20	1.17	1.20	1.26	2.55	2.08	synovial apoptosis inhibitor 1, synoviolin
CTH	−1.08	−1.07	−1.03	−1.03	1.72	2.07	cystathionase (cystathionine gamma-lyase)
SLC25A25	1.21	1.06	1.19	1.17	2.03	2.01	solute carrier family 25 (mitochrondrial carrier; phosphate carrier), member 25
FZD2	−1.01	−1.16	1.14	1.04	−1.62	−2.08	frizzled homolog 2 (Drosophila)
ADIPOR2	−1.05	−1.18	−1.02	−1.04	−1.93	−2.23	adiponectin receptor 2
ENC1	−1.02	−1.15	1.08	1.21	−2.00	−2.81	ectodermal-neural cortex (with BTB-like domain)
B3GALNT1	−1.40	−1.48	−1.70	−1.43	−3.00	−3.92	beta-1, 3-N-acetylgalactosaminyltransferase 1 (globoside blood group)
PPP1R3C	−1.23	−1.42	−1.26	−1.15	−5.14	−4.31	protein phosphatase 1, regulatory (inhibitor) subunit 3C
MT1F	−10.78	−8.64	−1.37	−1.26	−3.87	−4.34	metallothionein 1F
FADS2	−1.58	−1.55	−2.18	−1.61	−4.42	−4.77	fatty acid desaturase 2
ACLY	−2.36	−2.47	−2.02	−1.95	−4.87	−5.73	ATP citrate lyase
FADS1	−2.02	−2.05	−2.29	−1.58	−5.53	−6.42	fatty acid desaturase 1
FASN	−1.36	−1.23	−2.71	−1.76	−6.32	−6.96	fatty acid synthase
SLC25A1	−1.53	−2.67	−2.42	−2.79	−5.35	−7.44	solute carrier family 25 (mitochondrial carrier; citrate transporter), member 1
LPIN1	−1.96	−1.83	−2.77	−2.24	−6.16	−8.24	lipin 1
INSIG1	−1.76	−1.91	−4.47	−2.98	−9.60	−11.16	insulin induced gene 1
LDLR	1.19	1.20	−2.09	−1.23	−13.16	−11.20	low density lipoprotein receptor
LSS	−1.35	−1.90	−5.08	−3.10	−10.77	−12.52	lanosterol synthase (2, 3-oxidosqualene-lanosterol cyclase)
DHCR7	1.01	−1.25	−4.74	−2.93	−14.01	−15.03	7-dehydrocholesterol reductase
SCD	−1.45	−1.40	−2.35	−1.79	−16.93	−16.08	stearoyl CoA desaturase (delta-9-desaturase)

List shows genes identified by microarray analysis as regulated by combined silencing of SREBP1 and SREBP2. Genes were selected to show at least two-fold difference in expression after combined silencing of SREBP1 and SREBP2 compared to control transfected cells or cells transfected with either SREBP1 or SREBP2 targeting siRNAs. Values represent fold-change relative to control-transfected cells treated with ethanol (EtOH) or 4-hydroxytamoxifen (4-OHT), respectively, and are the results of three independent experiments.

A large number of genes showed considerable induction of expression following combined depletion of SREBP1 and SREBP2. Interestingly, many of these genes seem to be linked to inflammation and stress response such as cyclooxygenase 2 (PTGS2/COX2), c-JUN and several interleukins. We also found induction of several genes linked to ER-stress and the UPR (Table 1) and targets of the ATF4, XBP-1 and c-Jun transcription factors strongly associated with genes induced following SREBP depletion (Figure 1B). As the three main transcription factors associated with the ER-stress are ATF4, ATF6 and XBP-1, we compared the results of our microarray analysis with published datasets of target genes for ATF4 [24], ATF6 [16] and XBP-1 [25] using gene set enrichment analysis (GSEA). This analysis suggested that transcriptional programs associated with ER-stress are induced in response to combined ablation of SREBP1 and 2 (Figure 1C, D).

### Ablation of SREBP1 and SREBP2 causes ER-stress and activates the UPR

**Figure 2:**
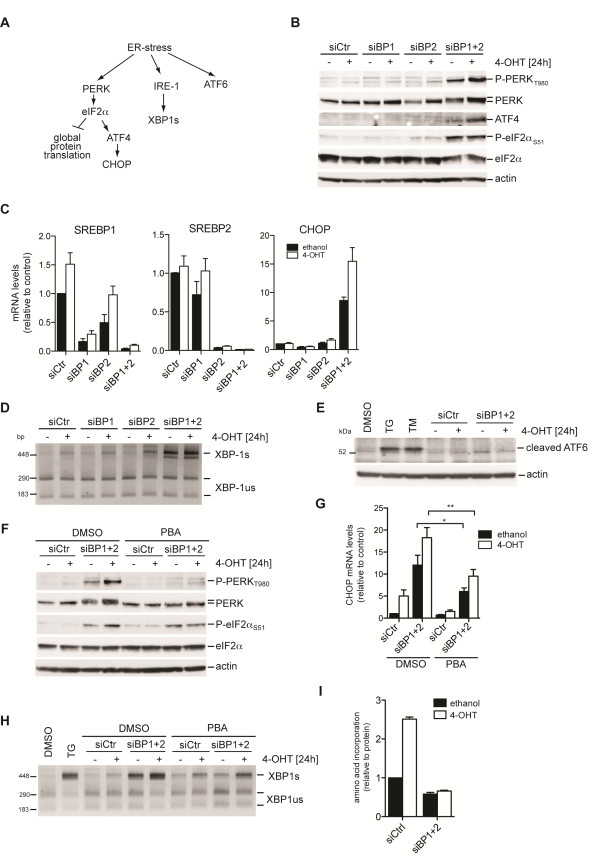
**Inhibition of SREBP function induces ER-stress. **(**A**) Schematic overview of the ER-stress pathway. (**B**) RPE-myrAkt-ER cells were transfected with siRNA targeting SREBP1 (siBP1), SREBP2 (siBP2) or both (siBP1 + 2). Scrambled siRNAs were used as controls (siCtr). At 72 hours post-transfection, cells were placed in medium containing 1% LPDS and treated with 100 nM 4-OHT or solvent (ethanol) for 24 hours. Phosphorylation of PERK (threonine 980) and eIF2α (serine 51) was determined. Actin was used as a loading control. (**C**) cDNA from cells treated as in B was analyzed for expression of *SREBP1*, *SREBP2 *and C/EBP-homologous protein (*CHOP*) by quantitative reverse transcriptase PCR (qRT-PCR). Graphs show mean ± standard error of the mean (SEM) of three independent replicates. (**D**) Splicing of *XBP-1* was determined by RT-PCR. Bands representing the unspliced (XBP-1us) and spliced transcript (XBP-1 s) are marked. (**E**) Cleaved ATF6 (50 kDa) was detected by immunoblotting. Treatment with 50 nM thapsigargin (TG) or 6 μM tunicamycin (TM) was used as control. (**F**) Cells depleted of SREBP1 and SREBP2 were treated with 100 nM 4-OHT or 10 mM of 4-phenyl butyric acid (PBA) for 24 hours as indicated. Phosphorylation of PERK and eIF2α was determined. (**G**) *CHOP* expression in cells treated in parallel to F. Graphs show mean ± (SEM) of three independent replicates. (**H**) Effect of PBA treatment on *XBP-1 *splicing. 50 nM thapsigargin (TG) was used as control. (**I**) Effect of SREBP depletion on protein synthesis. Graph shows mean and range of two independent experiments. *P < 0.05; **P < 0.01.

Since our analysis suggested that SREBP ablation induces changes in gene expression associated with the UPR, we next investigated whether this change is associated with activation of the ER-stress kinase PERK (Figure 2A). We found that combined silencing of SREBP1 and SREBP2 in cells cultured in lipoprotein-deplete conditions resulted in a strong increase in PERK phosphorylation compared to transfection of a non-specific control siRNA or silencing of either SREBP isoform alone (Figure 2B). We also observed an increase in phosphorylation of the PERK substrate eIF2α as well as increased translation of ATF4 (Figure 2B), two hallmarks of the ER-stress pathway. Silencing of SREBP also induced expression of *CHOP*, a transcriptional target of ATF4 (Figure 2C). The same results were also observed when different individual siRNA sequences targeting SREBP1 and SREBP2 were used (Additional file 4: Figure S2A and B).

Silencing of SREBP also induced the splicing of *XBP-1* mRNA (Figure 2D), indicating that inhibition of SREBP induces activation of IRE1. However, we did not observe processing of ATF6 following SREBP inhibition despite ATF6 being cleaved in these cells following treatment with tunicamycin or thapsigargin, two chemical inducers of ER-stress known to activate ATF6 cleavage (Figure 2E). Since many of the targets of ATF6 are also regulated by activation of the other arms of the ER-stress pathway, the regulation of ATF6 target genes observed in the gene expression signature (Figure 1C, D) is likely to be caused by activation of ATF4 or XBP-1.

PBA (4-phenyl butyric acid) is a chemical chaperone that can stabilize proteins in their native conformation and improve the folding capacity of the ER [26]. Treatment with PBA completely blocked phosphorylation of PERK in response to SREBP depletion and reduced phosphorylation of eIF2α following Akt activation (Figure 2F). Moreover, induction of *CHOP* mRNA expression and *XBP-1* splicing was significantly reduced by PBA treatment (Figure 2G, H) indicating that accumulation of misfolded proteins is involved in the induction of ER-stress in response to SREBP ablation.

We observed that activation of Akt in SREBP-depleted cells resulted in a marked increase in the levels of phosphorylated PERK (Figure 2B). Furthermore, induction of ATF4 and *CHOP* was also augmented by Akt activation (Figure 2B, C). These findings suggest that activation of Akt enhances ER-stress in the absence of SREBP. Akt induces translation via the mTORC1 pathway and could increase the protein load of the ER. Indeed, activation of Akt resulted in a two-fold increase in protein synthesis (Figure 2I). Crucially, Akt-dependent induction of protein synthesis was completely abolished in cells depleted of SREBP1 and 2, most likely due to the phosphorylation of eIF2α.

These findings indicate that depletion of SREBP induces two of the three arms of the UPR pathway, potentially by inducing the accumulation of misfolded proteins within the ER, resulting in an inhibition of Akt-dependent protein synthesis.

### Ablation of SREBP function alters cellular lipid composition

We next investigated whether inhibition of fatty acid or cholesterol biosynthesis following SREBP depletion could be responsible for induction of ER-stress. We used inhibitors of fatty acid synthase (C75 and cerulenin) or cholesterol synthesis (compactin) and compared their effect with a chemical inhibitor of SREBP function (fatostatin). Treatment of parental RPE cells with fatostatin in lipoprotein-deplete conditions induced eIF2α phosphorylation after 1 hour and resulted in detectable PERK phosphorylation and a clear shift in its mobility after 3 hours (Additional file 5: Figure S3A). This corresponds to the time when inhibition of SREBP-dependent gene expression by this drug is observed (Additional file 5: Figure S3B). In contrast, treatment with C75, cerulenin or compactin only caused a small increase in eIF2α phosphorylation and failed to induce PERK phosphorylation (Additional file 5: Figure S3A). Silencing of FASN, ACLY, HMGCR or HMGCS failed to cause *CHOP* induction suggesting that inhibition of fatty acid or cholesterol biosynthesis is not sufficient to induce ER-stress (Additional file 5: Figure S3C, D, E).

SREBP-target genes also include enzymes that are involved in lipid modification, mostly the desaturation of newly synthesized fatty acids. Indeed, among the genes most strongly downregulated in response to combined silencing of SREBP1 and SREBP2 were several fatty acid desaturases (SCD, FADS1 and FADS2; Table 1).

**Figure 3:**
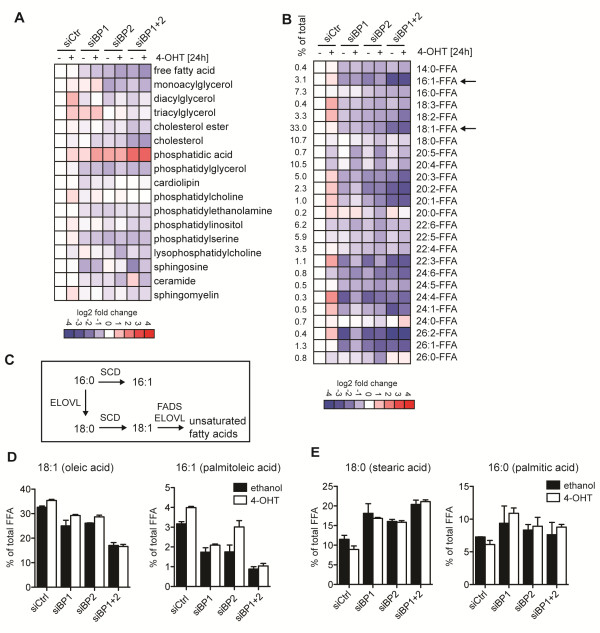
**Depletion of SREBP alters the cellular lipid spectrum and causes loss of mono-unsaturated fatty acids. **(**A**) Lipid analysis of cells depleted of SREBP1 (siBP1) or SREBP2 (siBP2) either alone or in combination (siBP1 + 2) and treated with 100 nM 4-OHT or solvent (ethanol) for 24 hours in medium containing 1% LPDS. Heat map represents log 2 fold changes in concentrations of the different lipid species relative to control-transfected cells (siCtr) treated with solvent (ethanol) (see Additional file 6: Table S2 for complete dataset). (**B**) Heat map representing changes in free fatty acid species. The percentage of each fatty acid in the control sample is also indicated (% of total). Arrows indicate palmitoleic and oleic acid (see Additional file 7: Table S3 for complete dataset). (**C**) Diagram showing the synthetic pathway for the generation of unsaturated fatty acids. Desaturation of C:16 and C:18 fatty acids by stearoyl-CoA desaturase (SCD) is the rate-limiting step. (**D**) Graphs showing the changes in the two major mono-unsaturated fatty acids, oleic and palmitoleic acid, following SREBP depletion represented as percentage of total free fatty acids (FFA). Graphs show mean and range of two independent experiments. (**E**) Changes in the two major saturated fatty acids, stearic and palmitic acid, following SREBP depletion represented as percentage of total free fatty acids (% of FFA). Graphs show mean and range of two independent experiments. ELOVL, long-chain fatty-acyl elongase; FADS, fatty acid desaturase.

We hypothesized that SREBP depletion could alter the cellular lipid composition by blocking lipid desaturation. We therefore investigated the effect of SREBP depletion on cellular lipid composition using mass spectrometry. Activation of Akt in cells cultured under lipoprotein-deplete conditions caused as much as a two-fold increase in the overall amounts of diacylglycerides and triacylglycerol (Figure 3A; Additional file 6: Table S2). Silencing of either SREBP1 or SREBP2 caused a moderate reduction in several lipid classes including ceramide, sphingosine, phosphatidylglycerol and free fatty acids (Figure 3A; Additional file 6: Table S2). Combined depletion of both genes caused a marked increase in the levels of phosphatidic acid (Figure 3A; Additional file 6: Table S2). Phosphatidic acid is a precursor for the synthesis of triacylglycerides and phospholipids, and its accumulation could be a consequence of reduced synthesis of these lipids. The conversion of phosphatidic acid to diacylglycerol is catalyzed by the phosphatidate phosphatase lipin 1 (LPIN1) [27], one of the genes strongly downregulated following SREBP depletion (Table 1).

We also investigated chain length and saturation levels of the lipid species within each class. The results are represented as percentage of the total lipid amount within each class (Additional file 7: Table S3) and number of double bonds (Additional file 8: Table S4). It should be noted that the mass spectrometry method employed here does not allow the definition of positional isomers. Interestingly, combined silencing of both genes resulted in a marked reduction in the percentage of mono-unsaturated fatty acids within the cellular pool of free fatty acids (Figure 3B). This change in saturation correlates with the induction of ER-stress as it was strongest in the samples from cells depleted of both SREBP1 and SREBP2. Fatty acids are synthesized by the condensation of malonyl-CoA with a growing acyl-chain by FASN. The rate-limiting step in the synthesis of unsaturated fatty acids is catalyzed by SCD, which introduces double-bonds into the 9 position of C16:0 (palmitic acid) and C18:0 (stearic acid). Long-chain poly-unsaturated fatty acids are produced from C18:1 (oleic acid) by elongases and other desaturases (Figure 3C). Several enzymes involved in the synthesis of poly-unsaturated fatty acids, including SCD, FADS1 and FADS2, are strongly downregulated in response to SREBP depletion (Table 1).

We found that oleic acid was the most abundant free fatty acid and constitutes approximately 30% of the total pool of free fatty acids in control cells (Additional file 6: Table S2). Interestingly, SREBP depletion caused a two-fold reduction in the percentage of oleic acid compared to control silenced cells (Figure 3D). Palmitoleic acid was the second most abundant mono-unsaturated fatty acid in these cells (3 to 4%) and was reduced three-fold upon SREBP depletion (Figure 3D). We also observed a corresponding increase in stearic acid. Indeed, stearic acid constituted about 20% of the total pool of free fatty acids in SREBP depleted cells (Figure 3E). We also noticed a considerable shift from mono- and poly-unsaturated lipid species to saturated forms throughout other lipid classes, most notably ceramide, diacylglycerides, lysophosphatidic acids, phosphatidic acids and triacylglycerides (Additional file 7: Table S3). These results strongly suggest that ablation of SREBP blocks fatty acid desaturation thereby affecting the saturation state of many cellular lipids. Accumulation of saturated lipids is likely to have profound effects on membrane fluidity and could affect the functionality of the ER, Golgi apparatus or components of the secretory pathway and results in accumulation of misfolded proteins and ER-stress.

### Induction of ER-stress following SREBP depletion is blocked by exogenous lipids

**Figure 4:**
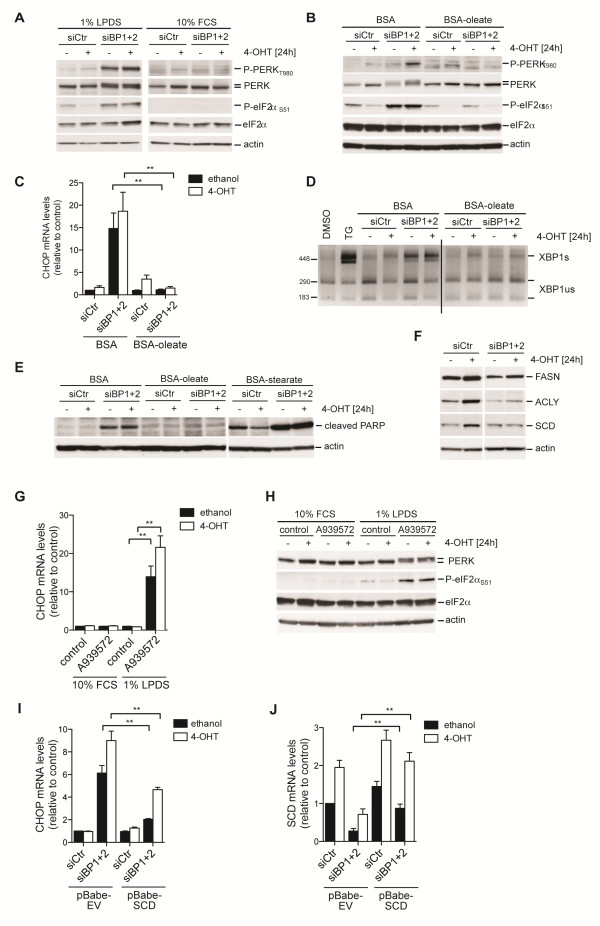
**Induction of ER-stress following depletion of SREBP is blocked by serum lipids or oleate. **(**A**) Cells depleted of SREBP1 and SREBP2 (siBP1 + 2) were placed in medium with 10% FCS or 1% LPDS, treated with 100 nM 4-OHT or solvent (ethanol) for 24 hours. Lysates were analyzed for phosphorylation of PERK and eIF2α. (**B**) Cells were depleted of SREBP1 and SREBP2 and treated with 100 nM 4-OHT or solvent in medium containing 1% LPDS supplemented with BSA or BSA-coupled oleate (300 μM oleate) for 24 hours. Phosphorylation of PERK and eIF2α was determined. (**C**) cDNA from cells treated as in B was used to determine *CHOP* expression by qRT-PCR. Graph shows mean ± SEM of three independent replicates. (**D**) Effect of oleate treatment on *XBP-1 *splicing. Cells treated with 50 nM thapsigargin (TG) were used as control. Line indicates removal of unrelated lanes from scanned gel image. (**E**) Induction of apoptosis (cleaved poly (ADP-ribose) polymerase (PARP)) in cells treated with BSA, BSA-oleate or BSA-stearate (both 300 μM fatty acid). Actin is shown as a loading control. (**F**) Expression of stearoyl-CoA desaturase (SCD) protein following Akt activation and SREBP silencing. (**G**) Parental RPE cells were treated with 1 μM of A939572 in medium with 10% FCS or 1% LPDS. Induction of *CHOP *was determined by qRT-PCR. (**H**) Phosphorylation of PERK (upper band) and eIF2α in cells treated with A939572 as in G. (**I**) Effect of SREBP depletion on *CHOP *induction was determined in empty vector (pBabe-EV) or SCD expressing cells (pBabe-SCD). (**J**) Expression of *SCD *mRNA in empty vector (pBabe-EV) or SCD expressing cells (pBabe-SCD). **P < 0.01.

We next investigated whether ER-stress induced by SREBP depletion could be abolished by restoring cellular mono-unsaturated fatty acids. Phosphorylation of PERK and eIF2α following SREBP depletion, which is readily detected in lipoprotein-deplete conditions, was completely blocked in the presence of 10% fetal calf serum (Figure 4A). In contrast, depletion of SREBP in medium supplemented with 10% fetal calf serum depleted of lipids (LDS) induced PERK phosphorylation (Additional file 9: Figure S4A) suggesting that the lack of serum-derived lipids, but not other serum factors, is responsible for the induction of ER-stress in the absence of SREBP.

Because SREBP depletion reduced the cellular pool of oleic acid, we next investigated the effect of SREBP depletion in cells cultured in lipoprotein-deplete conditions after addition of exogenous oleic acid. Figure 4B shows that addition of fatty acid free BSA-coupled oleic acid completely rescued PERK and eIF2α phosphorylation in SREBP depleted cells both in the presence or absence of Akt activation. BSA-oleate also blocked induction of *CHOP* expression and *XBP-1* splicing in these cells (Figure 4C, D). This suggests that a lack of unsaturated fatty acids is crucial for the induction of ER-stress in these cells.

Because we had also observed an increased fraction of stearic acid within the pool of free fatty acids in SREBP-depleted cells (Figure 3E), we next asked whether addition of stearic acid would be sufficient to induce ER-stress. BSA-stearate caused the appearance of cleaved poly (ADP-ribose) polymerase (PARP), an indicator of apoptosis, even in control cells (Figure 4E). Interestingly, this was partially rescued by activation of Akt, suggesting that Akt counteracts the damage caused by stearic acid. We also observed induction of cleaved PARP in response to SREBP silencing and this was completely prevented by addition of BSA-oleate (Figure 4E). However, addition of BSA-stearate to SREBP-silenced cells enhanced PARP cleavage and caused a substantial loss of viable cells, and prevented the detection of ER-stress markers in these cells (Figure 4E, and data not shown).

Oleic acid is produced by the introduction of a double bond into stearoyl-CoA by SCD. Moreover, SCD expression was strongly inhibited following SREBP depletion (Table 1; Figure 4F). We therefore investigated the effect of SCD inhibition on ER-stress. Transfection of siRNA oligonucleotides targeting SCD did not induce *CHOP* expression (Additional file 9: Figure S4B). However, these oligonucleotides were less efficient in depleting the levels of SCD mRNA compared to silencing of SREBP (Additional file 9: Figure S4C). We therefore used A939572, a specific inhibitor of SCD enzyme activity. Treatment of cells with this compound induced *CHOP* expression and phosphorylation of PERK and eIF2-α only in cells grown under lipoprotein-deplete conditions (Figure 4G, H). Furthermore, re-expression of SCD reduced the induction of the ER-stress marker *CHOP* in cells depleted of SREBP (Figure 4I, J). These results suggest that inhibition of SCD in response to SREBP depletion is responsible for the induction of ER-stress.

### SREBP depletion induces ER-stress via accumulation of reactive oxygen species

The ER-stress pathway is intricately connected to oxidative stress [28]. Protein folding is an oxidative process and excess oxidative stress can affect the folding capacity of the ER. Enhanced levels of ROS have been shown to induce the ER stress pathway [29].

**Figure 5:**
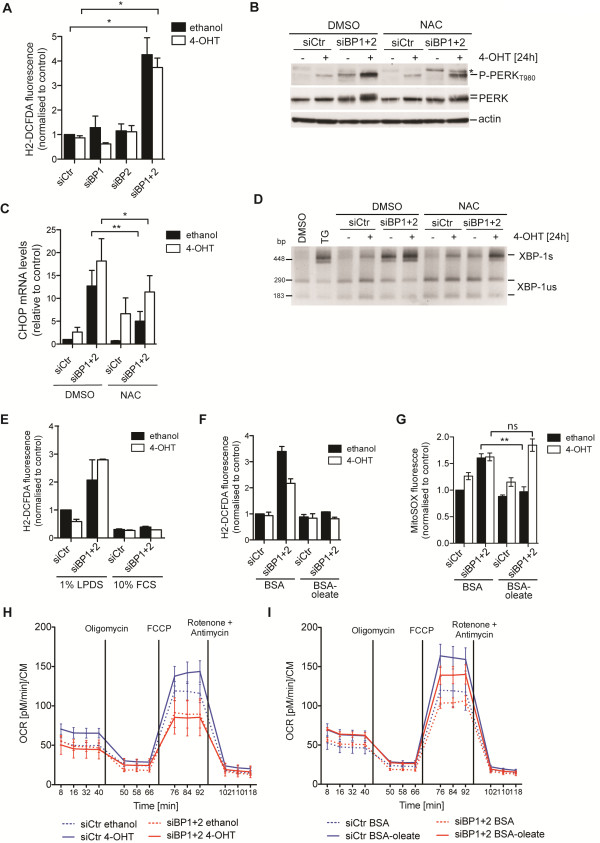
**Depletion of SREBP1 and SREBP2 causes reactive oxygen species (ROS) accumulation. **(**A**) Levels of reactive oxygen species (ROS) in cells depleted of SREBP1 (siBP1) and SREBP2 (siBP2) or both (siBP1 + 2) and treated with 100 nM 4-OHT or solvent for 24 hours in medium with 1% LPDS. Graph shows mean ± SEM of three independent experiments. (**B**) Cells were treated as in A but in the presence or absence of 10 mM of the antioxidant N-acetyl cysteine (NAC). Lysates were analyzed for phosphorylation of PERK (* = unspecific band). (**C**) Expression of *CHOP* in cells treated as in B. Graph shows mean ± SEM of three independent replicates. (**D**) Effect of NAC on *XBP-1 *splicing. Treatment with 50 nM thapsigargin (TG) was used as control. (**E**) ROS levels in SREBP-depleted cells treated with 4-OHT or solvent in medium with 10% FCS or 1% LPDS for 24 hours. Graph shows mean and range of two independent experiments. (**F**) Total ROS levels in cells depleted of SREBP and treated with 4-OHT or solvent in medium containing 1% LPDS supplemented with BSA or BSA-coupled oleate (300 μM oleate) for 24 hours. Graph shows mean and range of two independent experiments. (**G**) Mitochondrial ROS levels in cells treated as in F. Graph shows mean ± SEM of three independent experiments. (**H**) Mitochondrial respiration of control and SREBP depleted cells was determined using a Seahorse Bioanalyzer. Cells were treated with 4-OHT (solid lines) or solvent (dashed lines) for 24 hours in medium with 1% LPDS. Mitochondrial respiratory capacity was determined in the presence of FCCP. (**I**) Mitochondrial respiration after addition of BSA (0.3%, dashed lines) or BSA oleate (300 μM oleate, solid lines). *P < 0.05; **P < 0.01; ns = non-significant.

We therefore investigated whether depletion of SREBP can alter cellular ROS levels. Figure 5A shows that combined silencing of both SREBP1 and SREBP2 resulted in a significant increase in ROS levels. Crucially, this was not further increased following activation of Akt, suggesting that ROS induction is a consequence of SREBP inactivation alone. Activation of Akt under conditions of enhanced ROS levels is likely to increase the demands on the protein folding machinery thereby enhancing the severity of ER-stress. Furthermore, treatment with the antioxidant N-acetyl cysteine (NAC) partially rescued the induction of PERK phosphorylation, *CHOP* expression and *XBP-1* splicing in cells depleted of SREBP both in the presence and absence of Akt activation (Figure 5B, C, D). These results suggest that induction of ER-stress following SREBP depletion is caused by an increase in oxidative stress.

SREBP has been linked to resistance to proteotoxic and oxidative stress through the regulation of glucose-6-phosphate dehydrogenase (G6PD) [9,30]. We therefore investigated whether regulation of G6PD plays a role in the induction of ER-stress following SREBP depletion in the system used here. We only observed a small downregulation of G6PD mRNA following combined depletion of SREBP1 and SREBP2 (Additional file 2: Table S1). Furthermore, silencing of G6PD failed to induce *CHOP* expression in RPE-myrAkt-ER cells following Akt activation (Additional file 10: Figure S5A, B). Therefore, it seems unlikely that G6PD has a major role in the induction of ER stress we have observed. Instead, we observed that ROS formation following SREBP depletion was completely blocked in the presence of full serum (Figure 5E) but not lipid-depleted serum (Additional file 9: Figure S4D). Addition of BSA-oleate prevented overall and mitochondrial ROS accumulation in SREBP depleted cells (Figure 5F, G) suggesting that the depletion of mono-unsaturated fatty acids causes oxidative stress in these cells.

We next investigated the effect of SREBP depletion on mitochondrial respiratory activity. We found that basal mitochondrial oxygen consumption and total mitochondrial oxidative capacity are reduced in SREBP depleted cells (Figure 5H) and that both functions could be restored by the addition of BSA-oleate (Figure 5I). Together, these results suggest that alterations in cellular lipid composition following SREBP depletion cause mitochondrial dysfunction leading to increased formation of ROS.

### SREBP function is required to support cancer cell viability and tumor growth

**Figure 6:**
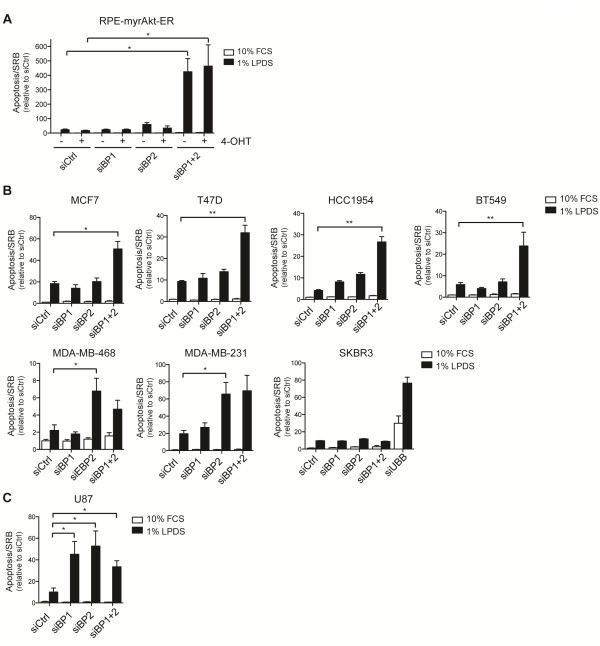
**Induction of apoptosis following depletion of SREBP in cancer cells is restricted to lipoprotein deplete conditions. **(**A**) RPE-myrAkt-ER cells were transfected with 25 nM siRNA oligonucleotides targeting SREBP1, SREBP2 or a combination of both. After 48 hours, cells were placed in medium containing 10% FCS or 1% LPDS for a further 48 hours in the presence of 100 nM 4-OHT or solvent (ethanol). Cell viability was determined by measuring caspase 3/7 activity (Apoptosis) normalized to total protein content (SRB). Graph shows mean ± SEM of three independent experiments. (**B**) The effect of SREBP depletion on cell viability in breast cancer cells. Cells were treated and analyzed as in A. Graphs show mean ± SEM of three independent experiments. Cell lines carry different mutations in components of the PI3-kinase pathway: MCF7 (PIK3CA E545K), T47D (PIK3CA L194F), HCC1954 (PIK3CA H1047R), BT549 (PTEN_null_), MDA-MB-468 (PTEN_null_), MDA-MB-231 (KRAS G13D) and SKBR3 (HER2 amplification). Information on cancer gene mutations was obtained from the Wellcome Trust Sanger Institute Cancer Genome Project (http://www.sanger.ac.uk/genetics/CGP). (**C**) Effect of depletion of SREBP1 or SREBP2 on viability of U87 glioblastoma cells. Graph shows mean ± SEM of three independent experiments. *P < 0.05; **P < 0.01.

The UPR pathway ensures that cells can respond to an excessive load of damaged and misfolded proteins by increasing the protein folding capacity of the ER and inducing ER-associated protein degradation (ERAD) [28]. However, excess and prolonged ER-stress can cause loss of cell viability by inducing apoptosis [31]. Indeed, we found that combined depletion of SREBP1 and SREBP2 induced apoptosis in RPE-myrAkt-ER cells only in lipoprotein-deplete conditions (Figure 6A). Activation of Akt did not rescue the induction of apoptosis by SREBP silencing (Figure 6A).

The Akt/mTORC1 pathway is frequently deregulated in human cancer [32]. We therefore investigated the effect of SREBP depletion in a panel of human cancer cell lines. Combined silencing of SREBP1 and SREBP2 caused apoptosis in four breast cancer cell lines (MCF7, BT549, T47D and HCC1954, Figure 6B). In contrast, silencing of SREBP2 was sufficient to induce apoptosis in MDA-MB231 and MDA-MB468 cells, while SKBR3 were insensitive to SREBP depletion (Figure 6B). Interestingly, all cell lines that were sensitive to SREBP ablation show mutations in a component of the PI3-kinase pathway (PTEN, PIK3CA or KRAS; COSMIC cancer cell line project), while the insensitive SKBR3 cell line is wild type for these genes. This suggests that SREBP may be essential for cancer cells that have activated this signaling axis.

Human glioblastoma multiforme (GBM) is strongly associated with mutations within the PI3-kinase pathway [33]. We therefore investigated the effect of SREBP depletion in U87 glioblastoma cells. Interestingly, these cells were sensitive to ablation of either SREBP1 or SREBP2 suggesting that both transcription factors could have overlapping but non-redundant functions in these cells (Figure 6C).

**Figure 7:**
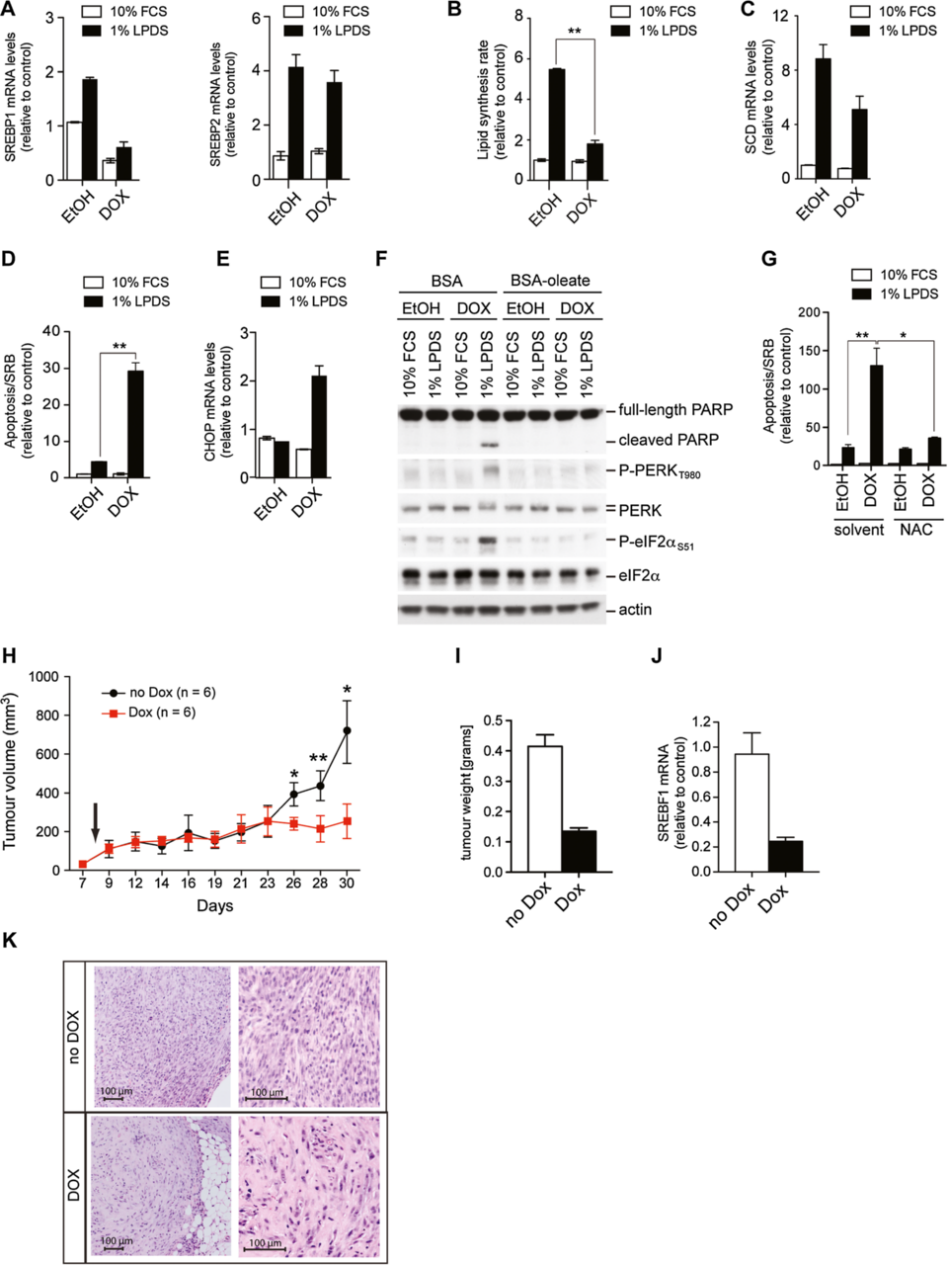
**SREBP1 is essential for cell viability and *in vivo *tumor growth. **(**A**) U87-GFP-Tet-pLKO-shSREBP1 cells were treated with 1 μg/ml doxycycline or solvent (ethanol) for 48 hours before being placed in medium containing either 10% FCS or 1% LPDS for a further 24 hours. Expression of *SREBP1 *and *SREBP2 *was determined. Graphs show mean and range of two independent experiments. (**B**) Cells were treated as in A and acetate-dependent *de novo *lipid synthesis was determined. Graph shows mean ± SEM of three independent experiments. (**C**) Expression of stearoyl-CoA desaturase (*SCD*) in U87 cells depleted of SREBP1. Graph shows mean and range of two independent experiments. (**D**) Induction of apoptosis was determined in cells depleted of SREBP1. Cells were treated with 1 μg/ml doxycycline or solvent for 48 hours before being placed in medium containing either 10% FCS or 1% lipoprotein depleted serum (LPDS) for the final 64 hours. Graph shows mean ± SEM of three independent experiments. (**E**) Expression of *CHOP* in cells treated as in A. Graph shows mean and range of two independent experiments. (**F**) Cells were treated with 1 μg/ml doxycycline or solvent for 48 hours before being placed in medium containing either 10% FCS or 1% LPDS for the final 24 hours. Lysates were analyzed for cleaved PARP and PERK and eIF2α phosphorylation. (**G**) Cells were treated as in D but 10 mM NAC was added prior to placing into lipoprotein-deplete conditions. Graph shows mean ± SEM of three independent experiments. (**H**) Nude mice (nu/nu, 6 per group) were injected subcutaneously with 5x10^6 ^U87-GFP-Tet-pLKO-shSREBP1 cells. Silencing was induced in the treatment group by addition of doxycycline to the food (day 8). Tumor volumes were determined over 30 days. Graph shows mean ± SEM. (**I**) Weight of tumors at day 30. Graph shows mean ± SEM. (**J**) Expression of SREBP1 in tumors at day 30. Graph shows mean ± SEM. (**K**) Histological analysis of tumors (hematoxylin and eosin staining). *P < 0.05; **P < 0.01.

Transduction of U87 cells with an inducible lentiviral expression construct encoding short-hairpin RNA (shRNA) targeting the expression of SREBP1 (Tet-pLKO), resulted in specific depletion of SREBP1 expression after doxycycline treatment without affecting the expression of SREBP2 (Figure 7A). Depletion of SREBP1 alone was sufficient to block the induction of lipid synthesis by lipoprotein-depletion and reduced the induction of SCD (Figure 7B, C). Expression of G6PD was not affected by SREBP1 depletion (Additional file 11: Figure S6A).

As expected, stable silencing of SREBP1 induced apoptosis in these cells, restricted to lipoprotein-deplete conditions only (Figure 7D). ER-stress was also induced by the depletion of SREBP1 in U87 cells demonstrated by an increase in *CHOP* expression and phosphorylation of PERK and eIF2α only under lipoprotein-deplete conditions (Figure 7E, F). Crucially, addition of exogenous oleic acid rescued the induction of ER-stress and cell death as indicated by cleavage of PARP, in the SREBP1-depleted cells (Figure 7F). Treatment with the antioxidant NAC was sufficient to block apoptosis in U87 cells where SREBP1 levels have been ablated (Figure 7G). Expression of *SREBP1*, *SREBP2*, *SCD* and *CHOP* or levels of apoptosis were not affected by doxycycline treatment in U87 cells expressing a scrambled shRNA sequence (Additional file 11: Figure S6B, C). Together, these data indicate that loss of SREBP1 in U87 cells is sufficient to induce ER-stress and apoptosis, mediated by loss of unsaturated fatty acids and accumulation of ROS.

To investigate the role of SREBP1 in supporting the growth and survival of cancer cells under the conditions encountered by a growing tumor *in vivo*, we injected U87-Tet-pLKO-shSREBP1 cells into the dorsal flank of nude mice (nu/nu). After tumors were palpable (8 days), mice were divided into two groups, and one group was treated with doxycycline. Tumor growth was followed over 30 days. Depletion of SREBP1 caused a significant reduction in tumor volume and weight (Figure 7H, I). When we investigated the efficiency of gene ablation *in vivo*, we observed a 70 to 80% reduction in SREBP1 mRNA levels after doxycycline treatment (Figure 7J). Histological analysis revealed a reduced density of tumor cells in the doxycycline-treated cohort associated with increased amounts of stromal cells consistent with the reduction in tumor growth (Figure 7K). These results confirm that SREBP is essential for the growth and survival of cancer cells under physiological conditions.

## Discussion

Protein folding and maturation is an important function of the ER and essential for cell viability. Chaperones and folding enzymes that ensure the correct trafficking and quality control of newly synthesized polypeptide chains are localized to the ER lumen. Accumulation of misfolded proteins following inhibition of protein folding, glycosylation or transport induces the unfolded protein response pathway, a highly regulated stress response cascade that increases the capacity of the ER to cope with the excess protein load. To elucidate the role of lipid metabolism in the regulation of cell growth, we analyzed the effect of SREBP depletion in immortalized human epithelial cells cultured under lipoprotein-deplete conditions. These conditions ensure that cells rely mainly on *de novo* lipid synthesis as the uptake of lipoproteins and free fatty acids from the medium is minimized. We observed that depletion of SREBP induces a transcriptional signature indicative of ER-stress and the UPR pathway. SREBP depletion activates the ER-stress kinase PERK resulting in increased phosphorylation of eIF2α. This was blocked by the chemical chaperone PBA suggesting that induction of PERK following SREBP depletion is caused by misfolded proteins. SREBP depletion also induced splicing of *XBP-1* mRNA suggesting that the IRE1 arm of the ER-stress pathway is engaged. However, although we observed ATF6 target genes as part of the gene signature induced following SREBP depletion, cleavage of the ATF6 protein was not detected. This could be explained by the substantial overlap between the transcriptional programs regulated by the different arms of the ER-stress response as many ER-stress target genes, including *CHOP*, are regulated by both ATF4 and ATF6 [16,19,34,35].

Interestingly, induction of PERK and eIF2α phosphorylation was enhanced by Akt activation. It has been shown previously that aberrant activation of mTORC1 by loss of TSC1 or TSC2 activates the UPR by increasing the protein load in the ER [1,36]. In our cell system, activation of Akt in the presence of SREBP was not sufficient to induce ER-stress. However, induction of the UPR markers was enhanced when Akt was activated in SREBP depleted cells, suggesting that increased protein synthesis aggravates ER-stress when SREBP is absent. It is likely that induction of protein synthesis by the Akt/mTORC1 signaling axis increases the demand for protein folding, trafficking and quality control within the ER. Moreover, depletion of SREBP blocked Akt-dependent protein synthesis, thus implying cross-talk between the protein and lipid synthesis pathways.

We observed downregulation of several enzymes within the fatty acid and cholesterol biosynthesis pathways following SREBP depletion. Previous reports have shown that inhibition of FASN induces ER-stress and loss of viability in breast cancer cells [37]. However, we found that inhibition of fatty acid or cholesterol biosynthesis alone was not sufficient to induce ER-stress in the cell line used here suggesting that additional components of the transcriptional program downstream of SREBP are required to prevent ER-stress. Among the genes most strongly inhibited by combined deletion of both transcription factors in our study were enzymes that catalyze fatty acid desaturation. We found that SREBP depletion caused a reduction in the levels of the unsaturated forms of several major lipid species. Desaturation alters the physical properties of lipids and is likely to have dramatic consequences for the function of structural lipids. Depletion of unsaturated fatty acids decreases the fluidity of the lipid bilayer and is likely to affect many processes that depend on biological membranes, including the synthesis, glycosylation and targeting of proteins. Indeed, inhibition of SCD has been shown to induce *CHOP* expression and apoptosis in cancer cells [38,39]. We found that addition of exogenous oleate or re-expression of SCD was sufficient to prevent ER-stress caused by SREBP depletion. Oleate has also been shown to prevent abnormal lipid distribution and ER-expansion caused by palmitate in skeletal muscle cells [40].

We also found that depletion of SREBP increased cellular ROS levels and impaired mitochondrial respiratory capacity. Importantly, phosphorylation of PERK and splicing of *XBP-1* following SREBP depletion were blocked by antioxidant treatment suggesting that ROS formation is essential for the engagement of this stress response pathway. The mechanism of regulation of the ER-stress response by ROS is only poorly understood but may involve direct activation of PERK [29]. Protein folding by the endoplasmic oxidoreductin 1-like protein (ERO1) is a highly oxidative process [41] and could be impaired under conditions of oxidative stress. Importantly, ROS induction and inhibition of mitochondrial respiratory capacity was abolished by oleate, suggesting that alterations in lipid composition cause mitochondrial dysfunction leading to oxidative stress in SREBP-depleted cells.

Our results also demonstrate that SREBP function is crucial for cell survival in lipoprotein-deplete conditions. Prolonged or excessive ER-stress leads to the induction of apoptosis [31]. Interestingly, the sensitivity to SREBP depletion was not restricted to RPE cells but could also be demonstrated in a panel of breast cancer cell lines in which the PI3-kinase pathway is activated by loss of function of PTEN or activating mutations in PIK3CA or KRAS. Depletion of SREBP1 was sufficient to induce apoptosis in U87 glioblastoma cells *in vitro*, which was restricted to lipoprotein-deplete conditions. However, SREBP1 function was essential for tumor formation suggesting that exogenous lipids are indeed limited under the physiological conditions encountered by cancer cells *in vivo*. Many cancer cells overexpress lipid metabolism enzymes and reactivate *de novo* fatty acid biosynthesis, but the exact mechanisms of this metabolic switch and its advantages for tumor growth are still unclear [42,43]. Enhanced fatty acid biosynthesis, elongation and desaturation are likely to be crucial to fulfill the cellular demand of lipids for membrane biogenesis during cell growth and proliferation. Silencing of acetyl-CoA carboxylase-α (ACACA) inhibits the proliferation of LnCAP prostate cancer cells [44], and inhibition of SREBP2 processing was found to reduce the viability of prostate cancer cells, particularly in lipoprotein deficient serum [45]. Our findings suggest that lipid synthesis and desaturation are also required to support the increased rate of protein synthesis in rapidly proliferating cancer cells. Activation of SREBP by the Akt/mTORC1 pathway may therefore decrease the dependence of cancer cells on exogenous lipids usually provided by the bloodstream. This may be particularly important under conditions of limited access to serum-derived factors such as those present in less vascularized regions of solid tumors.

## Conclusions

This study demonstrates that SREBP is essential for cancer cell survival and has a role in the regulation of lipid metabolism, protein homeostasis, stress response and cellular redox balance. Depletion of SREBP in the absence of exogenous lipids results in reduced levels of unsaturated fatty acids and leads to induction of ER-stress, ROS accumulation and inhibition of global protein synthesis. This suggests that SREBP is required for the coordinated regulation of lipid and protein biosynthesis, two essential processes required for Akt-dependent cell growth. We also found that depletion of SREBP induces apoptosis in a panel of breast cancer cell lines only in the absence of serum lipoproteins. Furthermore, depletion of SREBP1 induced ER-stress and apoptosis in U87 glioblastoma cells and blocked tumor formation in a xenograft model, indicating that extracellular lipids may be a limiting factor for tumor growth *in vivo*.

Taken together, our findings suggest that cellular lipid synthesis and desaturation are essential for the survival of cancer cells under physiological nutrient conditions. It is possible that cancer cells induce SREBP and *de novo* lipid synthesis as a response to the reduced amounts of lipids available within the tumor microenvironment, and that SREBP-dependent lipid synthesis and desaturation become essential for cancer cell growth and survival under these conditions. Targeting these processes could therefore provide novel strategies for cancer treatment.

### Availability of supporting data

The data sets supporting the results of this article are included within the article and in the Additional file 1 supplementary information.

## Abbreviations

ACLY: ATP-citrate lyase; ATF: Activating transcription factor; BSA: Bovine serum albumin; CHOP: C/EBP-homologous protein; DMEM: Dulbecco’s modified Eagle’s medium; EGFR: Epidermal growth factor receptor; eIF2α: eukaryotic translation initiation factor-2 α-subunit; ER: Endoplasmic reticulum; ERAD: Endoplasmic reticulum associated protein degradation; FASN: Fatty acid synthase; FCS: Fetal calf serum; FDR: False discovery rate; GBM: Glioblastoma multiforme; GSEA: Gene-Set Enrichment Analysis; G6PD: Glucose-6-phosphate dehydrogenase; HMGCR: HMG-CoA reductase; HMGCS: HMG-CoA synthase; INSIG1: Insulin-induced gene; IRE1: Inositol-requiring protein-1; ISR: Integrated stress response; LC-MS: Liquid chromatography-mass spectrometry; LDLR: Low-density lipoprotein receptor; LDS: Lipid depleted serum; LPDS: Lipoprotein depleted serum; mTORC1: mammalian target of rapamycin complex 1; OCR: Oxygen consumption rate; PERK: Eukaryotic translation initiation factor 2-alpha kinase 3; qRT-PCR: quantitative reverse transcriptase PCR; ROS: Reactive oxygen species; SCD: Stearoyl-CoA desaturase; SREBP: Sterol regulatory element binding protein; SCAP: SREBP cleavage activating protein; TCA: Trichloracetic acid; TSC1/TSC2: Tuberous sclerosis complex protein 1/2; UPR: Unfolded protein response; XBP-1: X-box binding protein 1; 4-OHT: 4-hydroxy tamoxifen.

## Competing interests

The authors declare that they have no competing interests.

## Authors’ contributions

BG did the microarray analysis, performed most experiments in human retinal pigment epithelial cells and human breast cancer cells and participated in the design of the study and writing of the manuscript; CAL analyzed SREBP in U87 cells, developed the U87 shSREBP cells, participated in the design of the study and contributed to the writing of the manuscript; KB provided the U87 glioblastoma cells and essential conceptual insight; SR performed the *in vivo* studies; QZ performed the lipidomics analysis; ECF analyzed ER-stress in U87 cells and contributed to the writing of the manuscript; SK performed some qPCR analyses; BP analyzed gene expression in U87 cells; HM performed some ROS detection assays; PE analyzed the microarray data; MW analyzed the lipidomics data and helped with coordination of the study; ALH was involved in the design and coordination of the study; AS conceived and coordinated the study and wrote the manuscript. All authors read and approved the final manuscript.

## Supplementary Material

Additional file 1 Supplemental Information. Click here for file

Additional file 2 **Table S1. **List of 416 genes regulated by SREBP1 and SREBP2 in a cooperative manner. Genes identified by Illumina microarray analysis as regulated by combined silencing of SREBP1 and SREBP2 by one-way ANOVA (analysis of variance) of quantile-normalized data using an FDR of 0.01. The columns list signal intensity and fold change over the respective control siRNA treated sample. Data represent three biologically independent experiments. Click here for file

Additional file 3 **Figure S1. **Validation of microarray experiment. RNA from cells after single or combined silencing of SREBP1 and SREBP2 treated with 100 nM 4-OHT or solvent (ethanol) for 24 hours in medium containing 1% lipoprotein deficient serum (LPDS) was used to determine the expression of selected upregulated and downregulated genes. Graph shows mean ± SD of two independent experiments. Click here for file

Additional file 4 **Figure S2. **Silencing of SREBP1 and SREBP2 using different siRNA sequences induces eIF2α phosphorylation and *CHOP *expression. (A) RPE-myrAkt-ER cells were transfected with different combinations of siRNA oligonucleotides specific for SREBP1 (siBP1#1 or siBP1#2) or SREBP2 (siBP2#1 or siBP2#4) or pools of four oligonucleotides targeting either gene (siBP1 + 2 pool). At 72 hours post-transfection, cells were placed into medium supplemented with 1% LPDS and treated with 100 nM 4-OHT or solvent (ethanol) for 24 hours. Lysates were analyzed for expression of SREBP1, SREBP2, phospho eIF2α (serine 51) and total eIF2α by immunoblotting. Actin was used as a loading control. (B) RNA from cells treated in parallel to A was used to determine expression of *SREBP1*, *SREBP2 *and *CHOP *by qRT-PCR. Graphs show mean ± SD of two independent experiments. Click here for file

Additional file 5 **Figure S3. **Inhibition of fatty acid or cholesterol biosynthesis is not sufficient to induce ER-stress. (A) Parental RPE-hTERT cells were placed in medium containing 1% LPDS for 24 hours and treated with 20 μM fatostatin, 45 μM C75, 40 μM cerulenin or 10 μM compactin (mevastatin) for the final 1, 3 or 6 hours or with 50 nM thapsigargin (TG) for the last 6 hours. Whole cell lysates were analyzed for expression and phosphorylation of PERK and eIF2α. (B) Expression of the SREBP target genes *FASN *and *SCD *in cells treated with 20 μM fatostatin for 1, 3 or 6 hours in medium containing 1% LPDS. Graph shows mean ± SD of two independent experiments. (C) RPE-myrAkt-ER cells were transfected with siRNA oligonucleotides targeting the indicated genes. At 72 hours post-transfection, cells were placed into medium supplemented with 1% LPDS and treated with 100 nM 4-OHT or solvent for 24 hours. Expression of *CHOP* was determined by qRT-PCR. Graph shows mean ± SD of two independent experiments. (D) Efficiency of downregulation of SREBP1 and SREBP2 after siRNA transfection was determined by qRT-PCR. Graphs show mean ± SD of two independent experiments. (E) Efficiency of downregulation of each target gene following SREBP depletion or gene-specific siRNA transfection was determined by qRT-PCR. Graphs show mean ± SD of two independent experiments. Click here for file

Additional file 6 **Table S2. **SREBP depletion causes marked changes in cellular lipid composition. Lipid concentrations in RPE-myrAkt-ER were analyzed in cells following silencing of SREBP1 or SREBP2 or after combined ablation of both genes. Cells were placed in medium supplemented with 1% LPDS and treated with 100 nM 4-OHT or solvent (ethanol) for 24 hours. Lipid concentrations were determined by mass spectrometry and normalized to protein concentration. The two values represent biologically independent experiments. Click here for file

Additional file 7 **Table S3. **SREBP depletion causes a shift from unsaturated to saturated lipid species. Composition of the major lipid species was analyzed in RPE-myrAkt-ER cells after silencing of SREBP1 or SREBP2 or after combined ablation of both genes. Cells were placed in medium supplemented with 1% LPDS and treated with 100 nM 4-OHT or solvent (ethanol) for 24 hours. Values represent the amount of a given lipid as % of total lipids within its class. The two values represent biologically independent experiments. Click here for file

Additional file 8 **Table S4. **Saturation levels. Click here for file

Additional file 9 **Figure S4. **Silencing of SREBP1 and SREBP2 induces PERK phosphorylation and ROS in medium supplemented with lipid depleted serum. (A) Cells depleted of SREBP1 and SREBP2 were placed in medium supplemented with 10% lipid depleted serum (LDS), treated with 100 nM 4-OHT or solvent (ethanol) for 24 hours. Lysates were analyzed for phosphorylation of Perk. Actin is used as loading control. (B) RPE-myrAkt-ER cells were transfected with siRNA oligonucleotides targeting SREBP1 and SREBP2 (siBP1+2) or SCD (siSCD). At 72 hours post-transfection, cells were placed into medium supplemented with 1% LPDS and treated with 100 nM 4-OHT or solvent for 24 hours. Expression of *CHOP *was determined by qRT-PCR. Graph shows mean ± range of two independent experiments. (C) Efficiency of downregulation of *SCD *after siRNA transfection was determined by qRT-PCR. Graphs show mean ± range of two independent experiments. (D) Cells treated as in A were used to determine ROS levels by CM-H_2_DCFDA staining and FACS analysis. Graph shows mean ± range of two independent experiments. Click here for file

Additional file 10 **Figure S5. **Silencing of glucose-6-phosphate dehydrogenase does not induce ER-stress. RPE-myrAkt-ER cells were transfected with siRNA oligonucleotides targeting SREBP1 and SREBP2 or glucose-6-phosphate dehydrogenase (G6PD). At 72 hours post-transfection, cells were placed into medium supplemented with 1% LPDS and treated with 100 nM 4-OHT or solvent (ethanol) for 24 hours. Graphs show mean ± SD of two independent experiments. (A) RNA was used to determine expression of *CHOP *by qRT-PCR. (B) Efficient depletion of *G6PD* was determined by qRT-PCR. Click here for file

Additional file 11 **Figure S6. **Expression of *G6PD *in U87 cells after silencing of SREBP1 and controls using a non-targeting shRNA sequence. (A) U87 cells expressing inducible shRNA targeting SREBP1 (U87-shSREBP1) were treated with 1 μg/ml doxycycline or solvent for 48 hours and then placed in medium containing either 10% FCS or 1% LPDS for a further 24 hours. Expression of *G6PD* mRNA was determined by qPCR. Graphs show mean ± SEM of three independent experiments. (B) U87 cells expressing a scrambled shRNA sequence (U87-shScr) were treated as in A. Expression of *SREBP1*, *SREBP2*, *SCD *and *CHOP *was determined by qRT-PCR. Graphs show mean ± SD of two independent experiments. (C) Induction of apoptosis (caspase 3/7 activity) was determined in U87 cells expressing a scrambled shRNA sequence. Cells were treated with 1 μg/ml doxycycline or solvent for 48 hours before being placed in medium containing either 10% FCS or 1% LPDS for a further 48 hours. Graph shows mean ± SEM of three independent experiments. *P < 0.05; n.s. = non significant. Click here for file
