# Supplementary material for: Sterol regulatory element binding protein-dependent regulation of lipid synthesis supports cell survival and tumor growth
Source: Cancer Metab. 2013 Jan 23;1:3. doi: 10.1186/2049-3002-1-3 (PMC3835903; doi:10.1186/2049-3002-1-3)
Supplement: Additional file 1 — Supplemental Information. [file 2049-3002-1-3-S1.doc]

Supplemental Information

**Sterol regulatory element binding protein-dependent regulation of lipid synthesis supports cell survival and tumour growth**

Beatrice Griffiths^1*^, Caroline A. Lewis^1*$^, Karim Bensaad^2^, Susana Ros^1^, Qifeng Zhang^3^, Emma C. Ferber^1^, Sofia Konisti^1#^, Barrie Peck^1^, Heike Miess^1^, Philip East^4^, Michael Wakelam^3^, Adrian L. Harris^2^ and Almut Schulze^1§^

^*^ these authors contributed equally to this work

**Supplementary Figure legends**

**Supplementary Table legends**

**Supplementary methods**

**1) SUPPLEMENTARY FIGURE LEGENDS**

**Figure S1: Validation of microarray experiment**

RNA from cells after single or combined silencing of SREBP1 and SREBP2 treated with 100 nM 4-OHT or solvent (ethanol) for 24 hours in medium containing 1% lipoprotein deficient serum (LPDS) was used to determine the expression of selected up- and down-regulated genes. Graph shows mean ± SD of two independent experiments.

**Figure S2: Silencing of SREBP1 and SREBP2 using different siRNA sequences induces eIF2α phosphorylation and CHOP expression**

**(A)** RPE-myrAkt-ER cells were transfected with different combinations of siRNA oligonucleotides specific for SREBP1 (siBP1#1 or siBP1#2) or SREBP2 (siBP2#1 or siBP2#4) or pools of four oligonucleotides targeting either gene (siBP1+2 pool). 72 hours post-transfection, cells were placed into medium supplemented with 1% LPDS and treated with 100 nM 4-OHT or solvent (ethanol) for 24 hours. Lysates were analysed for expression of SREBP1, SREBP2, phospho eIF2α (serine 51) and total eIF2α by immunoblotting. Actin was used as a loading control.

**(B)** RNA from cells treated in parallel to A was used to determine expression of SREBP1, SREBP2 and CHOP by qRT-PCR. Graphs show mean ± SD of two independent experiments.

**Figure S3: Inhibition of fatty acid or cholesterol biosynthesis is not sufficient to induce ER-stress**

**(A)** Parental RPE-hTERT cells were placed in medium containing 1% LPDS for 24 hours and treated with 20 µM fatostatin, 45 µM C75, 40 µM cerulenin or 10 µM compactin (mevastatin) for the final 1, 3 or 6 hours or with 50 nM thapsigargin (TG) for the last 6 hours. Whole cell lysates were analysed for expression and phosphorylation of PERK and eIF2α.

**(B)** Expression of the SREBP target genes FASN and SCD in cells treated with 20 µM fatostatin for 1, 3 or 6 hours in medium containing 1% LPDS. Graph shows mean ± SD of two independent experiments.

**(C)** RPE-myrAkt-ER cells were transfected with siRNA oligonucleotides targeting the indicated genes. 72 hours post-transfection, cells were placed into medium supplemented with 1% LPDS and treated with 100 nM 4-OHT or solvent for 24 hours. Expression of CHOP was determined by qRT-PCR. Graph shows mean ± SD of two independent experiments.

**(D)** Efficiency of downregulation of SREBP1 and SREBP2 after siRNA transfection was determined by qRT-PCR. Graphs show mean ± SD of two independent experiments.

**(E)** Efficiency of downregulation of each target gene following SREBP depletion or gene-specific siRNA transfection was determined by qRT-PCR. Graphs show mean ± SD of two independent experiments.

**Figure S4: Silencing of SREBP1 and SREBP2 induces PERK phosphorylation and ROS in medium supplemented with lipid depleted serum**

**(A)** Cells depleted of SREBP1 and SREBP2 were placed in medium supplemented with 10% lipid depleted serum (LDS), treated with 100 nM 4-OHT or solvent (ethanol) for 24 hours. Lysates were analysed for phosphorylation of PERK  is used as loading control.

**(B)** RPE-myrAkt-ER cells were transfected with siRNA oligonucleotides targeting SREBP1 and SREBP (siBP1+2) or SCD (siSCD). 72 hours post-transfection, cells were placed into medium supplemented with 1% LPDS and treated with 100 nM 4-OHT or solvent for 24 hours. Expression of CHOP was determined by qRT-PCR. Graph shows mean ± range of two independent experiments.

**(C)** Efficiency of downregulation of SCD after siRNA transfection was determined by qRT-PCR. Graphs show mean ± range of two independent experiments.

**(D)** Cells treated as in A were used to determine ROS levels by CM-H_2_DCFDA staining and FACS analysis. Graph shows mean ± range of two independent experiments.

**Figure S5: Silencing of glucose-6-phosphate dehydrogenase does not induce ER-stress**

RPE-myrAkt-ER cells were transfected with siRNA oligonucleotides targeting SREBP1 and SREBP2 or glucose-6-phosphate dehydrogenase (G6PD). 72 hours post-transfection, cells were placed into medium supplemented with 1% LPDS and treated with 100 nM 4-OHT or solvent (ethanol) for 24 hours. Graphs show mean ± SD of two independent experiments.

**(A)** RNA was used to determine expression of CHOP by qRT-PCR.

**(B)** Efficient depletion of G6PD was determined by qRT-PCR.

**Figure S6: Expression of G6PD in U87 cells after silencing of SREBP1 and controls using a non-targeting shRNA sequence**

**(A)** U87 cells expressing inducible shRNA targeting SREBP1 (U87-shSREBP1) were treated with 1µg/ml doxycycline or solvent for 48 hours and then placed in medium containing either 10% FCS or 1% LPDS for a further 24 h. Expression of G6PD mRNA was determined by qPCR. Graphs show mean ± SEM of three independent experiments.

**(B)** U87 cells expressing a scrambled shRNA sequence (U87-shScr) were treated as in A. Expression of SREBP1, SREBP2, SCD and CHOP were determined by qRT-PCR. Graphs show mean ± SD of two independent experiments.

**(C)** Induction of apoptosis (caspase 3/7 activity) was determined in U87 cells expressing a scrambled shRNA sequence. Cells were treated with 1µg/ml doxycycline or solvent for 48 h before being placed in medium containing either 10% FCS or 1% LPDS for a further 48 h. Graph shows mean ± SEM of three independent experiments. (*) P < 0.05; n.s. = non significant.

**2) SUPPLEMENTARY TABLE LEGENDS**

**Table S1: List of 416 genes regulated by SREBP1 and SREBP2 in a cooperative manner**

Genes identified by Illumina microarray analysis as regulated by combined silencing of SREBP1 and SREBP2 by 1-way ANOVA (analysis of variance) of quantile-normalised data using an FDR of 0.01. The columns list Signal Intensity and Fold Change over the respective control siRNA treated sample. Data represent three biologically independent experiments.

**Table S2: SREBP depletion causes marked changes in cellular lipid composition**

Lipid concentrations in RPE-myrAkt-ER cells following silencing of SREBP1 or SREBP2 or combined ablation of both genes. Cells were placed in medium supplemented with 1% LPDS and treated with 100 nM 4-OHT or solvent (ethanol) for 24 hours. Lipid concentrations were determined by mass spectrometry and normalised to protein concentration. The two values represent biologically independent experiments.

**Table S3: SREBP depletion causes a shift from unsaturated to saturated lipid species**

Composition of the major lipid species in RPE-myrAkt-ER cells after silencing of SREBP1 and SREBP2. Values represent the amount of a given lipid as % of total of its class. The two values represent biologically independent experiments.

**3) SUPPLEMENTARY METHODS**

siRNA sequences

All siRNA sequences were purchased from Dharmacon

Gene name siRNA name product number

siControl si CONTROL NON-Targeting siRNA 3 D-001210-03

SREBP1 SREBPF1 MU-006891

SREBP2 SREBF2 MU-009549

FASN FASN MU-003964

ACLY ACLY MU-004915

HMGCS1 HMGCS1 MU-009808

HMGCR HMGCR MU-009811

PERK EIF2AK3 MU-004883

G6PD G6PD MU-008181

Cell lysis and immunoblotting

For total cell lysis, cells were washed with ice-cold PBS and dissolved in lysis buffer (1% Triton X-100, 50 mM Tris pH 7.5, 300 mM NaCl, 1 mM EGTA, 1 mM DTT, 1 mM NaVO_4_, and Protease- and Phosphatase Inhibitor, Roche). Cell lysates were separated by SDS-PAGE and blotted onto PVDF membrane (Millipore). Proteins were detected by immunoblotting with ECL detection.

RNA preparation and quantitative reverse transcription PCR (qRT-PCR)

Total RNA was isolated using the RNeasy kit (Qiagen). 2.5µg of total RNA was used for cDNA synthesis with SuperScript II RT and oligo dT primer (Invitrogen). qRT-PCR was performed with Quantitect SYBR-Green mastermix (Qiagen). Primers were obtained from Qiagen (Quantitect qRT-PCR primers).

Microarray data analysis

To identify genes regulated in response to combined silencing of SREBP1 and SREBP2, knockdown and control samples for each of the treatment groups were compared by 1-way ANOVA (analysis of variance) of quantile-normalised data with an FDR of 0.01. Genes were further filtered into those significantly affected by silencing of both genes but not by silencing of one. In addition, genes showing a significant change larger than 2-fold after silencing of both genes compared to silencing of either SREBP1 or SREBP2 were also selected. Data analysis and clustering was carried out using the Lumi and Limma packages from Bioconductor 2.6, R 2.11. Pathway analysis and identification of transcription factor targets represented in the set of regulated genes was performed using Metacore GeneGo software.

Lipid analysis by mass spectrometry

Cell pellets were spiked with appropriate internal standards (for each sample: 100ng 12:0/12:0/12:0-TG, 200ng 12:0/12:0-DG, 100ng 12:0-MG, 200ng 17:0-FA, 100ng C17-Cer, 50ng C17-SG, 200ng 14:0/14:0/14:0/14:0-CL, 100ng 12:0/12:0-PG, 200ng 12:0/12:0-PE, 200ng 12:0/12:0-PS, 400ng 17:0/20:4-PI, 100ng 12:0/12:0-PA, 400ng 12:0/12:0-PC, 100ng 17:0-LPA, 100ng 17:0-LPC, 100ng 12:0-Cer1P, 100ng C17-S1P, 200ng C17-SM and 50ng C17-SPC) before extraction. The samples were extracted using a modified Folch method: first extraction with 4ml chloroform : 2ml methanol : 2ml 0.88% NaCl for each sample; second extraction of upper phase with 3ml of synthetic lower phase of chloroform/methanol/0.88% NaCl 2:1:1, the combined lower phases of the lipid extract were dried using a Thermo SpeedVac at room temperature under vacuum and re-dissolved in 50µl chloroform/methanol 1:1, of which 7µl was injected onto the column for LC-MS analysis. For LC/MS/MS analysis, a Shimadzu IT-TOF LC/MS/MS system hyphenated with a five-channel online degasser, four-pump, column oven, and autosampler with cooler Prominence HPLC (Shimadzu) was used. In detail, lipid classes were separated on a normal phase silica gel column (2.1x150mm, 4micro, MicoSolv Technology) using a hexane/dichloromethane/chloroform/methanol/acetanitrile/water/ethylamine solvent gradient based on the polarity of head group. Accurate mass (with mass accuracy ~5ppm) and tandem MS were used for molecular species identification and quantification. The identity of lipid was further confirmed by reference to appropriate lipid standards. IT-TOF mass spectrometer operation conditions: ESI interface voltage +4.5kv for positive ESI and -4kv for negative ESI, heat block temperature 230C, nebulising gass flow 1.4L/min, CDL temperature 210C, with drying gas on at pressure of 100kPa. All solvents used for lipid extraction and LC/MS/MS analysis were LC-MS grade from Fisher Scientific. Lipid amounts were normalised by protein concentrations of each sample.

Generation of doxycycline-inducible shRNA cell line

Short hairpin RNA sequences targeting human SREBF1 or a non-targeting control were cloned into the TetOnPLKO lentiviral vector (Addgene) . Lentiviruses were produced by co-transfecting HEK 293T with the shRNA plasmid and the packaging plasmids pCMVΔR8.91 (gag-pol) and pMD.G (VSV-G glycoprotein) . Supernatants containing lentiviruses were collected 48h after transfection, mixed with polybrene (16 μg/ml) and used to infect U87-GFP cells. Fresh medium containing puromycin (1 μg/ml) was added after 24 hours and cells were selected for at least 48 hours before being used in experiments.

| **Oligonucletotide:** | **Sequence (5’-3’)** |
| --- | --- |
| shSREBF1 forward | CCGGCCAGAAACTCAAGCAGGAGAACTCGAGTTCTCCTGCTTGAGTTTCTGGTTTTT |
| shSREBF1 reverse | AATTAAAAACCAGAAACTCAAGCAGGAGAACTCGAGTTCTCCTGCTTGAGTTTCTGG |
| shScrambled forward | CCGGCCTAAGGTTAAGTCGCCCTCGCTCGAGCGAGGGCGACTTAACCTTAGG |
| shScrambled reverse | AATTCCTAAGGTTAAGTCGCCCTCGCTCGAGCGAGGGCGACTTAACCTTAGG |

Detection of cell viability in cell line panel

All cells were transfected with 25 nM of Dharmacon SMARTpools in 96-well plates. All breast cancer cell lines were transfected using Lullaby (OZ Biosciences). RPE and U87 were transfected using Dharmafect 1 reagent (Dharmacon). After 24 hours, culture medium was replaced. 24 hours later, medium was again replaced with medium containing either 10% FCS or 1% LPDS. 48 hours later, caspase 3/7 activity was measured using a fluorescent substrate (Calbiochem). Cells were fixed with trichloroacetic acid and normalized to total protein content determined by sulforhodamine B staining.

Statistical Analysis

Statistical analysis. Student’s t-tests assuming a two-tailed distribution and equal variance were performed for statistical analysis.

**Supplementary references:**

Wiederschain D, Wee S, Chen L, Loo A, Yang G, Huang A, Chen Y, Caponigro G, Yao YM, Lengauer C et al. 2009. Single-vector inducible lentiviral RNAi system for oncology target validation. *Cell Cycle* **8**: 498-504.

Zufferey R, Nagy D, Mandel RJ, Naldini L, Trono D. 1997. Multiply attenuated lentiviral vector achieves efficient gene delivery in vivo. *Nat Biotechnol* **15**: 871-875.
