# Supplementary material for: Sterol regulatory element binding protein-dependent regulation of lipid synthesis supports cell survival and tumor growth
Source: Cancer Metab. 2013 Jan 23;1:3. doi: 10.1186/2049-3002-1-3 (PMC3835903; doi:10.1186/2049-3002-1-3)
Supplement: Additional file 2 — Table S1. List of 416 genes regulated by SREBP1 and SREBP2 in a cooperative manner. Genes identified by Illumina microarray analysis as regulated by combined silencing of SREBP1 and SREBP2 by one-way ANOVA (analysis of variance) of quantile-normalized data using an FDR of 0.01. The columns list signal intensity and fold change over the respective control siRNA treated sample. Data represent three biologically independent experiments. [file 2049-3002-1-3-S2.pdf]

Table S1: List of 416 genes regulated by SREBP1 and SREBP2 in a cooperative manner

	symbol	Entrez Acc number	Signal Intensity<br>siCtr EtOH	siCtr 4-OHT	siSREBP1 EtOH	siSREBP1 4-OHT	siSREBP2 EtOH	siSREBP2 4-OHT	siSREBP1 +2 EtOH	siSREBP1 +2 4-OHT
	**Upregulated genes**									
ILMN_2054297	PTGS2	5743	146	180	206	319	204	223	7261	4981
ILMN_1744381	SERPINE1	5054	1546	453	4194	1068	3840	1765	11104	5172
ILMN_1676984	DDIT3	1649	375	403	272	323	426	447	2750	4230
ILMN_1751607	FOSB	2354	170	180	152	185	267	286	1530	1673
ILMN_1722718	BMP2	650	138	146	252	246	348	322	1848	1231
ILMN_1659936	PPP1R15A	23645	2103	1879	1988	2370	3314	2940	12408	14172
ILMN_1702487	SGK1	6446	1598	1286	4880	3631	5193	3171	15784	9231
ILMN_1788107	IL11	3589	291	228	375	383	479	576	1578	1586
ILMN_1781285	DUSP1	1843	1274	824	1313	900	1360	1065	5979	5545
ILMN_1712112	RCAN1	1827	263	238	324	339	257	261	1666	1594
ILMN_1798210	E2F7	144455	402	327	772	451	1053	729	2787	2119
ILMN_2375879	VEGFA	7422	186	227	352	591	366	626	984	1462
ILMN_1805192	ITPRIP	85450	698	724	1256	1195	961	1091	4459	4509
ILMN_2374865	ATF3	467	215	338	147	171	165	175	1740	2104
ILMN_1787815	TRIB3	57761	417	564	453	610	454	515	1923	3442
ILMN_1708041	PLEKHF1	79156	244	238	254	330	312	324	894	1427
ILMN_1738742	PLAT	5327	289	274	831	541	688	338	3009	1507
ILMN_2367239	RCAN1	1827	967	771	1027	860	857	752	5496	4113
ILMN_1796417	ASNS	440	958	1093	874	950	825	827	4347	5789
ILMN_1811767	INHBE	83729	103	102	108	108	99	107	314	517
ILMN_1806023	JUN	3725	1333	1149	1964	1584	2667	2313	5643	5840
ILMN_2305112	CTH	1491	264	205	198	202	204	248	849	1006
ILMN_2374159	HERPUD1	9709	909	777	900	903	727	696	3161	3724
ILMN_2405521	MTHFD2	10797	1325	1462	2225	2096	2020	2413	5592	6947
ILMN_1699651	IL6	3569	235	280	474	640	328	477	1567	1297
ILMN_1809931	NDRG1	10397	170	178	163	169	206	183	691	764
ILMN_1731714	CREB5	9586	156	163	218	219	257	237	709	692
ILMN_1720158	ETS2	2114	670	600	570	857	703	720	1892	2415
ILMN_1782110	ZNF295	49854	349	241	359	308	436	340	943	906
ILMN_1658483	IL1A	3552	135	182	158	278	154	177	458	682
ILMN_1684158	GPT2	84706	632	622	537	773	683	772	1447	2310
ILMN_1679041	SLC3A2	6520	517	794	790	965	546	591	2176	2928
ILMN_1745964	IRAK2	3656	265	383	460	604	412	530	1481	1384
ILMN_1716766	CEBPG	1054	334	339	397	379	413	381	1218	1175
ILMN_1674706	MTHFD2	10797	478	538	699	687	629	784	1998	1838
ILMN_1682717	IER3	8870	2242	2861	3186	3428	3763	3695	12455	9618
ILMN_1739222	ETV5	2119	713	557	1115	854	1006	889	2245	1861
ILMN_1665792	ITGA2	3673	161	165	265	230	289	228	729	547
ILMN_2367883	GEM	2669	206	384	185	294	202	252	988	1263
ILMN_1773742	DNAJB9	4189	678	1114	569	1164	500	877	2530	3583
ILMN_1787186	NOV	4856	391	316	414	433	375	351	932	1004
ILMN_1778064	FICD	11153	243	296	338	396	293	372	639	936
ILMN_1652287	NOG	9241	128	132	158	132	163	199	513	408
ILMN_1781560	ST3GAL6	10402	171	140	181	199	215	188	504	430
ILMN_1707312	NFIL3	4783	130	144	162	162	170	161	461	439
ILMN_1775501	IL1B	3553	196	383	309	517	392	595	1553	1151
ILMN_1678968	GEM	2669	111	128	110	128	124	125	211	370
ILMN_1683859	SLC7A1	6541	1132	1106	1192	1163	1233	1403	2680	3175
ILMN_1664362	SGIP1	84251	525	387	465	401	765	582	1693	1098
ILMN_1657451	SRPK2	6733	198	183	208	204	201	195	470	519
ILMN_1693014	CEBPB	1051	3933	3989	3020	3305	4105	3752	12632	11126
ILMN_2401873	DUSP10	11221	172	164	170	144	228	184	475	451
ILMN_1701361	C9orf150	286343	156	218	207	322	171	189	602	596
ILMN_1726456	SLC3A2	6520	894	1227	1102	1084	755	960	2729	3267
ILMN_1810533	SLC6A15	55117	132	141	148	170	160	172	394	362
ILMN_1687768	NCOA7	135112	1145	1241	1075	1402	1067	1254	3094	3135
ILMN_2318638	TGIF1	7050	1399	937	1096	899	1219	928	2911	2351
ILMN_1759513	RND3	390	2842	2578	1965	2664	2128	2199	7064	6430
ILMN_1804735	CBS	875	504	550	561	573	513	564	1065	1336
ILMN_1719695	NFKBIZ	64332	685	551	596	513	686	564	1380	1337
ILMN_1659544	STX3	6809	549	452	384	317	490	417	1036	1094
ILMN_1775380	SMOX	54498	197	278	265	317	244	298	549	650
ILMN_2119297	SAMD4A	23034	198	195	247	165	216	204	401	433
ILMN_1694731	CLCN7	1186	2746	2854	2545	3501	2918	3750	4711	6317
ILMN_2214678	MXD1	4084	233	270	279	308	253	264	494	592
ILMN_1673566	ADAMTS1	9510	417	253	315	204	299	290	1045	546
ILMN_2398107	ASNS	440	145	146	137	143	118	134	315	313
ILMN_1801313	SIAH2	6478	196	190	227	213	204	229	362	404
ILMN_1662618	SQSTM1	8878	3506	5240	4221	4645	3923	5415	7015	11142
ILMN_2094776	CCNL1	57018	675	643	657	671	786	689	1262	1365
ILMN_2266948	SLC38A1	81539	177	188	225	239	260	225	322	398
ILMN_1800512	HMOX1	3162	215	331	228	262	169	196	477	699
ILMN_1803143	SYVN1	84447	447	546	534	639	537	690	1138	1138
ILMN_1777060	CTH	1491	126	125	117	117	123	122	217	258
ILMN_1791728	SLC25A25	114789	624	731	758	773	744	858	1265	1466
ILMN_1768031	DEDD2	162989	836	683	717	825	786	739	1213	1364
ILMN_2188722	NA	NA	686	701	691	757	558	807	1155	1398
ILMN_1659682	GALK2	2585	182	209	190	241	212	232	271	414
ILMN_2137789	KLF4	9314	108	102	105	102	101	118	226	200
ILMN_1695745	DISP1	84976	220	251	220	341	237	347	423	492
ILMN_1705985	PIGA	5277	420	421	505	504	509	467	714	814
ILMN_2402600	GLIS3	169792	795	775	679	709	824	843	1738	1470
ILMN_1758633	CCDC130	81576	1802	2216	2127	2827	2093	2486	2950	4198
ILMN_1682197	NFXL1	152518	250	234	264	267	315	281	448	440
ILMN_1667561	IFRD1	3475	255	323	273	345	256	332	615	608
ILMN_1743204	DUSP8	1850	153	140	139	118	145	154	240	264
ILMN_2383349	STEAP3	55240	1102	950	971	957	1233	922	1688	1775

	Fold change over si-control and p-value<br>siSREBP1 EtOH	p-value	siSREBP1 4-OHT	p-value	siSREBP2 EtOH	p-value	siSREBP2 4-OHT	p-value	siSREBP1 +2 EtOH	p-value	siSREBP1 +2 4OHT	p-value	genename
ILMN_2054297	1.4113	0.1081	1.7689	0.0057	1.3962	0.1051	1.2405	0.2773	49.6437	0.0000	27.6623	0.0000	prostaglandin-endoperoxide synthase 2 (prostaglandin G/H synthase and cyclooxygenase)
ILMN_1744381	2.7124	0.0000	2.3572	0.0000	2.4839	0.0000	3.8956	0.0000	7.1821	0.0000	11.4179	0.0000	serpin peptidase inhibitor, clade E (nexin, plasminogen activator inhibitor type 1), member 1
ILMN_1676984	-1.3759	0.0053	-1.2451	0.0378	1.1356	0.2007	1.1102	0.2796	7.3355	0.0000	10.5062	0.0000	DNA-damage-inducible transcript 3
ILMN_1751607	-1.1202	0.4028	1.0296	0.8264	1.5676	0.0014	1.5924	0.0009	8.9778	0.0000	9.3092	0.0000	FBJ murine osteosarcoma viral oncogene homolog B
ILMN_1722718	1.8268	0.0000	1.6818	0.0000	2.5163	0.0000	2.1997	0.0000	13.3777	0.0000	8.4133	0.0000	bone morphogenetic protein 2
ILMN_1659936	-1.0581	0.5752	1.2611	0.0299	1.5758	0.0002	1.5644	0.0002	5.8993	0.0000	7.5415	0.0000	protein phosphatase 1, regulatory (inhibitor) subunit 15A
ILMN_1702487	3.0537	0.0000	2.8242	0.0000	3.2499	0.0000	2.4662	0.0000	9.8777	0.0000	7.1802	0.0000	serum/glucocorticoid regulated kinase 1
ILMN_1788107	1.2901	0.0404	1.6762	0.0004	1.6458	0.0003	2.5213	0.0000	5.4292	0.0000	6.9445	0.0000	interleukin 11
ILMN_1781285	1.0305	0.7245	1.0925	0.3280	1.0677	0.4471	1.2926	0.0084	4.6941	0.0000	6.7335	0.0000	dual specificity phosphatase 1
ILMN_1712112	1.2330	0.1277	1.4271	0.0136	-1.0213	0.8733	1.0971	0.5050	6.3418	0.0000	6.7112	0.0000	regulator of calcineurin 1
ILMN_1798210	1.9210	0.0001	1.3809	0.0280	2.6203	0.0000	2.2295	0.0000	6.9332	0.0000	6.4854	0.0000	E2F transcription factor 7
ILMN_2375879	1.8861	0.0038	2.6032	0.0000	1.9633	0.0024	2.7546	0.0000	5.2751	0.0000	6.4361	0.0000	vascular endothelial growth factor A
ILMN_1805192	1.7990	0.0000	1.6509	0.0000	1.3766	0.0003	1.5084	0.0000	6.3837	0.0000	6.2318	0.0000	inositol 1,4,5-triphosphate receptor interacting protein
ILMN_2374865	-1.4638	0.0097	-1.9706	0.0000	-1.3048	0.0542	-1.9285	0.0000	8.0930	0.0000	6.2290	0.0000	activating transcription factor 3
ILMN_1787815	1.0855	0.5818	1.0815	0.5847	1.0889	0.5619	-1.0937	0.5329	4.6113	0.0000	6.1051	0.0000	tribbles homolog 3 (Drosophila)
ILMN_1708041	1.0408	0.8140	1.3878	0.0690	1.2786	0.1604	1.3657	0.0759	3.6672	0.0000	6.0071	0.0000	pleckstrin homology domain containing, family F (with FYVE domain) member 1
ILMN_1738742	2.8753	0.0000	1.9725	0.0000	2.3799	0.0000	1.2330	0.0305	10.4057	0.0000	5.4962	0.0000	plasminogen activator, tissue
ILMN_2367239	1.0620	0.4920	1.1168	0.2241	-1.1281	0.1811	-1.0253	0.7833	5.6863	0.0000	5.3384	0.0000	regulator of calcineurin 1
ILMN_1796417	-1.0963	0.3505	-1.1496	0.1583	-1.1614	0.1370	-1.3214	0.0090	4.5392	0.0000	5.2980	0.0000	asparagine synthetase
ILMN_1811767	1.0520	0.7015	1.0665	0.6232	-1.0345	0.8096	1.0548	0.6877	3.0556	0.0000	5.0859	0.0000	inhibin, beta E
ILMN_1806023	1.4733	0.0000	1.3794	0.0003	2.0006	0.0000	2.0138	0.0000	4.2335	0.0000	5.0839	0.0000	jun oncogene
ILMN_2305112	-1.3317	0.0665	-1.0165	0.9086	-1.2926	0.0955	1.2130	0.2116	3.2186	0.0000	4.9112	0.0000	cystathionase (cystathionine gamma-lyase)
ILMN_2374159	-1.0096	0.9249	1.1621	0.1313	-1.2491	0.0315	-1.1161	0.2730	3.4795	0.0000	4.7942	0.0000	homocysteine-inducible, endoplasmic reticulum stress-inducible, ubiquitin-like domain member 1
ILMN_2405521	1.6794	0.0000	1.4334	0.0002	1.5248	0.0000	1.6501	0.0000	4.2218	0.0000	4.7511	0.0000	methylenetetrahydrofolate dehydrogenase (NADP+ dependent) 2, methenyltetrahydrofolate cyclohydrolase
ILMN_1699651	2.0166	0.0000	2.2885	0.0000	1.3970	0.0109	1.7053	0.0001	6.6645	0.0000	4.6346	0.0000	interleukin 6 (interferon, beta 2)
ILMN_1809931	-1.0417	0.4434	-1.0489	0.3879	1.2132	0.0015	1.0280	0.6033	4.0681	0.0000	4.2983	0.0000	N-myc downstream regulated 1
ILMN_1731714	1.3994	0.0095	1.3483	0.0181	1.6523	0.0003	1.4545	0.0039	4.5580	0.0000	4.2492	0.0000	cAMP responsive element binding protein 5
ILMN_1720158	-1.1754	0.1335	1.4269	0.0022	1.0487	0.6469	1.1996	0.0904	2.8244	0.0000	4.0221	0.0000	v-ets erythroblastosis virus E26 oncogene homolog 2 (avian)
ILMN_1782110	1.0284	0.7176	1.2794	0.0050	1.2480	0.0057	1.4115	0.0002	2.7008	0.0000	3.7661	0.0000	zinc finger protein 295
ILMN_1658483	1.1740	0.1777	1.5299	0.0003	1.1448	0.2549	-1.0263	0.8065	3.3973	0.0000	3.7509	0.0000	interleukin 1, alpha
ILMN_1684158	-1.1776	0.1535	1.2416	0.0586	1.0801	0.4864	1.2397	0.0588	2.2892	0.0000	3.7104	0.0000	glutamic pyruvate transaminase (alanine aminotransferase) 2
ILMN_1679041	1.5277	0.0015	1.2156	0.0906	1.0553	0.6576	-1.3425	0.0178	4.2066	0.0000	3.6905	0.0000	solute carrier family 3 (activators of dibasic and neutral amino acid transport), member 2
ILMN_1745964	1.7354	0.0001	1.5782	0.0003	1.5519	0.0009	1.3837	0.0055	5.5860	0.0000	3.6168	0.0000	interleukin-1 receptor-associated kinase 2
ILMN_1716766	1.1888	0.0264	1.1180	0.1399	1.2357	0.0082	1.1229	0.1258	3.6450	0.0000	3.4640	0.0000	CCAAT/enhancer binding protein (C/EBP), gamma
ILMN_1674706	1.4608	0.0054	1.2772	0.0572	1.3151	0.0381	1.4579	0.0053	4.1773	0.0000	3.4155	0.0000	methylenetetrahydrofolate dehydrogenase (NADP+ dependent) 2, methenyltetrahydrofolate cyclohydrolase
ILMN_1682717	1.4212	0.0003	1.1982	0.0333	1.6785	0.0000	1.2913	0.0042	5.5548	0.0000	3.3615	0.0000	immediate early response 3
ILMN_1739222	1.5641	0.0001	1.5347	0.0003	1.4118	0.0015	1.5965	0.0001	3.1499	0.0000	3.3431	0.0000	ets variant 5
ILMN_1665792	1.6441	0.0000	1.3979	0.0000	1.7953	0.0000	1.3853	0.0000	4.5315	0.0000	3.3230	0.0000	integrin, alpha 2 (CD49B, alpha 2 subunit of VLA-2 receptor)
ILMN_2367883	-1.1091	0.2531	-1.3056	0.0021	-1.0172	0.8486	-1.5254	0.0000	4.8080	0.0000	3.2878	0.0000	GTP binding protein overexpressed in skeletal muscle
ILMN_1773742	-1.1922	0.1708	1.0445	0.7091	-1.3567	0.0242	-1.2700	0.0548	3.7311	0.0000	3.2164	0.0000	DnaJ (Hsp40) homolog, subfamily B, member 9
ILMN_1787186	1.0588	0.4599	1.3691	0.0006	-1.0425	0.5909	1.1109	0.1983	2.3855	0.0000	3.1739	0.0000	nephroblastoma overexpressed gene
ILMN_1778064	1.3917	0.0042	1.3380	0.0073	1.2063	0.0867	1.2565	0.0305	2.6303	0.0000	3.1618	0.0000	FIC domain containing
ILMN_1652287	1.2389	0.0767	-1.0018	0.9786	1.2806	0.0409	1.5045	0.0011	4.0184	0.0000	3.0885	0.0000	noggin
ILMN_1781560	1.0602	0.3036	1.4158	0.0000	1.2620	0.0004	1.3405	0.0001	2.9502	0.0000	3.0692	0.0000	ST3 beta-galactoside alpha-2,3-sialyltransferase 6
ILMN_1707312	1.2480	0.0071	1.1294	0.1142	1.3102	0.0015	1.1172	0.1472	3.5619	0.0000	3.0501	0.0000	nuclear factor, interleukin 3 regulated
ILMN_1775501	1.5801	0.0007	1.3481	0.0064	2.0063	0.0000	1.5517	0.0002	7.9445	0.0000	3.0029	0.0000	interleukin 1, beta
ILMN_1678968	-1.0119	0.9227	-1.0029	0.9810	1.1106	0.3654	-1.0266	0.8087	1.8931	0.0000	2.8789	0.0000	GTP binding protein overexpressed in skeletal muscle
ILMN_1683859	1.0531	0.6357	1.0516	0.6445	1.0891	0.4356	1.2685	0.0377	2.3670	0.0000	2.8702	0.0000	solute carrier family 7 (cationic amino acid transporter, y+ system), member 1
ILMN_1664362	-1.1274	0.2195	1.0357	0.7188	1.4579	0.0005	1.5048	0.0004	3.2275	0.0000	2.8401	0.0000	SH3-domain GRB2-like (endophilin) interacting protein 1
ILMN_1657451	1.0509	0.3523	1.1135	0.0551	1.0155	0.7743	1.0603	0.2753	2.3715	0.0000	2.8294	0.0000	SFRS protein kinase 2
ILMN_1693014	-1.3024	0.0043	-1.2070	0.0322	1.0438	0.6028	-1.0631	0.4613	3.2118	0.0000	2.7894	0.0000	CCAAT/enhancer binding protein (C/EBP), beta
ILMN_2401873	-1.0161	0.8882	-1.1356	0.2714	1.3241	0.0125	1.1259	0.2636	2.7573	0.0000	2.7566	0.0000	dual specificity phosphatase 10
ILMN_1701361	1.3264	0.0049	1.4788	0.0001	1.0945	0.3400	-1.1508	0.1161	3.8480	0.0000	2.7381	0.0000	chromosome 9 open reading frame 150
ILMN_1726456	1.2317	0.0165	-1.1320	0.1284	-1.1853	0.0477	-1.2786	0.0054	3.0508	0.0000	2.6627	0.0000	solute carrier family 3 (activators of dibasic and neutral amino acid transport), member 2
ILMN_1810533	1.1186	0.2892	1.2031	0.0732	1.2116	0.0721	1.2162	0.0593	2.9879	0.0000	2.5631	0.0000	solute carrier family 6 (neutral amino acid transporter), member 15
ILMN_1687768	-1.0653	0.5199	1.1296	0.2182	-1.0737	0.4684	1.0104	0.9121	2.7016	0.0000	2.5255	0.0000	nuclear receptor coactivator 7
ILMN_2318638	-1.2765	0.0243	-1.0423	0.6964	-1.1479	0.1814	-1.0104	0.9218	2.0807	0.0000	2.5079	0.0000	TGFB-induced factor homeobox 1
ILMN_1759513	-1.4466	0.0005	1.0332	0.7126	-1.3358	0.0037	-1.1723	0.0863	2.4852	0.0000	2.4938	0.0000	Rho family GTPase 3
ILMN_1804735	1.1122	0.2433	1.0409	0.6518	1.0184	0.8452	1.0257	0.7707	2.1130	0.0000	2.4281	0.0000	cystathionine-beta-synthase
ILMN_1719695	-1.1498	0.2022	-1.0740	0.5205	1.0008	0.9818	1.0227	0.8225	2.0133	0.0000	2.4260	0.0000	nuclear factor of kappa light polypeptide gene enhancer in B-cells inhibitor, zeta
ILMN_1659544	-1.4274	0.0001	-1.4264	0.0001	-1.1199	0.1275	-1.0823	0.2996	1.8891	0.0000	2.4206	0.0000	syntaxin 3
ILMN_1775380	1.3452	0.0088	1.1406	0.1795	1.2383	0.0519	1.0717	0.4803	2.7836	0.0000	2.3395	0.0000	spermine oxidase
ILMN_2119297	1.2503	0.0491	-1.1774	0.1740	1.0912	0.4349	1.0492	0.6594	2.0270	0.0000	2.2237	0.0000	sterile alpha motif domain containing 4A
ILMN_1694731	-1.0789	0.6115	1.2267	0.1770	1.0625	0.6824	1.3139	0.0753	1.7155	0.0014	2.2130	0.0000	chloride channel 7
ILMN_2214678	1.1967	0.0607	1.1419	0.1356	1.0858	0.3780	-1.0208	0.8177	2.1210	0.0000	2.1965	0.0000	MAX dimerization protein 1
ILMN_1673566	-1.3231	0.0054	-1.2409	0.0421	-1.3938	0.0014	1.1444	0.1815	2.5058	0.0000	2.1575	0.0000	ADAM metallopeptidase with thrombospondin type 1 motif, 1
ILMN_2398107	-1.0554	0.6586	-1.0184	0.8902	-1.2246	0.1174	-1.0898	0.4769	2.1735	0.0000	2.1477	0.0000	asparagine synthetase
ILMN_1801313	1.1560	0.0955	1.1185	0.1964	1.0387	0.6520	1.2037	0.0359	1.8448	0.0000	2.1272	0.0000	seven in absentia homolog 2 (Drosophila)
ILMN_1662618	1.2038	0.1169	-1.1281	0.2941	1.1189	0.3334	1.0335	0.7723	2.0007	0.0000	2.1263	0.0000	sequestosome 1
ILMN_2094776	-1.0267	0.6615	1.0443	0.4803	1.1648	0.0174	1.0714	0.2626	1.8709	0.0000	2.1238	0.0000	cyclin L1
ILMN_2266948	1.2713	0.0569	1.2688	0.0523	1.4659	0.0038	1.1957	0.1458	1.8141	0.0000	2.1163	0.0000	solute carrier family 38, member 1
ILMN_1800512	1.0594	0.6364	-1.2616	0.0493	-1.2743	0.0699	-1.6918	0.0002	2.2183	0.0000	2.1108	0.0000	heme oxygenase (decycling) 1
ILMN_1803143	1.1962	0.1131	1.1695	0.1548	1.2016	0.1027	1.2627	0.0382	2.5476	0.0000	2.0840	0.0000	synovial apoptosis inhibitor 1, synoviolin
ILMN_1777060	-1.0785	0.4083	-1.0716	0.4535	-1.0260	0.7749	-1.0265	0.7829	1.7179	0.0000	2.0650	0.0000	cystathionase (cystathionine gamma-lyase)
ILMN_1791728	1.2138	0.0288	1.0571	0.5052	1.1918	0.0455	1.1737	0.0619	2.0263	0.0000	2.0060	0.0000	solute carrier family 25 (mitochondrial carrier; phosphate carrier), member 25
ILMN_1768031	-1.1655	0.1298	1.2079	0.0681	-1.0638	0.5303	1.0816	0.4295	1.4513	0.0008	1.9972	0.0000	death effector domain containing 2
ILMN_2188722	1.0064	0.9617	1.0802	0.4793	-1.2308	0.0714	1.1508	0.2056	1.6826	0.0001	1.9937	0.0000	NA
ILMN_1659682	1.0453	0.5375	1.1534	0.0397	1.1646	0.0365	1.1066	0.1379	1.4884	0.0000	1.9768	0.0000	galactokinase 2
ILMN_2137789	-1.0312	0.7743	-1.0011	0.9985	-1.0657	0.5661	1.1615	0.1751	2.0932	0.0000	1.9654	0.0000	Kruppel-like factor 4 (gut)
ILMN_1695745	1.0009	0.9957	1.3559	0.0178	1.0756	0.5713	1.3810	0.0121	1.9211	0.0000	1.9575	0.0000	dispatched homolog 1 (Drosophila)
ILMN_1705985	1.2035	0.0890	1.1965	0.0985	1.2138	0.0762	1.1107	0.3246	1.7025	0.0000	1.9338	0.0000	phosphatidylinositol glycan anchor biosynthesis, class A
ILMN_2402600	-1.1704	0.1431	-1.0932	0.4037	1.0369	0.7262	1.0881	0.4124	2.1857	0.0000	1.8972	0.0000	GLIS family zinc finger 3
ILMN_1758633	1.1805	0.1085	1.2762	0.0213	1.1615	0.1459	1.1221	0.2505	1.6371	0.0001	1.8948	0.0000	coiled-coil domain containing 130
ILMN_1682197	1.0589	0.5457	1.1426	0.1708	1.2624	0.0185	1.2023	0.0612	1.7953	0.0000	1.8809	0.0000	nuclear transcription factor, X-box binding-like 1
ILMN_1667561	1.0729	0.4297	1.0668	0.4388	1.0066	0.9375	1.0266	0.7504	2.4141	0.0000	1.8795	0.0000	interferon-related developmental regulator 1
ILMN_1743204	-1.0946	0.3377	-1.1926	0.0856	-1.0552	0.5613	1.0987	0.3033	1.5728	0.0000	1.8791	0.0000	dual specificity phosphatase 8
ILMN_2383349	-1.1344	0.0666	1.0075	0.9093	1.1187	0.0977	-1.0306	0.6513	1.5323	0.0000	1.8684	0.0000	STEAP family member 3

ILMN_1686152	GGA2	23062	463	451	424	518	488	517	585	840	-1.0910	0.2112	1.1500	0.0485	1.0539	0.4372	1.1476	0.0523	1.2629	0.0019	1.8633	0.0000	golgi-associated, gamma adaptin ear containing, ARF binding protein 2
ILMN_1740426	RASD1	51655	108	107	106	101	113	113	158	199	-1.0210	0.7528	-1.0568	0.4212	1.0480	0.4862	1.0508	0.4746	1.4548	0.0000	1.8552	0.0000	RAS, dexamethasone-induced 1
ILMN_1807719	CTNS	1497	384	456	342	438	384	453	587	835	-1.1221	0.3819	-1.0429	0.7255	-1.0004	0.9984	-1.0067	0.9799	1.5291	0.0022	1.8298	0.0000	cystinosis, nephropathic
ILMN_1703477	ARHGEF2	9181	1639	1869	1568	1529	1403	1750	3479	3406	-1.0455	0.6330	-1.2224	0.0449	-1.1682	0.1128	-1.0681	0.4883	2.1221	0.0000	1.8220	0.0000	Rho/Rac guanine nucleotide exchange factor (GEF) 2
ILMN_1798867	CAMTA2	23125	118	134	117	117	108	129	188	243	-1.0062	0.9189	-1.1466	0.0612	-1.0905	0.2452	-1.0414	0.5519	1.5903	0.0000	1.8137	0.0000	calmodulin binding transcription activator 2
ILMN_2117171	LMO4	8543	1472	952	1766	1541	1638	1415	2533	1724	1.1997	0.2676	1.6196	0.0078	1.1125	0.5125	1.4870	0.0247	1.7207	0.0026	1.8122	0.0015	LIM domain only 4
ILMN_1766010	YARS	8565	2625	2738	2719	3176	2568	3105	4103	4947	1.0359	0.5720	1.1601	0.0254	-1.0222	0.7248	1.1340	0.0534	1.5631	0.0000	1.8070	0.0000	tyrosyl-tRNA synthetase
ILMN_1657870	ABL2	27	162	134	155	130	152	120	233	238	-1.0393	0.6585	-1.0236	0.8061	-1.0640	0.4660	-1.1148	0.2592	1.4426	0.0002	1.7803	0.0000	v-abl Abelson murine leukemia viral oncogene homolog 2 (arg, Abelson-related gene)
ILMN_1753111	NAMPT	10135	224	271	248	335	262	266	337	477	1.1098	0.3674	1.2371	0.0548	1.1685	0.1829	-1.0191	0.8750	1.5067	0.0012	1.7582	0.0000	nicotinamide phosphoribosyltransferase
ILMN_2084836	CORO2B	10391	227	177	296	240	243	218	398	312	1.3037	0.0363	1.3524	0.0275	1.0684	0.6059	1.2279	0.1208	1.7516	0.0001	1.7572	0.0002	coronin, actin binding protein, 2B
ILMN_1760160	STX1A	6804	556	469	600	375	541	501	967	823	1.0780	0.5239	-1.2490	0.0925	-1.0285	0.8122	1.0698	0.5441	1.7370	0.0001	1.7564	0.0001	syntaxin 1A (brain)
ILMN_1727200	SLCO4A1	28231	139	141	159	170	157	151	203	247	1.1464	0.0556	1.2023	0.0113	1.1295	0.0858	1.0686	0.3175	1.4615	0.0000	1.7492	0.0000	solute carrier organic anion transporter family, member 4A1
ILMN_2400322	DYRK3	8444	274	288	289	269	304	294	439	501	1.0530	0.4772	-1.0684	0.3737	1.1107	0.1533	1.0217	0.7699	1.6002	0.0000	1.7428	0.0000	dual-specificity tyrosine-(Y)-phosphorylation regulated kinase 3
ILMN_1684726	C2orf27A	29798	138	125	142	123	158	147	276	218	1.0304	0.6295	-1.0188	0.7722	1.1487	0.0338	1.1760	0.0182	1.9995	0.0000	1.7416	0.0000	chromosome 2 open reading frame 27A
ILMN_2108938	FNBP4	23360	1539	1332	1393	1284	1647	1604	2109	2316	-1.1049	0.2495	-1.0368	0.6731	1.0700	0.4314	1.2041	0.0399	1.3700	0.0012	1.7391	0.0000	formin binding protein 4
ILMN_2412761	MAFG	4097	269	293	243	269	248	315	471	506	-1.1093	0.2796	-1.0907	0.3482	-1.0864	0.3846	1.0757	0.4137	1.7484	0.0000	1.7260	0.0000	v-maf musculoaponeurotic fibrosarcoma oncogene homolog G (avian)
ILMN_1662839	PLEKHA1	59338	908	713	887	938	829	986	1527	1222	-1.0239	0.8603	1.3154	0.0447	-1.0953	0.4802	1.3825	0.0197	1.6814	0.0004	1.7126	0.0004	pleckstrin homology domain containing, family A (phosphoinositide binding specific) member 1
ILMN_1743763	TJAP1	93643	868	1123	1103	1496	1221	1492	1562	1904	1.2705	0.1031	1.3321	0.0503	1.4064	0.0245	1.3287	0.0518	1.7991	0.0004	1.6959	0.0010	tight junction associated protein 1 (peripheral)
ILMN_1728677	CREB5	9586	103	110	114	112	117	116	170	186	1.1118	0.1268	1.0227	0.7350	1.1380	0.0652	1.0522	0.4499	1.6558	0.0000	1.6938	0.0000	cAMP responsive element binding protein 5
ILMN_1779706	TP53BP2	7159	656	656	617	597	721	767	1081	1109	-1.0619	0.4530	-1.0990	0.2468	1.0989	0.2410	1.1692	0.0591	1.6491	0.0000	1.6905	0.0000	tumor protein p53 binding protein, 2
ILMN_1811997	RNF115	27246	2520	2202	2539	2259	2783	2446	3284	3704	1.0074	0.9146	1.0262	0.7111	1.1044	0.1613	1.1108	0.1408	1.3029	0.0009	1.6821	0.0000	ring finger protein 115
ILMN_1702683	SLC33A1	9197	191	184	195	213	165	214	291	308	1.0203	0.8027	1.1584	0.1303	-1.1579	0.1411	1.1615	0.1258	1.5235	0.0001	1.6748	0.0000	solute carrier family 33 (acetyl-CoA transporter), member 1
ILMN_1678904	ENO3	2027	225	217	198	263	213	242	315	362	-1.1392	0.2827	1.2109	0.1138	-1.0593	0.6174	1.1152	0.3446	1.3950	0.0082	1.6705	0.0002	enolase 3 (beta, muscle)
ILMN_1766275	PIK3CD	5293	250	207	232	221	250	224	403	345	-1.0817	0.2671	1.0679	0.3805	-1.0030	0.9623	1.0819	0.2963	1.6111	0.0000	1.6690	0.0000	phosphoinositide-3-kinase, catalytic, delta polypeptide
ILMN_1721138	GRPEL2	134266	753	697	744	795	765	783	1109	1159	-1.0121	0.9114	1.1408	0.2409	1.0155	0.8895	1.1239	0.2964	1.4718	0.0016	1.6628	0.0001	GrpE-like 2, mitochondrial (E. coli)
ILMN_2175465	RSL24D1	51187	3490	2803	3078	3232	3186	3166	4792	4631	-1.1340	0.0739	1.1531	0.0458	-1.0954	0.1866	1.1295	0.0832	1.3728	0.0001	1.6524	0.0000	ribosomal L24 domain containing 1
ILMN_2339955	NR4A2	4929	111	111	108	108	102	109	216	184	-1.0306	0.7642	-1.0277	0.7980	-1.0960	0.3943	-1.0185	0.8572	1.9347	0.0000	1.6504	0.0000	nuclear receptor subfamily 4, group A, member 2
ILMN_1741003	ANXA5	308	14630	13536	18836	17611	17902	16457	19694	22126	1.2875	0.0061	1.3011	0.0046	1.2236	0.0234	1.2158	0.0277	1.3461	0.0018	1.6347	0.0000	annexin A5
ILMN_2415157	ARID5A	10865	133	128	131	115	134	125	197	210	-1.0148	0.8180	-1.1187	0.0952	1.0102	0.8659	-1.0244	0.6876	1.4824	0.0000	1.6339	0.0000	AT rich interactive domain 5A (MRF1-like)
ILMN_1671791	PCK2	5106	587	580	335	254	416	313	984	947	-1.7500	0.0000	-2.2795	0.0000	-1.4099	0.0005	-1.8522	0.0000	1.6761	0.0000	1.6333	0.0000	phosphoenolpyruvate carboxykinase 2 (mitochondrial)
ILMN_1815121	PLAGL1	5325	225	186	208	185	218	217	263	304	-1.0791	0.2585	-1.0091	0.9040	-1.0286	0.6673	1.1656	0.0321	1.1698	0.0200	1.6320	0.0000	pleiomorphic adenoma gene-like 1
ILMN_1777765	C12orf10	60314	2102	2879	2273	3880	2589	3270	2999	4668	1.0815	0.4192	1.3479	0.0047	1.2318	0.0401	1.1359	0.1878	1.4264	0.0013	1.6215	0.0000	chromosome 12 open reading frame 10
ILMN_1743137	BAT2L2	23215	1430	1330	1379	1475	1347	1552	1859	2147	-1.0373	0.5990	1.1090	0.1537	-1.0616	0.3970	1.1667	0.0390	1.2998	0.0012	1.6141	0.0000	HLA-B associated transcript 2-like 2
ILMN_1707748	PIM3	415116	181	215	187	206	207	236	325	345	1.0374	0.5515	-1.0421	0.4833	1.1463	0.0343	1.0996	0.1098	1.7978	0.0000	1.6089	0.0000	pim-3 oncogene
ILMN_1684653	GPR115	221393	106	99	102	104	103	110	130	160	-1.0406	0.4806	1.0513	0.3941	-1.0229	0.6779	1.1119	0.0751	1.2269	0.0012	1.6065	0.0000	G protein-coupled receptor 115
ILMN_1744239	FEM1B	10116	268	276	306	313	329	328	334	442	1.1407	0.0521	1.1336	0.0672	1.2256	0.0050	1.1879	0.0147	1.2451	0.0028	1.6018	0.0000	fem-1 homolog b (C. elegans)
ILMN_1666206	GSDMB	55876	173	156	173	152	176	145	229	249	-1.0032	0.9348	-1.0310	0.6584	1.0129	0.8706	-1.0814	0.2570	1.3216	0.0002	1.5958	0.0000	gasdermin B
ILMN_1796642	NCF2	4688	132	134	152	148	154	146	180	214	1.1477	0.0695	1.1002	0.1910	1.1672	0.0428	1.0849	0.2743	1.3600	0.0003	1.5928	0.0000	neutrophil cytosolic factor 2
ILMN_1703688	RASA2	5922	142	134	176	155	174	168	228	214	1.2369	0.0089	1.1498	0.0820	1.2237	0.0119	1.2473	0.0079	1.6051	0.0000	1.5927	0.0000	RAS p21 protein activator 2
ILMN_1797594	NFAT5	10725	282	270	293	302	237	281	375	429	1.0409	0.7122	1.1183	0.2737	-1.1903	0.1048	1.0418	0.6931	1.3324	0.0081	1.5899	0.0001	nuclear factor of activated T-cells 5, tonicity-responsive
ILMN_2226917	KIAA0247	9766	245	300	282	329	261	271	588	476	1.1499	0.1278	1.0963	0.2884	1.0635	0.4961	-1.1065	0.2613	2.3988	0.0000	1.5872	0.0000	KIAA0247
ILMN_1698968	ASXL2	55252	493	486	545	573	506	578	771	771	1.1035	0.1776	1.1780	0.0304	1.0259	0.7245	1.1889	0.0228	1.5631	0.0000	1.5867	0.0000	additional sex combs like 2 (Drosophila)
ILMN_1664099	GLIS3	169792	179	167	166	167	174	200	272	264	-1.0754	0.4373	1.0011	0.9531	-1.0276	0.7663	1.1970	0.0544	1.5207	0.0001	1.5849	0.0000	GLIS family zinc finger 3
ILMN_1770373	TMEM39A	55254	233	296	240	291	254	306	360	468	1.0283	0.6901	-1.0168	0.7948	1.0864	0.2347	1.0349	0.6031	1.5421	0.0000	1.5814	0.0000	transmembrane protein 39A
ILMN_1795678	POLR3C	10623	1656	1536	2027	1765	1821	1779	2362	2420	1.2245	0.0037	1.1491	0.0361	1.0997	0.1388	1.1581	0.0279	1.4268	0.0000	1.5752	0.0000	polymerase (RNA) III (DNA directed) polypeptide C (62kD)
ILMN_1718266	DGKI	9162	124	125	131	154	125	124	180	196	1.0569	0.5624	1.2334	0.0283	1.0116	0.9130	-1.0013	0.9926	1.4558	0.0003	1.5738	0.0000	diacylglycerol kinase, iota
ILMN_1743643	ZXDC	79364	159	183	174	196	204	205	225	287	1.0955	0.3082	1.0719	0.4254	1.2834	0.0092	1.1168	0.2052	1.4154	0.0006	1.5678	0.0000	ZXD family zinc finger C
ILMN_1697153	ZDHHC17	23390	789	692	820	814	1000	858	1412	1081	1.0397	0.6597	1.1769	0.0779	1.2680	0.0121	1.2400	0.0227	1.7893	0.0000	1.5631	0.0000	zinc finger, DHHC-type containing 17
ILMN_1660663	DYRK1A	1859	328	338	371	386	401	365	416	527	1.1310	0.0678	1.1411	0.0484	1.2206	0.0050	1.0795	0.2424	1.2666	0.0013	1.5592	0.0000	dual-specificity tyrosine-(Y)-phosphorylation regulated kinase 1A
ILMN_1755303	ZNF217	7764	444	419	467	548	495	486	760	646	1.0529	0.5928	1.3085	0.0104	1.1140	0.2746	1.1593	0.1403	1.7125	0.0000	1.5411	0.0002	zinc finger protein 217
ILMN_1729217	FAM131A	131408	250	276	290	340	305	337	379	424	1.1595	0.0715	1.2326	0.0124	1.2164	0.0206	1.2213	0.0152	1.5118	0.0000	1.5370	0.0000	family with sequence similarity 131, member A
ILMN_1653871	NAMPT	10135	204	218	206	232	210	212	278	335	1.0131	0.8549	1.0646	0.4017	1.0296	0.7012	-1.0287	0.6919	1.3622	0.0004	1.5366	0.0000	nicotinamide phosphoribosyltransferase
ILMN_1781386	WIPI1	55062	819	914	939	1089	782	919	1198	1403	1.1467	0.0705	1.1911	0.0225	-1.0479	0.5209	1.0056	0.9312	1.4629	0.0000	1.5354	0.0000	WD repeat domain, phosphoinositide interacting 1
ILMN_1758311	NET1	10276	1062	822	1211	923	1241	998	1946	1256	1.1402	0.0651	1.1229	0.1097	1.1681	0.0311	1.2143	0.0107	1.8327	0.0000	1.5285	0.0000	neuroepithelial cell transforming 1
ILMN_1764361	DUSP16	80824	142	150	137	140	160	148	217	229	-1.0400	0.5577	-1.0728	0.2737	1.1260	0.0646	-1.0137	0.8298	1.5264	0.0000	1.5263	0.0000	dual specificity phosphatase 16
ILMN_1659926	ST7L	54879	143	178	156	215	168	193	231	272	1.0936	0.1852	1.2046	0.0062	1.1746	0.0251	1.0850	0.2023	1.6200	0.0000	1.5252	0.0000	suppression of tumorigenicity 7 like
ILMN_2209221	DMRT1	1761	102	99	103	98	116	120	187	151	1.0141	0.8917	-1.0155	0.8857	1.1377	0.1986	1.2074	0.0688	1.8355	0.0000	1.5236	0.0002	doublesex and mab-3 related transcription factor 1
ILMN_2412024	JMJD6	23210	180	214	212	245	214	252	239	326	1.1786	0.0431	1.1476	0.0696	1.1850	0.0380	1.1798	0.0326	1.3241	0.0012	1.5234	0.0000	jumonji domain containing 6
ILMN_1654099	LRIG2	9860	160	162	172	184	175	179	225	246	1.0749	0.2328	1.1385	0.0377	1.0987	0.1217	1.1071	0.0992	1.4091	0.0000	1.5202	0.0000	leucine-rich repeats and immunoglobulin-like domains 2
ILMN_1691432	PRDM4	11108	535	583	586	715	546	580	717	885	1.0944	0.3008	1.2273	0.0232	1.0198	0.8248	-1.0054	0.9597	1.3387	0.0024	1.5189	0.0001	PR domain containing 4
ILMN_1702211	TGIF1	7050	118	116	119	115	121	117	194	176	1.0140	0.8561	-1.0138	0.8621	1.0296	0.7026	1.0058	0.9399	1.6467	0.0000	1.5132	0.0000	TGFB-induced factor homeobox 1
ILMN_2314080	CTAGE5	4253	113	113	124	126	129	122	179	169	1.0931	0.1801	1.1179	0.0922	1.1369	0.0564	1.0838	0.2199	1.5808	0.0000	1.5013	0.0000	CTAGE family, member 5
ILMN_2298261	NA	NA	109	114	123	133	124	138	156	172	1.1308	0.2055	1.1660	0.1265	1.1406	0.1817	1.2087	0.0586	1.4341	0.0007	1.5008	0.0002	NA
ILMN_2073732	CBLL1	79872	624	625	644	765	748	822	958	936	1.0324	0.7428	1.2232	0.0493	1.1996	0.0733	1.3147	0.0096	1.5357	0.0002	1.4968	0.0004	Cas-Br-M (murine) ecotropic retroviral transforming sequence-like 1
ILMN_1715969	SLC25A37	51312	634	592	779	719	639	536	843	885	1.2292	0.0326	1.2140	0.0455	1.0081	0.9336	-1.1047	0.2966	1.3303	0.0046	1.4952	0.0002	solute carrier family 25, member 37
ILMN_2117716	SFRS17A	8227	1317	1394	1345	1264	1269	1430	2052	2078	1.0211	0.7986	-1.1036	0.2364	-1.0383	0.6459	1.0254	0.7601	1.5576	0.0000	1.4902	0.0001	splicing factor, arginine/serine-rich 17A
ILMN_2358914	SLC35C2	51006	414	408	448	493	411	435	541	606	1.0834	0.2444	1.2074	0.0099	-1.0074	0.9070	1.0657	0.3513	1.3077	0.0006	1.4825	0.0000	solute carrier family 35, member C2
ILMN_1815656	SERINC3	10955	2728	3051	3023	3583	3246	3479	3858	4521	1.1081	0.2403	1.1743	0.0718	1.1899	0.0537	1.1402	0.1354	1.4144	0.0006	1.4817	0.0002	serine incorporator 3
ILMN_1743583	MADD	8567	166	202	180	254	216	239	236	298	1.0859	0.4509	1.2583	0.0214	1.3022	0.0150	1.1837	0.0793	1.4190	0.0018	1.4797	0.0003	MAP-kinase activating death domain
ILMN_1783695	EPRS	2058	3401	3994	3496	4686	3585	4554	5002	5892	1.0279	0.6438	1.1734	0.0129	1.0541	0.3808	1.1402	0.0364	1.4709	0.0000	1.4751	0.0000	glutamyl-prolyl-tRNA synthetase
ILMN_1790534	MAP2K3	5606	412	472	407	494	503	466	647	695	-1.0132	0.8680	1.0467	0.5779	1.2196	0.0215	-1.0119	0.8763	1.5683	0.0000	1.4732	0.0001	mitogen-activated protein kinase kinase 3
ILMN_1782439	CNN3	1266	10035	8633	11314	11095	11590	10675	14987	12713	1.1275	0.1367	1.2852	0.0042	1.1550	0.0775	1.2365	0.0128	1.4935	0.0000	1.4725	0.0001	calponin 3, acidic
ILMN_2267535	NET1	10276	166	154	208	197	211	169	248	227	1.2562	0.0253	1.2736	0.0191	1.2733	0.0187	1.0934	0.3805	1.4974	0.0003	1.4715	0.0005	neuroepithelial cell transforming 1
ILMN_1654685	MCTP1	79772	352	437	305	498	341	482	473	643	-1.1550	0.0946	1.1380	0.1037	-1.0303	0.7161	1.1022	0.2121	1.3442	0.0012	1.4711	0.0000	multiple C2 domains, transmembrane 1
ILMN_1792276	CCDC149	91050	126	123	116	116	120	119	144	180	-1.0828	0.1650	-1.0603	0.3034	-1.0465	0.4259	-1.0290	0.6245	1.1397	0.0220	1.4672	0.0000	coiled-coil domain containing 149
ILMN_1713759	UBE2J1	51465	1216	1061	1099	1137	1300	1211	1539	1556	-1.1061	0.1721	1.0720	0.3424	1.0689	0.3564	1.1419	0.0784	1.2658	0.0033	1.4670	0.0000	ubiquitin-conjugating enzyme E2, J1 (UBC6 homolog, yeast)
ILMN_2361862	VLDLR	7436	112	117	120	132	125	124	142	171	1.0685	0.2290	1.1290	0.0299	1.1137	0.0559	1.0583	0.2976	1.2726	0.0001	1.4605	0.0000	very low density lipoprotein receptor
ILMN_1729167	EFTUD1	79631	319	260	304	250	318	262	400	380	-1.0467	0.4541	-1.0398	0.5441	-1.0018	0.9788	1.0055	0.9397	1.2560	0.0009	1.4601	0.0000	elongation factor Tu GTP binding domain containing 1
ILMN_1665964	GAB2	9846	207	174	199	170	218	195	307	252	-1.0403	0.5850	-1.0210	0.7739	1.0529	0.4521	1.1238	0.1145	1.4843	0.0000	1.4507	0.0000	GRB2-associated binding protein 2
ILMN_2163796	RABGGTB	5876	446	514	491	590	453	631	889	746	1.1018	0.3056	1.1473	0.1592	1.0158	0.8691	1.2274	0.0407	1.9935	0.0000	1.4502	0.0007	Rab geranylgeranyltransferase, beta subunit
ILMN_2147105	NA	NA	248	231	239	245	219	254	322	333	-1.0392	0.6105	1.0629	0.4332	-1.1357	0.1120	1.1017	0.2124	1.2967	0.0020	1.4462	0.0001	NA
ILMN_2404625	LAT	27040	114	109	127	108	110	120	149	157	1.1060	0.1247	-1.0129	0.8626	-1.0374	0.5865	1.1050	0.1385	1.3038	0.0003	1.4432	0.0000	linker for activation of T cells
ILMN_1780291	NFAT5	10725	159	152	176	184	168	167	198	219	1.1123	0.1090	1.2139	0.0057	1.0578	0.3924	1.1032	0.1430	1.2455	0.0020	1.4411	0.0000	nuclear factor of activated T-cells 5, tonicity-responsive
ILMN_1706344	ABHD6	57406	214	246	220	268	205	281	276	353	1.0325	0.6484	1.0924	0.1976	-1.0414	0.5726	1.1461	0.0514	1.2907	0.0012	1.4381	0.0000	abhydrolase domain containing 6
ILMN_1788988	THAP1	55145	197	203	224	235	236	231	276	290	1.1377	0.1623	1.1594	0.1095	1.1962	0.0581	1.1379	0.1588	1.4015	0.0010	1.4307	0.0005	THAP domain containing, apoptosis associated protein 1
ILMN_1706217	TLR4	7099	258	252	262	270	267	310	331	361	1.0165	0.8291	1.0720	0.3368	1.0339	0.6427	1.2294	0.0077	1.2847	0.0017	1.4304	0.0000	toll-like receptor 4
ILMN_1764201	MAP2	4133	124	118	153	126	132	118	196	169	1.2304	0.0117	1.0625	0.4903	1.0572	0.4799	-1.0044	0.9088	1.5781	0.0000	1.4288	0.0001	microtubule-associated protein 2
ILMN_1772946	STRN3	29966	1230	1333	1355	1647	1367	1664	1616	1902	1.1012	0.2084	1.2361	0.0095	1.1116	0.1705	1.2485	0.0070	1.3135	0.0015	1.4273	0.0001	striatin, calmodulin binding protein 3
ILMN_2157020	SNORD48	26801	130	129	148	133	152	155	162	184	1.1394	0.0487	1.0288	0.6637	1.1647	0.0221	1.1972	0.0086	1.2458	0.0018	1.4248	0.0000	small nucleolar RNA, C/D box 48
ILMN_2392143	TRAF6	7189	253	260	296	290	259	256	332	370	1.1709	0.0233	1.1147	0.1078	1.0227	0.7192	-1.0144	0.8242	1.3131	0.0004	1.4221	0.0000	TNF receptor-associated factor 6
ILMN_1810436	DNAJC27	51277	263	297	267	314	264	287	334	422	1.0135	0.8269	1.0567	0.3571	1.0042	0.9394	-1.0359	0.5612	1.2694	0.0006	1.4212	0.0000	DnaJ (Hsp40) homolog, subfamily C, member 27
ILMN_1737308	GLRX	2745	839	849	759	983	758	756	1372	1206	-1.1058	0.2917	1.1578	0.1294	-1.1074	0.2820	-1.1232	0.2291	1.6350	0.0000	1.4204	0.0011	glutaredoxin (thioltransferase)
ILMN_1661833	ANKRD12	23253	384	382	366	440	388	403	622	543	-1.0475	0.5189	1.1508	0.0499	1.0118	0.8632	1.0538	0.4486	1.6193	0.0000	1.4195	0.0000	ankyrin repeat domain 12

ILMN_1779480	KCMF1	56888	140	142	159	164	162	152	189	201	1.1377	0.0518	1.1512	0.0323	1.1537	0.0313	1.0654	0.3242	1.3500	0.0001	1.4099	0.0000	potassium channel modulatory factor 1
ILMN_1810962	PTPRK	5796	2201	2370	2178	2221	2166	2543	4458	3340	-1.0106	0.9169	-1.0668	0.5194	-1.0161	0.8735	1.0731	0.4802	2.0255	0.0000	1.4091	0.0023	protein tyrosine phosphatase, receptor type, K
ILMN_2084073	UCN	7349	143	145	150	147	173	146	199	204	1.0541	0.5354	1.0107	0.8978	1.2153	0.0267	1.0020	0.9793	1.3924	0.0005	1.4059	0.0003	urocortin
ILMN_1810559	RHOQ	23433	5057	5845	4462	5717	4935	5666	6339	8212	-1.1332	0.0637	-1.0225	0.7280	-1.0247	0.7045	-1.0317	0.6263	1.2535	0.0020	1.4049	0.0000	ras homolog gene family, member Q
ILMN_1679268	PELI1	57162	204	197	191	230	206	202	246	276	-1.0727	0.3102	1.1694	0.0319	1.0069	0.9410	1.0228	0.7541	1.2025	0.0121	1.4017	0.0001	pellino homolog 1 (Drosophila)
ILMN_1656904	SLC1A4	6509	139	130	124	119	125	136	173	182	-1.1186	0.2036	-1.0940	0.3179	-1.1107	0.2294	1.0479	0.5826	1.2481	0.0102	1.4000	0.0004	solute carrier family 1 (glutamate/neutral amino acid transporter), member 4
ILMN_1746012	MBD6	114785	594	800	604	685	661	885	915	1118	1.0177	0.8755	-1.1672	0.1261	1.1123	0.3030	1.1069	0.3018	1.5403	0.0003	1.3985	0.0020	methyl-CpG binding domain protein 6
ILMN_1710124	CMTM8	152189	997	1176	829	1098	1121	1149	1527	1641	-1.2030	0.1323	-1.0710	0.5524	1.1244	0.3222	-1.0231	0.8410	1.5313	0.0013	1.3952	0.0078	CKLF-like MARVEL transmembrane domain containing 8
ILMN_2047460	C1orf187	374946	102	99	102	101	123	114	198	138	1.0028	0.9709	1.0152	0.8460	1.2067	0.0147	1.1429	0.0772	1.9436	0.0000	1.3936	0.0001	chromosome 1 open reading frame 187
ILMN_2374293	DYRK1A	1859	548	511	567	601	523	591	758	711	1.0341	0.5765	1.1760	0.0125	-1.0478	0.4359	1.1558	0.0236	1.3817	0.0000	1.3913	0.0000	dual-specificity tyrosine-(Y)-phosphorylation regulated kinase 1A
ILMN_1752478	DHRS3	9249	980	1247	993	1590	1329	1358	1338	1733	1.0134	0.8967	1.2748	0.0299	1.3560	0.0091	1.0885	0.4201	1.3655	0.0079	1.3898	0.0047	dehydrogenase/reductase (SDR family) member 3
ILMN_1680693	ZNF419	79744	342	415	307	383	328	371	480	576	-1.1126	0.1314	-1.0839	0.2235	-1.0423	0.5460	-1.1190	0.0971	1.4039	0.0000	1.3890	0.0000	zinc finger protein 419
ILMN_1694475	GTPBP2	54676	144	156	144	139	139	141	193	216	1.0015	0.9820	-1.1247	0.0819	-1.0395	0.5601	-1.1043	0.1483	1.3394	0.0001	1.3876	0.0000	GTP binding protein 2
ILMN_1662243	ING1	3621	271	279	286	297	288	316	400	386	1.0569	0.5872	1.0650	0.5163	1.0635	0.5368	1.1331	0.2106	1.4758	0.0006	1.3868	0.0024	inhibitor of growth family, member 1
ILMN_1741847	MMP10	4319	101	103	103	113	97	102	130	143	1.0199	0.7702	1.1007	0.1664	-1.0405	0.5781	-1.0051	0.9434	1.2882	0.0009	1.3846	0.0001	matrix metallopeptidase 10 (stromelysin 2)
ILMN_2212590	TMEM170A	124491	245	305	265	364	283	302	363	421	1.0803	0.2949	1.1929	0.0155	1.1570	0.0533	-1.0094	0.8856	1.4818	0.0000	1.3794	0.0001	transmembrane protein 170A
ILMN_1724062	LIN54	132660	244	241	300	281	308	264	329	332	1.2293	0.0186	1.1643	0.0759	1.2581	0.0095	1.0949	0.2822	1.3459	0.0013	1.3748	0.0006	lin-54 homolog (C. elegans)
ILMN_1677240	SLC10A7	84068	110	126	121	140	131	140	141	173	1.0960	0.1555	1.1078	0.0873	1.1944	0.0085	1.1107	0.0857	1.2769	0.0006	1.3681	0.0000	solute carrier family 10 (sodium/bile acid cotransporter family), member 7
ILMN_1777322	FAM91A1	157769	285	338	300	351	298	314	335	461	1.0539	0.3561	1.0383	0.4805	1.0457	0.4296	-1.0758	0.1867	1.1750	0.0076	1.3659	0.0000	family with sequence similarity 91, member A1
ILMN_1781173	HDAC9	9734	120	110	116	103	109	108	181	150	-1.0353	0.7393	-1.0706	0.5195	-1.0972	0.3727	-1.0194	0.8629	1.5111	0.0002	1.3592	0.0039	histone deacetylase 9
ILMN_1709399	SLC25A34	284723	128	112	119	117	114	110	151	152	-1.0790	0.1824	1.0485	0.4220	-1.1225	0.0520	-1.0151	0.8077	1.1842	0.0049	1.3560	0.0000	solute carrier family 25, member 34
ILMN_1779177	U2AF1L4	199746	296	313	310	316	343	313	372	423	1.0475	0.4794	1.0073	0.9100	1.1580	0.0329	1.0004	0.9912	1.2570	0.0017	1.3497	0.0001	U2 small nuclear RNA auxiliary factor 1-like 4
ILMN_1709549	PLEKHM1	9842	273	256	269	250	258	268	400	345	-1.0149	0.8357	-1.0234	0.7724	-1.0572	0.4680	1.0482	0.5550	1.4638	0.0000	1.3479	0.0007	pleckstrin homology domain containing, family M (with RUN domain) member 1
ILMN_1662686	USPL1	10208	192	195	214	234	182	225	257	262	1.1114	0.2035	1.2000	0.0313	-1.0552	0.5223	1.1546	0.0823	1.3358	0.0014	1.3463	0.0009	ubiquitin specific peptidase like 1
ILMN_1686846	AKAP12	9590	126	114	139	119	137	131	194	153	1.0964	0.2574	1.0395	0.6481	1.0817	0.3224	1.1488	0.1059	1.5347	0.0000	1.3431	0.0012	A kinase (PRKA) anchor protein 12
ILMN_1669376	DRAM1	55332	736	622	723	612	691	548	1127	836	-1.0191	0.7627	-1.0159	0.8048	-1.0656	0.3191	-1.1360	0.0587	1.5303	0.0000	1.3428	0.0001	DNA-damage regulated autophagy modulator 1
ILMN_1664560	DYRK1A	1859	602	597	618	646	572	690	793	801	1.0267	0.7185	1.0824	0.2703	-1.0525	0.4740	1.1569	0.0515	1.3170	0.0008	1.3423	0.0004	dual-specificity tyrosine-(Y)-phosphorylation regulated kinase 1A
ILMN_1718831	TMEM57	55219	220	359	246	373	240	343	330	482	1.1160	0.0691	1.0386	0.4633	1.0898	0.1471	-1.0493	0.3516	1.4990	0.0000	1.3419	0.0000	transmembrane protein 57
ILMN_1733115	RALGAPB	57148	335	375	343	374	326	380	401	502	1.0235	0.6599	-1.0016	0.9715	-1.0269	0.6063	1.0132	0.8018	1.1955	0.0020	1.3409	0.0000	Ral GTPase activating protein, beta subunit (non-catalytic)
ILMN_1692335	ELK3	2004	167	159	186	191	204	189	214	213	1.1167	0.0973	1.1989	0.0103	1.2238	0.0044	1.1889	0.0140	1.2870	0.0006	1.3382	0.0002	ELK3, ETS-domain protein (SRF accessory protein 2)
ILMN_1685567	TGDS	23483	359	386	414	446	380	419	495	515	1.1509	0.0229	1.1557	0.0178	1.0568	0.3450	1.0845	0.1633	1.3778	0.0000	1.3348	0.0000	TDP-glucose 4,6-dehydratase
ILMN_1694269	CCDC93	54520	111	117	122	129	105	125	138	156	1.1001	0.1165	1.1068	0.0947	-1.0569	0.3752	1.0705	0.2553	1.2426	0.0012	1.3299	0.0001	coiled-coil domain containing 93
ILMN_1742074	C8orf76	84933	1411	1544	1366	1646	1556	1707	1790	2039	-1.0328	0.5144	1.0658	0.2000	1.1024	0.0572	1.1059	0.0491	1.2685	0.0001	1.3208	0.0000	chromosome 8 open reading frame 76
ILMN_1797362	LIMK1	3984	191	176	217	177	203	199	230	231	1.1336	0.0360	1.0056	0.9281	1.0609	0.3166	1.1351	0.0421	1.2045	0.0036	1.3127	0.0001	LIM domain kinase 1
ILMN_2294684	CEP170	9859	142	161	170	194	177	177	191	211	1.2015	0.0289	1.2043	0.0207	1.2480	0.0098	1.0988	0.2147	1.3496	0.0008	1.3120	0.0013	centrosomal protein 170kDa
ILMN_1723260	ETV5	2119	119	122	132	127	124	126	162	160	1.1069	0.1071	1.0427	0.4940	1.0367	0.5627	1.0375	0.5418	1.3580	0.0000	1.3120	0.0001	ets variant 5
ILMN_1676305	PTK2	5747	158	154	169	176	187	172	205	201	1.0721	0.2351	1.1436	0.0275	1.1850	0.0063	1.1176	0.0646	1.2976	0.0001	1.3088	0.0001	PTK2 protein tyrosine kinase 2
ILMN_1815313	EPGN	255324	172	203	183	206	206	193	222	266	1.0697	0.4465	1.0119	0.8913	1.2022	0.0418	-1.0530	0.5439	1.2959	0.0058	1.3077	0.0031	epithelial mitogen homolog (mouse)
ILMN_1779492	SMURF1	57154	128	138	139	153	127	150	160	181	1.0844	0.1541	1.1057	0.0789	-1.0090	0.8775	1.0825	0.1624	1.2509	0.0005	1.3072	0.0001	SMAD specific E3 ubiquitin protein ligase 1
ILMN_1708077	DAXX	1616	366	395	390	349	415	418	433	516	1.0657	0.3192	-1.1303	0.0692	1.1344	0.0593	1.0592	0.3596	1.1827	0.0148	1.3059	0.0003	death-domain associated protein
ILMN_1736048	ELL	8178	255	244	230	251	245	266	316	319	-1.1076	0.1138	1.0271	0.6570	-1.0380	0.5471	1.0880	0.1754	1.2389	0.0015	1.3056	0.0002	elongation factor RNA polymerase II
ILMN_1680782	PATL1	219988	1453	1421	1483	1449	1631	1390	2015	1854	1.0210	0.7939	1.0197	0.8121	1.1225	0.1539	-1.0219	0.7818	1.3872	0.0005	1.3048	0.0029	protein associated with topoisomerase II homolog 1 (yeast)
ILMN_2183687	LIME1	54923	257	260	282	297	271	278	332	339	1.0994	0.1621	1.1403	0.0528	1.0556	0.4195	1.0684	0.3124	1.2960	0.0006	1.3039	0.0004	Lck interacting transmembrane adaptor 1
ILMN_1683682	DAGLA	747	144	156	155	188	152	168	179	203	1.0768	0.2372	1.2055	0.0040	1.0607	0.3451	1.0783	0.2077	1.2474	0.0013	1.3022	0.0002	diacylglycerol lipase, alpha
ILMN_2232157	SLMO1	10650	338	366	386	351	334	314	435	474	1.1393	0.1361	-1.0438	0.6183	-1.0123	0.8888	-1.1645	0.0874	1.2849	0.0066	1.2958	0.0045	slowmo homolog 1 (Drosophila)
ILMN_1752394	CCNB1IP1	57820	767	695	796	706	867	681	960	899	1.0376	0.4683	1.0169	0.7437	1.1301	0.0234	-1.0205	0.6912	1.2517	0.0002	1.2945	0.0001	cyclin B1 interacting protein 1
ILMN_1658144	TRIP11	9321	515	672	548	778	614	796	698	869	1.0623	0.4306	1.1581	0.0564	1.1915	0.0307	1.1840	0.0295	1.3535	0.0006	1.2924	0.0020	thyroid hormone receptor interactor 11
ILMN_2414533	NA	NA	665	832	675	835	758	875	813	1075	1.0148	0.7957	1.0031	0.9562	1.1384	0.0304	1.0519	0.3549	1.2214	0.0018	1.2918	0.0001	NA
ILMN_1687501	MOXD1	26002	1601	1162	1737	1260	1611	1325	2079	1499	1.0845	0.1568	1.0839	0.1705	1.0060	0.9146	1.1397	0.0317	1.2981	0.0001	1.2894	0.0002	monooxygenase, DBH-like 1
ILMN_1731644	SETDB2	83852	175	187	167	199	179	171	221	241	-1.0512	0.5349	1.0656	0.4288	1.0203	0.8147	-1.0937	0.2741	1.2594	0.0076	1.2894	0.0031	SET domain, bifurcated 2
ILMN_1769071	XKRX	402415	107	112	114	114	107	107	146	145	1.0661	0.2666	1.0177	0.7532	1.0045	0.9409	-1.0498	0.4047	1.3672	0.0000	1.2883	0.0001	XK, Kell blood group complex subunit-related, X-linked
ILMN_1694547	IFFO1	25900	122	121	149	132	123	113	171	155	1.2249	0.0149	1.0911	0.2755	1.0119	0.8806	-1.0668	0.4414	1.4029	0.0002	1.2859	0.0031	intermediate filament family orphan 1
ILMN_1780824	CDK17	5128	121	118	127	139	141	132	170	151	1.0523	0.3919	1.1729	0.0114	1.1665	0.0135	1.1136	0.0764	1.4099	0.0000	1.2784	0.0003	cyclin-dependent kinase 17
ILMN_2360291	UGGT1	56886	495	573	568	680	544	614	625	732	1.1469	0.0515	1.1865	0.0156	1.0980	0.1761	1.0728	0.2922	1.2617	0.0021	1.2774	0.0011	UDP-glucose glycoprotein glucosyltransferase 1
ILMN_1690754	SVIL	6840	541	641	470	606	553	630	725	816	-1.1500	0.0567	-1.0577	0.4106	1.0215	0.7751	-1.0167	0.8048	1.3397	0.0003	1.2736	0.0015	supervillin
ILMN_1691444	UHRF2	115426	161	157	173	180	174	178	216	200	1.0783	0.2392	1.1437	0.0431	1.0826	0.2158	1.1288	0.0695	1.3445	0.0001	1.2701	0.0009	ubiquitin-like with PHD and ring finger domains 2
ILMN_1728467	GHITM	27069	13362	14340	13996	15908	14430	15355	16026	18207	1.0475	0.3160	1.1093	0.0326	1.0800	0.1039	1.0708	0.1451	1.1994	0.0007	1.2697	0.0000	growth hormone inducible transmembrane protein
ILMN_1756455	IL5RA	3568	127	145	146	166	156	149	165	184	1.1435	0.0544	1.1468	0.0436	1.2281	0.0053	1.0325	0.6348	1.2937	0.0008	1.2697	0.0011	interleukin 5 receptor, alpha
ILMN_1809285	DCP1A	55802	242	276	258	257	257	284	312	349	1.0650	0.3485	-1.0706	0.3037	1.0614	0.3738	1.0292	0.6540	1.2882	0.0007	1.2670	0.0010	DCP1 decapping enzyme homolog A (S. cerevisiae)
ILMN_1716524	RAB7A	7879	4760	5079	4366	5065	5035	5498	5540	6431	-1.0902	0.0887	-1.0027	0.9558	1.0576	0.2588	1.0825	0.1149	1.1637	0.0052	1.2664	0.0001	RAB7A, member RAS oncogene family
ILMN_1794074	MXI1	4601	140	140	133	150	136	137	174	177	-1.0548	0.3208	1.0750	0.1765	-1.0339	0.5316	-1.0210	0.6972	1.2411	0.0003	1.2654	0.0001	MAX interactor 1
ILMN_1652024	CSNK1G3	1456	505	503	460	563	558	548	593	636	-1.0975	0.1083	1.1186	0.0531	1.1056	0.0793	1.0888	0.1340	1.1749	0.0075	1.2649	0.0003	casein kinase 1, gamma 3
ILMN_1765409	STAM	8027	1079	1188	1134	1198	1253	1216	1454	1502	1.0507	0.3978	1.0087	0.8783	1.1614	0.0165	1.0235	0.6862	1.3477	0.0000	1.2644	0.0005	signal transducing adaptor molecule (SH3 domain and ITAM motif) 1
ILMN_1810191	PLA2G4C	8605	138	154	130	153	145	136	171	194	-1.0608	0.3670	-1.0054	0.9438	1.0557	0.3968	-1.1302	0.0596	1.2448	0.0017	1.2634	0.0006	phospholipase A2, group IVC (cytosolic, calcium-independent)
ILMN_1691290	CELSR3	1951	389	427	403	434	377	409	492	539	1.0346	0.6698	1.0175	0.8251	-1.0332	0.6699	-1.0445	0.5607	1.2634	0.0056	1.2623	0.0051	cadherin, EGF LAG seven-pass G-type receptor 3 (flamingo homolog, Drosophila)
ILMN_1663444	LIN7B	64130	231	306	249	354	273	333	307	385	1.0813	0.2365	1.1572	0.0220	1.1841	0.0148	1.0860	0.1790	1.3299	0.0002	1.2580	0.0008	lin-7 homolog B (C. elegans)
ILMN_1788251	SNN	8303	131	140	147	145	135	144	164	175	1.1249	0.0828	1.0357	0.5842	1.0353	0.6029	1.0332	0.5996	1.2575	0.0016	1.2517	0.0015	stannin
ILMN_1735798	OSBPL3	26031	137	131	150	146	158	135	182	164	1.0915	0.1288	1.1106	0.0713	1.1556	0.0155	1.0258	0.6689	1.3289	0.0000	1.2502	0.0006	oxysterol binding protein-like 3
ILMN_1769013	ASGR1	432	156	166	156	186	178	158	194	206	1.0024	0.9756	1.1248	0.0587	1.1410	0.0383	-1.0475	0.4354	1.2413	0.0014	1.2422	0.0012	asialoglycoprotein receptor 1
ILMN_1673024	RBM15B	29890	231	218	279	254	252	238	296	270	1.2077	0.0050	1.1679	0.0189	1.0880	0.1813	1.0908	0.1762	1.2780	0.0005	1.2415	0.0019	RNA binding motif protein 15B
ILMN_1745686	MFHAS1	9258	112	110	113	107	116	114	150	137	1.0051	0.9585	-1.0356	0.6072	1.0345	0.6315	1.0325	0.6347	1.3384	0.0002	1.2391	0.0028	malignant fibrous histiocytoma amplified sequence 1
ILMN_1692352	IRAK4	51135	189	192	210	224	203	209	229	238	1.1125	0.0491	1.1654	0.0066	1.0720	0.1913	1.0848	0.1273	1.2106	0.0011	1.2380	0.0004	interleukin-1 receptor-associated kinase 4
ILMN_2225577	C5orf37	134359	339	386	386	429	396	430	434	477	1.1371	0.0360	1.1111	0.0738	1.1667	0.0137	1.1149	0.0655	1.2778	0.0003	1.2357	0.0010	chromosome 5 open reading frame 37
ILMN_1682636	CXCL2	2920	99	106	107	109	100	103	125	131	1.0845	0.2744	1.0274	0.7076	1.0054	0.9423	-1.0299	0.6932	1.2618	0.0033	1.2349	0.0046	chemokine (C-X-C motif) ligand 2
ILMN_1782292	LAMP1	3916	7288	6687	7403	6564	7314	6846	9205	8252	1.0159	0.7736	-1.0186	0.7367	1.0037	0.9465	1.0238	0.6677	1.2631	0.0003	1.2342	0.0009	lysosomal-associated membrane protein 1
ILMN_2402363	STK19	8859	1332	1410	1549	1556	1323	1330	1673	1738	1.1625	0.0581	1.1040	0.2000	-1.0072	0.9201	-1.0602	0.4446	1.2561	0.0064	1.2331	0.0108	serine/threonine kinase 19
ILMN_1660533	RPN1	6184	6151	7443	7171	8425	6183	8309	7486	9155	1.1657	0.0084	1.1319	0.0280	1.0052	0.9215	1.1164	0.0481	1.2170	0.0013	1.2300	0.0008	ribophorin I
ILMN_1728802	SDCCAG8	10806	221	240	253	260	228	232	295	294	1.1454	0.0284	1.0840	0.1719	1.0330	0.5771	-1.0318	0.5840	1.3355	0.0001	1.2289	0.0014	serologically defined colon cancer antigen 8
ILMN_2356654	LGALS8	3964	437	438	417	510	387	476	530	538	-1.0477	0.5519	1.1634	0.0523	-1.1276	0.1275	1.0868	0.2698	1.2142	0.0153	1.2283	0.0104	lectin, galactoside-binding, soluble, 8
ILMN_2071826	RNF152	220441	110	111	117	125	117	123	134	135	1.0668	0.2214	1.1321	0.0226	1.0650	0.2367	1.1102	0.0510	1.2197	0.0008	1.2160	0.0008	ring finger protein 152
ILMN_1764383	MCOLN1	57192	456	562	444	484	488	536	569	677	-1.0272	0.7380	-1.1615	0.0636	1.0717	0.3792	-1.0482	0.5336	1.2480	0.0088	1.2044	0.0203	mucolipin 1
ILMN_1657769	EIF5	1983	108	112	118	111	114	117	138	135	1.0944	0.1108	-1.0129	0.8326	1.0560	0.3319	1.0400	0.4712	1.2810	0.0001	1.2038	0.0019	eukaryotic translation initiation factor 5
ILMN_1652790	CLK1	1195	140	137	141	141	142	130	170	165	1.0119	0.8297	1.0309	0.5662	1.0168	0.7550	-1.0555	0.3252	1.2160	0.0010	1.1995	0.0022	CDC-like kinase 1
ILMN_2382126	PPFIA1	8500	333	335	296	312	344	351	418	401	-1.1240	0.1097	-1.0718	0.3395	1.0338	0.6303	1.0481	0.4943	1.2567	0.0029	1.1984	0.0138	protein tyrosine phosphatase, receptor type, f polypeptide (PTPRF), interacting protein (liprin), alpha 1
ILMN_1773407	C16orf72	29035	856	1023	807	917	873	960	1114	1213	-1.0602	0.4114	-1.1150	0.1275	1.0205	0.7676	-1.0653	0.3636	1.3023	0.0010	1.1859	0.0207	chromosome 16 open reading frame 72
ILMN_1789136	SERF2	10169	10661	10392	10897	10323	11400	10519	12720	12310	1.0222	0.6433	-1.0066	0.8894	1.0694	0.1667	1.0123	0.7965	1.1932	0.0012	1.1846	0.0017	small EDRK-rich factor 2
ILMN_2094416	NA	NA	153	132	142	131	141	136	183	154	-1.0795	0.1466	-1.0039	0.9454	-1.0867	0.1195	1.0330	0.5426	1.1932	0.0016	1.1664	0.0075	NA
ILMN_2061565	PLCH2	9651	135	146	148	134	144	141	179	169	1.0926	0.0930	-1.0872	0.1130	1.0641	0.2339	-1.0326	0.5452	1.3227	0.0000	1.1589	0.0061	phospholipase C, eta 2
ILMN_1683959	MED13L	23389	156	145	149	157	143	163	180	168	-1.0436	0.4328	1.0827	0.1495	-1.0899	0.1224	1.1230	0.0398	1.1567	0.0096	1.1572	0.0118	mediator complex subunit 13-like
ILMN_1741628	ABL2	27	154	145	156	131	160	146	188	166	1.0108	0.8251	-1.1085	0.0538	1.0359	0.4715	1.0076	0.8653	1.2183	0.0004	1.1449	0.0105	v-abl Abelson murine leukemia viral oncogene homolog 2 (arg, Abelson-related gene)
ILMN_1728106	TNF	7124	107	119	119	132	106	107	136	136	1.1068	0.1247	1.1113	0.0898	-1.0179	0.7822	-1.1075	0.1200	1.2646	0.0010	1.1437	0.0341	tumor necrosis factor (TNF superfamily, member 2)
ILMN_1774272	ESRRA	2101	212	230	219	230	192	225	248	258	1.0351	0.4672	-1.0007	1.0000	-1.1035	0.0543	-1.0197	0.6754	1.1683	0.0031	1.1230	0.0181	estrogen-related receptor alpha

**Downregulated genes**																							
ILMN_1779423	MRPS14	63931	236	238	238	257	255	226	202	213	1.0095	0.8489	1.0796	0.1358	1.0779	0.1475	-1.0546	0.3124	-1.1679	0.0072	-1.1152	0.0472	mitochondrial ribosomal protein S14
ILMN_1790461	C6orf125	84300	3636	3735	3758	3440	4053	3688	3202	3293	1.0334	0.5438	-1.0856	0.1386	1.1145	0.0547	-1.0127	0.8164	-1.1358	0.0270	-1.1340	0.0287	chromosome 6 open reading frame 125
ILMN_2178244	NAA40	79829	152	150	144	135	155	162	133	130	-1.0553	0.3291	-1.1141	0.0640	1.0192	0.7275	1.0795	0.1676	-1.1426	0.0256	-1.1509	0.0205	N(alpha)-acetyltransferase 40, NatD catalytic subunit, homolog (S. cerevisiae)
ILMN_1770936	COQ5	84274	2775	3056	2777	2806	2880	3163	2388	2619	1.0006	0.9876	-1.0889	0.0891	1.0379	0.4439	1.0353	0.4735	-1.1621	0.0053	-1.1668	0.0043	coenzyme Q5 homolog, methyltransferase (S. cerevisiae)
ILMN_1789364	ZNF789	285989	171	155	180	164	157	155	134	132	1.0557	0.2981	1.0585	0.2941	-1.0845	0.1357	-1.0035	0.9612	-1.2702	0.0003	-1.1713	0.0095	zinc finger protein 789
ILMN_1718950	ZNF433	163059	145	136	128	135	140	126	114	116	-1.1329	0.0151	-1.0049	0.9203	-1.0344	0.4683	-1.0766	0.1371	-1.2687	0.0001	-1.1714	0.0041	zinc finger protein 433
ILMN_1812552	ACER3	55331	581	739	610	852	523	673	490	628	1.0497	0.5298	1.1540	0.0619	-1.1103	0.1816	-1.0972	0.2261	-1.1852	0.0377	-1.1760	0.0399	alkaline ceramidase 3
ILMN_1699859	GNPAT	8443	1166	1202	1039	1186	1229	1235	941	1019	-1.1215	0.0699	-1.0135	0.8239	1.0545	0.3857	1.0277	0.6487	-1.2389	0.0020	-1.1802	0.0121	glyceronephosphate O-acyltransferase
ILMN_1761764	ALKBH7	84266	526	543	452	473	603	543	438	449	-1.1614	0.0732	-1.1458	0.0997	1.1466	0.0899	1.0009	0.9789	-1.2001	0.0326	-1.2077	0.0281	alkB, alkylation repair homolog 7 (E. coli)
ILMN_1693597	ZNF287	57336	121	114	117	114	116	113	104	94	-1.0353	0.4459	-1.0023	0.9633	-1.0479	0.3110	-1.0086	0.8456	-1.1712	0.0026	-1.2097	0.0007	zinc finger protein 287
ILMN_1740165	C14orf102	55051	742	690	639	740	658	583	547	570	-1.1616	0.0380	1.0729	0.3045	-1.1273	0.0907	-1.1831	0.0231	-1.3582	0.0002	-1.2106	0.0113	chromosome 14 open reading frame 102
ILMN_1725760	YTHDC2	64848	188	203	209	226	183	183	154	167	1.1083	0.1803	1.1156	0.1369	-1.0319	0.6547	-1.1080	0.1719	-1.2204	0.0157	-1.2127	0.0172	YTH domain containing 2
ILMN_1729816	VDAC3	7419	11145	11651	11017	10564	11044	11596	8839	9595	-1.0117	0.7551	-1.1029	0.0152	-1.0092	0.8067	-1.0047	0.9000	-1.2610	0.0000	-1.2142	0.0000	voltage-dependent anion channel 3
ILMN_2383693	UPF2	26019	1533	1444	1335	1497	1359	1334	1275	1189	-1.1483	0.0256	1.0364	0.5387	-1.1278	0.0486	-1.0829	0.1797	-1.2021	0.0045	-1.2150	0.0031	UPF2 regulator of nonsense transcripts homolog (yeast)
ILMN_1728230	ZNF30	90075	169	186	163	198	173	178	144	153	-1.0360	0.5149	1.0648	0.2230	1.0255	0.6415	-1.0444	0.4208	-1.1745	0.0087	-1.2164	0.0016	zinc finger protein 30
ILMN_1771746	C6orf165	154313	167	190	139	189	138	173	127	155	-1.2085	0.0075	-1.0028	0.9594	-1.2173	0.0058	-1.0984	0.1393	-1.3143	0.0004	-1.2217	0.0036	chromosome 6 open reading frame 165
ILMN_1712455	RBM4	5936	981	1055	978	1074	903	960	796	861	-1.0031	0.9519	1.0185	0.7696	-1.0855	0.2053	-1.0991	0.1490	-1.2313	0.0038	-1.2249	0.0045	RNA binding motif protein 4
ILMN_2400292	MAPK9	5601	866	912	759	834	882	967	705	741	-1.1409	0.0378	-1.0937	0.1437	1.0187	0.7592	1.0604	0.3267	-1.2291	0.0025	-1.2314	0.0023	mitogen-activated protein kinase 9
ILMN_1654612	ZNF589	51385	156	155	152	133	149	144	124	126	-1.0226	0.6500	-1.1710	0.0056	-1.0449	0.3840	-1.0774	0.1491	-1.2591	0.0002	-1.2335	0.0005	zinc finger protein 589
ILMN_1669905	DCP2	167227	639	589	563	599	574	631	472	475	-1.1365	0.0618	1.0185	0.7804	-1.1146	0.1081	1.0723	0.2855	-1.3540	0.0002	-1.2383	0.0043	DCP2 decapping enzyme homolog (S. cerevisiae)
ILMN_2363361	SFXN4	119559	635	684	619	603	652	685	496	551	-1.0255	0.6763	-1.1343	0.0477	1.0265	0.6719	1.0013	0.9836	-1.2797	0.0007	-1.2426	0.0018	sideroflexin 4
ILMN_1802053	ZNF91	7644	156	147	134	136	131	134	126	118	-1.1686	0.0094	-1.0806	0.1637	-1.1930	0.0037	-1.0927	0.1140	-1.2434	0.0007	-1.2429	0.0009	zinc finger protein 91
ILMN_2179652	PRELID1	27166	3357	3910	3502	3440	3168	3350	2718	3137	1.0431	0.5026	-1.1366	0.0511	-1.0597	0.3592	-1.1674	0.0210	-1.2354	0.0029	-1.2464	0.0020	PRELI domain containing 1
ILMN_1658080	ZNF573	126231	220	226	186	238	199	219	157	181	-1.1872	0.0216	1.0535	0.4446	-1.1100	0.1386	-1.0309	0.6494	-1.4069	0.0001	-1.2482	0.0044	zinc finger protein 573
ILMN_1753515	SRR	63826	312	280	295	273	307	260	236	224	-1.0577	0.3382	-1.0271	0.6449	-1.0166	0.7692	-1.0759	0.2199	-1.3229	0.0001	-1.2517	0.0012	serine racemase
ILMN_1807710	HINT1	3094	28756	27340	24438	24287	24560	22387	21786	21832	-1.1767	0.0179	-1.1257	0.0750	-1.1709	0.0212	-1.2213	0.0049	-1.3199	0.0003	-1.2523	0.0020	histidine triad nucleotide binding protein 1
ILMN_2391345	C9orf23	138716	211	189	181	170	220	201	167	151	-1.1650	0.0584	-1.1129	0.1849	1.0413	0.5545	1.0634	0.4326	-1.2668	0.0062	-1.2557	0.0110	chromosome 9 open reading frame 23
ILMN_1739497	GTF2H5	404672	6013	5135	5530	5125	6375	5408	4721	4076	-1.0874	0.1788	-1.0020	0.9740	1.0601	0.3419	1.0531	0.4005	-1.2737	0.0007	-1.2600	0.0011	general transcription factor IIH, polypeptide 5
ILMN_2155708	TLR3	7098	126	123	112	122	108	102	97	98	-1.1238	0.0908	-1.0111	0.8841	-1.1627	0.0325	-1.2139	0.0090	-1.3026	0.0009	-1.2611	0.0026	toll-like receptor 3
ILMN_1684352	TRIM45	80263	194	191	175	163	177	171	151	151	-1.1085	0.0626	-1.1720	0.0069	-1.0977	0.0874	-1.1153	0.0495	-1.2828	0.0002	-1.2626	0.0003	tripartite motif-containing 45
ILMN_1719449	DCLK2	166614	192	192	192	183	160	160	146	152	1.0013	0.9991	-1.0498	0.5053	-1.2012	0.0235	-1.2017	0.0226	-1.3182	0.0018	-1.2643	0.0055	doublecortin-like kinase 2
ILMN_2155998	PSMD6	9861	6308	6213	5538	5394	5213	6072	5045	4904	-1.1392	0.0450	-1.1519	0.0309	-1.2100	0.0054	-1.0232	0.7089	-1.2503	0.0016	-1.2668	0.0010	proteasome (prosome, macropain) 26S subunit, non-ATPase, 6
ILMN_1742889	WDR77	79084	451	494	406	432	431	469	319	389	-1.1090	0.0653	-1.1417	0.0205	-1.0461	0.4006	-1.0522	0.3384	-1.4114	0.0000	-1.2679	0.0002	WD repeat domain 77
ILMN_1771987	SLC44A2	57153	2246	2058	2000	1808	2225	2200	1649	1620	-1.1234	0.1104	-1.1381	0.0803	-1.0096	0.8875	1.0690	0.3499	-1.3626	0.0003	-1.2704	0.0029	solute carrier family 44, member 2
ILMN_1716583	NME7	29922	1511	1504	1407	1594	1313	1336	1189	1177	-1.0737	0.3020	1.0600	0.3973	-1.1503	0.0508	-1.1254	0.0933	-1.2707	0.0021	-1.2783	0.0017	non-metastatic cells 7, protein expressed in (nucleoside-diphosphate kinase)
ILMN_1762167	GTDC1	79712	217	218	207	207	193	191	174	170	-1.0526	0.2798	-1.0515	0.2904	-1.1281	0.0188	-1.1408	0.0108	-1.2469	0.0002	-1.2809	0.0001	glycosyltransferase-like domain containing 1
ILMN_1713978	SDF2	6388	2530	2633	2439	2845	2249	2289	1972	2052	-1.0372	0.5573	1.0803	0.2171	-1.1248	0.0686	-1.1505	0.0324	-1.2824	0.0006	-1.2831	0.0006	stromal cell-derived factor 2
ILMN_1668795	TTC30B	150737	157	144	131	127	127	135	121	112	-1.1913	0.0164	-1.1325	0.0811	-1.2336	0.0055	-1.0665	0.3304	-1.2941	0.0012	-1.2849	0.0019	tetratricopeptide repeat domain 30B
ILMN_2050023	CCDC23	374969	2093	1999	2090	1887	2169	1740	1673	1555	-1.0017	0.9815	-1.0594	0.3877	1.0361	0.5909	-1.1492	0.0471	-1.2508	0.0029	-1.2854	0.0011	coiled-coil domain containing 23
ILMN_1667199	SQRDL	58472	414	432	397	374	399	368	328	334	-1.0419	0.5138	-1.1562	0.0312	-1.0362	0.5675	-1.1756	0.0178	-1.2602	0.0017	-1.2951	0.0006	sulfide quinone reductase-like (yeast)
ILMN_1724734	UQCC	55245	277	291	273	282	261	256	219	225	-1.0172	0.7835	-1.0337	0.6090	-1.0631	0.3563	-1.1383	0.0574	-1.2662	0.0020	-1.2966	0.0008	ubiquinol-cytochrome c reductase complex chaperone
ILMN_1652753	PAAF1	80227	887	885	839	810	888	858	602	681	-1.0569	0.2432	-1.0922	0.0713	1.0013	0.9750	-1.0306	0.5205	-1.4730	0.0000	-1.2990	0.0000	proteasomal ATPase-associated factor 1
ILMN_1702363	SULF1	23213	185	149	172	135	191	135	143	115	-1.0727	0.1932	-1.1005	0.1133	1.0313	0.5633	-1.1003	0.1148	-1.2973	0.0001	-1.3015	0.0003	sulfatase 1
ILMN_2073012	TMEM203	94107	2486	2269	2388	2026	2827	2201	1890	1740	-1.0412	0.5201	-1.1200	0.0814	1.1371	0.0486	-1.0307	0.6283	-1.3158	0.0003	-1.3040	0.0004	transmembrane protein 203
ILMN_1759460	TAF7	6879	549	454	498	511	453	435	394	348	-1.1023	0.2455	1.1255	0.1756	-1.2139	0.0297	-1.0438	0.6037	-1.3942	0.0008	-1.3052	0.0057	TAF7 RNA polymerase II, TATA box binding protein (TBP)-associated factor, 55kDa
ILMN_2359211	AP2A1	160	163	145	143	113	147	131	120	111	-1.1406	0.0726	-1.2829	0.0037	-1.1081	0.1527	-1.1094	0.1926	-1.3605	0.0004	-1.3053	0.0023	adaptor-related protein complex 2, alpha 1 subunit
ILMN_1753719	GTF2A2	2958	4304	4299	3782	4156	3590	3957	2965	3274	-1.1382	0.0380	-1.0345	0.5661	-1.1989	0.0056	-1.0865	0.1691	-1.4519	0.0000	-1.3134	0.0002	general transcription factor IIA, 2, 12kDa
ILMN_1758642	NA	NA	202	213	199	186	181	198	144	162	-1.0154	0.8075	-1.1435	0.0285	-1.1155	0.0743	-1.0719	0.2256	-1.4061	0.0000	-1.3139	0.0002	NA
ILMN_2288070	FTO	79068	476	488	431	378	428	401	361	371	-1.1048	0.2165	-1.2925	0.0037	-1.1118	0.1852	-1.2170	0.0201	-1.3186	0.0021	-1.3174	0.0021	fat mass and obesity associated
ILMN_1677824	NA	NA	446	461	417	448	412	405	366	349	-1.0688	0.2067	-1.0289	0.5773	-1.0820	0.1419	-1.1374	0.0205	-1.2182	0.0012	-1.3193	0.0000	NA
ILMN_1680390	GCNT2	2651	331	478	293	434	307	431	263	362	-1.1281	0.0372	-1.1012	0.0650	-1.0781	0.1743	-1.1080	0.0517	-1.2588	0.0004	-1.3213	0.0000	glucosaminyl (N-acetyl) transferase 2, I-branching enzyme (I blood group)
ILMN_2206098	ATG10	83734	337	363	342	343	317	295	267	274	1.0147	0.8573	-1.0580	0.4570	-1.0647	0.3966	-1.2335	0.0113	-1.2647	0.0058	-1.3258	0.0014	ATG10 autophagy related 10 homolog (S. cerevisiae)
ILMN_1701512	KIAA0391	9692	1627	1782	1632	1720	1592	1571	1265	1344	1.0028	0.9501	-1.0362	0.4414	-1.0220	0.6374	-1.1345	0.0116	-1.2859	0.0000	-1.3265	0.0000	KIAA0391
ILMN_2317348	APTX	54840	957	1125	883	1026	944	1104	757	848	-1.0837	0.2492	-1.0968	0.1777	-1.0133	0.8455	-1.0188	0.7791	-1.2638	0.0026	-1.3266	0.0004	aprataxin
ILMN_2152178	MTMR15	22909	275	246	233	211	244	222	195	185	-1.1847	0.0115	-1.1639	0.0274	-1.1292	0.0577	-1.1064	0.1239	-1.4108	0.0000	-1.3281	0.0003	myotubularin related protein 15
ILMN_1677793	P2RX5	5026	156	153	150	143	139	131	128	115	-1.0361	0.5311	-1.0719	0.2345	-1.1203	0.0581	-1.1673	0.0148	-1.2205	0.0026	-1.3333	0.0001	purinergic receptor P2X, ligand-gated ion channel, 5
ILMN_1764415	ZNF585A	199704	243	278	227	283	234	244	186	208	-1.0710	0.3323	1.0203	0.7691	-1.0409	0.5653	-1.1367	0.0787	-1.3048	0.0016	-1.3358	0.0006	zinc finger protein 585A
ILMN_1660723	RDH13	112724	254	245	238	201	234	207	195	182	-1.0691	0.3610	-1.2149	0.0168	-1.0859	0.2624	-1.1840	0.0333	-1.3045	0.0019	-1.3416	0.0009	retinol dehydrogenase 13 (all-trans/9-cis)
ILMN_2051867	PTCD2	79810	512	560	546	614	538	534	399	417	1.0666	0.2456	1.0964	0.1009	1.0520	0.3599	-1.0492	0.3779	-1.2817	0.0003	-1.3445	0.0000	pentatricopeptide repeat domain 2
ILMN_1656977	HIBCH	26275	1346	1382	1085	1139	1283	1230	932	1024	-1.2406	0.0087	-1.2135	0.0164	-1.0493	0.5243	-1.1237	0.1289	-1.4444	0.0001	-1.3501	0.0006	3-hydroxyisobutyryl-Coenzyme A hydrolase
ILMN_1790249	F8A1	8263	647	660	739	569	726	686	500	488	1.1421	0.1996	-1.1602	0.1639	1.1218	0.2651	1.0387	0.7094	-1.2942	0.0219	-1.3530	0.0092	coagulation factor VIII-associated (intronic transcript) 1
ILMN_2394264	UBTF	7343	198	171	159	138	174	152	133	126	-1.2452	0.0043	-1.2394	0.0074	-1.1395	0.0621	-1.1285	0.1005	-1.4888	0.0000	-1.3575	0.0005	upstream binding transcription factor, RNA polymerase I
ILMN_1759670	AMACR	23600	337	408	335	469	341	383	257	301	-1.0061	0.9478	1.1490	0.1499	1.0109	0.9184	-1.0664	0.5122	-1.3110	0.0153	-1.3577	0.0061	alpha-methylacyl-CoA racemase
ILMN_1670377	ZNF20	7568	387	464	396	455	351	409	309	341	1.0220	0.7426	-1.0184	0.7830	-1.1055	0.1543	-1.1352	0.0677	-1.2538	0.0037	-1.3608	0.0002	zinc finger protein 20
ILMN_2352131	ERBB2	2064	1090	1023	1173	1066	1145	1073	727	752	1.0763	0.3768	1.0414	0.6239	1.0508	0.5474	1.0489	0.5669	-1.4986	0.0001	-1.3615	0.0014	v-erb-b2 erythroblastic leukemia viral oncogene homolog 2, neuro/glioblastoma derived oncogene homolog (avian)
ILMN_2398378	PML	5371	150	151	139	120	129	130	114	111	-1.0840	0.2929	-1.2654	0.0065	-1.1685	0.0571	-1.1646	0.0572	-1.3150	0.0023	-1.3643	0.0008	promyelocytic leukemia
ILMN_2341952	MRPL35	51318	1851	1991	1958	1929	1864	1977	1371	1458	1.0579	0.4576	-1.0323	0.6705	1.0068	0.9311	-1.0074	0.9207	-1.3502	0.0007	-1.3661	0.0005	mitochondrial ribosomal protein L35
ILMN_1797372	C3orf58	205428	430	341	420	376	376	381	300	249	-1.0246	0.8105	1.1013	0.3281	-1.1442	0.1650	1.1166	0.2562	-1.4321	0.0013	-1.3714	0.0053	chromosome 3 open reading frame 58
ILMN_2087575	ZC3H4	23211	1484	1155	1193	981	1226	1002	1063	842	-1.2437	0.0085	-1.1773	0.0441	-1.2107	0.0185	-1.1521	0.0795	-1.3962	0.0003	-1.3718	0.0005	zinc finger CCCH-type containing 4
ILMN_1791792	C12orf5	57103	439	454	374	403	448	444	322	331	-1.1756	0.0383	-1.1267	0.1163	1.0196	0.7948	-1.0240	0.7555	-1.3640	0.0005	-1.3727	0.0004	chromosome 12 open reading frame 5
ILMN_1740500	TSPYL4	23270	355	264	281	233	279	235	202	192	-1.2606	0.0047	-1.1341	0.1189	-1.2727	0.0038	-1.1238	0.1440	-1.7568	0.0000	-1.3729	0.0008	TSPY-like 4
ILMN_1764320	C8orf58	541565	348	272	304	241	279	249	217	198	-1.1417	0.0534	-1.1281	0.0916	-1.2464	0.0031	-1.0921	0.2072	-1.5989	0.0000	-1.3736	0.0002	chromosome 8 open reading frame 58
ILMN_1743352	TBCC	6903	735	844	726	815	790	710	558	611	-1.0121	0.8814	-1.0347	0.6582	1.0750	0.3520	-1.1882	0.0354	-1.3173	0.0022	-1.3819	0.0005	tubulin folding cofactor C
ILMN_2309180	SMARCD3	6604	458	455	417	395	381	468	326	328	-1.0981	0.1564	-1.1497	0.0460	-1.2007	0.0105	1.0301	0.6471	-1.4054	0.0001	-1.3880	0.0001	SWI/SNF related, matrix associated, actin dependent regulator of chromatin, subfamily d, member 3
ILMN_2367172	AMACR	23600	233	271	216	268	235	236	163	193	-1.0794	0.1712	-1.0103	0.8311	1.0093	0.8616	-1.1502	0.0135	-1.4270	0.0000	-1.4061	0.0000	alpha-methylacyl-CoA racemase
ILMN_1737170	FLII	2314	803	695	693	546	645	559	492	494	-1.1594	0.0830	-1.2727	0.0090	-1.2450	0.0138	-1.2431	0.0162	-1.6332	0.0000	-1.4072	0.0006	flightless I homolog (Drosophila)
ILMN_1753016	MRPL35	51318	367	327	333	330	283	312	233	232	-1.1032	0.2907	1.0067	0.9430	-1.2960	0.0120	-1.0501	0.6072	-1.5740	0.0001	-1.4085	0.0021	mitochondrial ribosomal protein L35
ILMN_2329834	RBM12	10137	1188	1192	1164	1083	1062	1120	861	844	-1.0209	0.7902	-1.1010	0.2318	-1.1194	0.1620	-1.0648	0.4286	-1.3796	0.0006	-1.4132	0.0003	RNA binding motif protein 12
ILMN_1741350	CEP70	80321	324	327	278	286	336	297	226	230	-1.1675	0.0196	-1.1466	0.0351	1.0364	0.5500	-1.1009	0.1268	-1.4328	0.0000	-1.4243	0.0000	centrosomal protein 70kDa
ILMN_2288232	PHKB	5257	486	446	436	356	421	352	344	313	-1.1131	0.1992	-1.2520	0.0123	-1.1542	0.0875	-1.2658	0.0092	-1.4122	0.0004	-1.4264	0.0004	phosphorylase kinase, beta
ILMN_1735199	CIAPIN1	57019	531	483	483	432	466	448	367	339	-1.0993	0.1946	-1.1188	0.1327	-1.1390	0.0816	-1.0794	0.2988	-1.4474	0.0001	-1.4275	0.0001	cytokine induced apoptosis inhibitor 1
ILMN_1667577	LCMT2	9836	841	848	764	651	936	827	562	591	-1.1019	0.3778	-1.3033	0.0233	1.1123	0.3220	-1.0246	0.8264	-1.4962	0.0015	-1.4354	0.0033	leucine carboxyl methyltransferase 2
ILMN_2384536	PECI	10455	1836	1706	1513	1472	1532	1417	1174	1185	-1.2134	0.0166	-1.1591	0.0604	-1.1980	0.0240	-1.2039	0.0215	-1.5632	0.0000	-1.4392	0.0001	peroxisomal D3,D2-enoyl-CoA isomerase
ILMN_1788283	COTL1	23406	201	198	162	148	165	163	145	137	-1.2403	0.0295	-1.3418	0.0048	-1.2192	0.0396	-1.2202	0.0411	-1.3916	0.0020	-1.4499	0.0009	coactosin-like 1 (Dictyostelium)
ILMN_1687403	MRPL40	64976	2138	2267	1774	1838	1963	1927	1468	1561	-1.2052	0.0474	-1.2333	0.0270	-1.0889	0.3427	-1.1767	0.0781	-1.4566	0.0004	-1.4520	0.0004	mitochondrial ribosomal protein L40
ILMN_1734608	ZNF77	58492	252	270	276	261	241	230	196	185	1.0963	0.1932	-1.0325	0.6588	-1.0444	0.5488	-1.1749	0.0354	-1.2875	0.0028	-1.4595	0.0001	zinc finger protein 77
ILMN_2384544	ADAM15	8751	348	306	353	257	358	282	264	209	1.0127	0.8759	-1.1916	0.0347	1.0282	0.7157	-1.0840	0.3020	-1.3193	0.0015	-1.4606	0.0001	ADAM metallopeptidase domain 15
ILMN_1758827	RTN4IP1	84816	411	430	382	425	390	397	267	294	-1.0740	0.3161	-1.0129	0.8532	-1.0533	0.4777	-1.0841	0.2523	-1.5366	0.0000	-1.4606	0.0000	reticulon 4 interacting protein 1
ILMN_1730888	ZNF680	340252	160	178	149	159	148	147	119	121	-1.0728	0.2982	-1.1168	0.0965	-1.0802	0.2575	-1.2072	0.0087	-1.3417	0.0004	-1.4624	0.0000	zinc finger protein 680
ILMN_1685978	ATPIF1	93974	302	306	256	243	240	291	195	209	-1.1813	0.0639	-1.2594	0.0149	-1.2564	0.0158	-1.0531	0.5424	-1.5474	0.0001	-1.4677	0.0004	ATPase inhibitory factor 1
ILMN_1739521	NLGN1	22871	493	388	451	401	503	430	371	264	-1.0944	0.1256	1.0333	0.5837	1.0194	0.7445	1.1101	0.0894	-1.3298	0.0001	-1.4704	0.0000	neuroligin 1
ILMN_1769158	ISOC2	79763	598	578	577	534	526	459	416	391	-1.0358	0.6508	-1.0814	0.3130	-1.1366	0.1046	-1.2574	0.0075	-1.4378	0.0001	-1.4762	0.0001	isochorismatase domain containing 2

ILMN_1655748	ZNF323	64288	210	223	185	232	173	185	156	151	-1.1326	0.1075	1.0429	0.5606	-1.2105	0.0192	-1.2018	0.0210	-1.3473	0.0010	-1.4795	0.0001	zinc finger protein 323
ILMN_1726755	COPS4	51138	1913	2036	1664	1820	1759	1780	1329	1376	-1.1497	0.0192	-1.1188	0.0523	-1.0879	0.1381	-1.1441	0.0226	-1.4392	0.0000	-1.4797	0.0000	COP9 constitutive photomorphogenic homolog subunit 4 (Arabidopsis)
ILMN_2217329	IAH1	285148	3858	3482	3511	2893	3930	3228	2407	2347	-1.0990	0.2884	-1.2036	0.0454	1.0187	0.8330	-1.0785	0.3938	-1.6025	0.0000	-1.4836	0.0002	isoamyl acetate-hydrolyzing esterase 1 homolog (S. cerevisiae)
ILMN_2119793	ANKRD34A	284615	297	265	262	218	252	228	208	178	-1.1370	0.0842	-1.2132	0.0162	-1.1798	0.0307	-1.1626	0.0521	-1.4284	0.0001	-1.4853	0.0001	ankyrin repeat domain 34A
ILMN_1727532	OLFML3	56944	229	243	223	225	189	207	171	163	-1.0265	0.6916	-1.0781	0.2514	-1.2124	0.0082	-1.1713	0.0218	-1.3417	0.0003	-1.4916	0.0000	olfactomedin-like 3
ILMN_1773493	TIMM23	10431	2677	2594	2074	2031	2196	2084	1650	1728	-1.2908	0.0146	-1.2771	0.0184	-1.2189	0.0505	-1.2449	0.0324	-1.6219	0.0001	-1.5010	0.0004	translocase of inner mitochondrial membrane 23 homolog (yeast)
ILMN_2330861	SMC4	10051	2610	2230	2082	2043	2573	2248	1855	1485	-1.2534	0.0278	-1.0916	0.3692	-1.0144	0.8816	1.0083	0.9282	-1.4074	0.0020	-1.5018	0.0004	structural maintenance of chromosomes 4
ILMN_1659727	CDAN1	146059	306	282	305	257	350	291	213	187	-1.0011	0.9922	-1.0983	0.2083	1.1446	0.0607	1.0322	0.6564	-1.4375	0.0001	-1.5079	0.0000	congenital dyserythropoietic anemia, type I
ILMN_1661799	HIGD1A	25994	3982	4724	3914	4496	3491	3682	2934	3131	-1.0174	0.8426	-1.0506	0.5762	-1.1408	0.1472	-1.2830	0.0099	-1.3574	0.0024	-1.5085	0.0001	HIG1 hypoxia inducible domain family, member 1A
ILMN_1733110	RASSF7	8045	2182	2151	2544	1825	2213	1914	1395	1426	1.1660	0.1005	-1.1787	0.0803	1.0142	0.8838	-1.1241	0.2038	-1.5641	0.0001	-1.5088	0.0002	Ras association (RalGDS/AF-6) domain family (N-terminal) member 7
ILMN_2325978	HDGF2	84717	873	744	650	506	722	679	525	483	-1.3425	0.0219	-1.4710	0.0052	-1.2089	0.1208	-1.0955	0.4627	-1.6634	0.0004	-1.5386	0.0022	hepatoma-derived growth factor-related protein 2
ILMN_1748438	POLR2G	5436	3347	3888	3667	3742	3395	3413	2871	2503	1.0955	0.1294	-1.0391	0.5118	1.0144	0.8066	-1.1390	0.0351	-1.1661	0.0154	-1.5530	0.0000	polymerase (RNA) II (DNA directed) polypeptide G
ILMN_2300186	DYNLL1	8655	10082	10949	9884	10042	9365	9594	7096	7007	-1.0200	0.8146	-1.0903	0.3141	-1.0766	0.3885	-1.1413	0.1304	-1.4207	0.0005	-1.5625	0.0000	dynein, light chain, LC8-type 1
ILMN_2054392	PPIL1	51645	915	803	885	757	851	717	495	513	-1.0338	0.7703	-1.0607	0.6010	-1.0751	0.5169	-1.1205	0.3214	-1.8477	0.0000	-1.5663	0.0009	peptidylprolyl isomerase (cyclophilin)-like 1
ILMN_2411731	HPS5	11234	1383	1024	1198	865	1323	919	797	651	-1.1543	0.0204	-1.1837	0.0093	-1.0449	0.4475	-1.1146	0.0790	-1.7339	0.0000	-1.5727	0.0000	Hermansky-Pudlak syndrome 5
ILMN_1781996	NUDT16	131870	293	340	303	317	278	290	205	216	1.0344	0.5674	-1.0730	0.2254	-1.0527	0.4039	-1.1711	0.0130	-1.4288	0.0000	-1.5749	0.0000	nudix (nucleoside diphosphate linked moiety X)-type motif 16
ILMN_1785170	ARMCX2	9823	678	750	633	715	542	663	443	468	-1.0696	0.3544	-1.0499	0.4914	-1.2490	0.0058	-1.1320	0.0937	-1.5308	0.0000	-1.6048	0.0000	armadillo repeat containing, X-linked 2
ILMN_1665205	ZNF260	339324	555	597	513	528	501	463	408	371	-1.0819	0.3478	-1.1296	0.1465	-1.1064	0.2275	-1.2891	0.0053	-1.3607	0.0014	-1.6078	0.0000	zinc finger protein 260
ILMN_2043918	DLEU1	10301	1241	1447	983	1235	1081	1138	751	892	-1.2628	0.0317	-1.1714	0.1265	-1.1485	0.1823	-1.2713	0.0256	-1.6538	0.0001	-1.6216	0.0001	deleted in lymphocytic leukemia 1 (non-protein coding)
ILMN_1730685	MRPL16	54948	284	292	257	281	308	253	211	180	-1.1047	0.1336	-1.0392	0.5395	1.0858	0.2000	-1.1528	0.0362	-1.3492	0.0002	-1.6246	0.0000	mitochondrial ribosomal protein L16
ILMN_1663132	ADCK2	90956	797	719	967	648	824	657	619	440	1.2138	0.0532	-1.1084	0.3016	1.0347	0.7233	-1.0939	0.3669	-1.2869	0.0173	-1.6318	0.0001	aarF domain containing kinase 2
ILMN_1784946	ORC3L	23595	605	559	462	440	477	494	382	343	-1.3086	0.0051	-1.2724	0.0111	-1.2680	0.0116	-1.1325	0.1575	-1.5840	0.0000	-1.6320	0.0000	origin recognition complex, subunit 3-like (yeast)
ILMN_2246563	COL8A1	1295	598	357	506	262	525	378	318	219	-1.1823	0.1127	-1.3616	0.0153	-1.1409	0.2048	1.0579	0.6066	-1.8818	0.0000	-1.6347	0.0005	collagen, type VIII, alpha 1
ILMN_1740900	BMP4	652	4076	2163	3999	2608	4055	3436	1602	1320	-1.0191	0.8463	1.2054	0.0709	-1.0051	0.9595	1.5885	0.0001	-2.5439	0.0000	-1.6394	0.0001	bone morphogenetic protein 4
ILMN_1768077	TCTN3	26123	446	409	457	378	409	352	300	249	1.0245	0.7545	-1.0815	0.3211	-1.0907	0.2566	-1.1603	0.0703	-1.4841	0.0001	-1.6450	0.0000	tectonic family member 3
ILMN_1665538	SKP2	6502	1311	871	1252	810	1378	1014	709	527	-1.0472	0.5795	-1.0747	0.4063	1.0518	0.5445	1.1639	0.0849	-1.8498	0.0000	-1.6511	0.0000	S-phase kinase-associated protein 2 (p45)
ILMN_1681456	FOXL2	668	320	265	250	199	370	248	186	158	-1.2804	0.0659	-1.3327	0.0423	1.1562	0.2333	-1.0688	0.6302	-1.7238	0.0005	-1.6735	0.0014	forkhead box L2
ILMN_2343278	PPAP2A	8611	1334	1836	1249	1647	1149	1706	810	1092	-1.0678	0.3828	-1.1146	0.1463	-1.1607	0.0569	-1.0761	0.3193	-1.6473	0.0000	-1.6810	0.0000	phosphatidic acid phosphatase type 2A
ILMN_2209027	RPS26	6231	11302	10491	7995	7563	8953	7057	6191	6057	-1.4136	0.0110	-1.3871	0.0154	-1.2623	0.0733	-1.4867	0.0045	-1.8256	0.0001	-1.7321	0.0003	ribosomal protein S26
ILMN_1753627	DOLK	22845	1168	1414	1115	1127	1065	1120	792	813	-1.0471	0.6347	-1.2550	0.0222	-1.0964	0.3303	-1.2634	0.0191	-1.4751	0.0005	-1.7391	0.0000	dolichol kinase
ILMN_1657446	C1orf57	84284	1603	1369	1243	1133	1302	1058	937	770	-1.2892	0.0154	-1.2081	0.0638	-1.2312	0.0422	-1.2941	0.0153	-1.7105	0.0000	-1.7776	0.0000	chromosome 1 open reading frame 57
ILMN_1651826	BASP1	10409	16808	15675	14078	11146	14258	12715	9929	8426	-1.1939	0.1318	-1.4064	0.0069	-1.1789	0.1601	-1.2328	0.0787	-1.6929	0.0002	-1.8603	0.0000	brain abundant, membrane attached signal protein 1
ILMN_1705750	TGM2	7052	2169	902	2793	959	2126	1135	972	476	1.2876	0.0321	1.0634	0.6049	-1.0203	0.8578	1.2579	0.0623	-2.2307	0.0000	-1.8936	0.0000	transglutaminase 2 (C polypeptide, protein-glutamine-gamma-glutamyltransferase)
ILMN_2067852	SLC30A1	7779	454	518	369	453	383	511	314	271	-1.2294	0.0118	-1.1414	0.0810	-1.1849	0.0335	-1.0127	0.8591	-1.4463	0.0001	-1.9110	0.0000	solute carrier family 30 (zinc transporter), member 1
ILMN_1765001	NAT6	24142	620	603	511	393	473	413	363	315	-1.2144	0.1590	-1.5356	0.0040	-1.3113	0.0491	-1.4608	0.0095	-1.7099	0.0007	-1.9164	0.0001	N-acetyltransferase 6 (GCN5-related)
ILMN_1653711	FZD2	2535	4273	3907	4246	3363	4889	4052	2635	1882	-1.0064	0.9383	-1.1618	0.0887	1.1442	0.1214	1.0370	0.6688	-1.6215	0.0000	-2.0759	0.0000	frizzled homolog 2 (Drosophila)
ILMN_2224833	ADIPOR2	79602	2117	2577	2014	2191	2073	2471	1096	1155	-1.0513	0.4197	-1.1760	0.0143	-1.0217	0.7273	-1.0428	0.4944	-1.9322	0.0000	-2.2310	0.0000	adiponectin receptor 2
ILMN_1779147	ENC1	8507	3150	3535	3092	3062	3399	4287	1572	1258	-1.0186	0.8172	-1.1547	0.0796	1.0790	0.3398	1.2128	0.0219	-2.0043	0.0000	-2.8092	0.0000	ectodermal-neural cortex (with BTB-like domain)
ILMN_2325347	B3GALNT1	8706	624	1006	445	680	368	706	208	257	-1.4010	0.0000	-1.4803	0.0000	-1.6969	0.0000	-1.4251	0.0000	-3.0003	0.0000	-3.9193	0.0000	beta-1,3-N-acetylgalactosaminyltransferase 1 (globoside blood group)
ILMN_1736670	PPP1R3C	5507	2692	2785	2182	1967	2133	2411	524	647	-1.2340	0.0005	-1.4156	0.0000	-1.2622	0.0002	-1.1549	0.0095	-5.1385	0.0000	-4.3053	0.0000	protein phosphatase 1, regulatory (inhibitor) subunit 3C
ILMN_1718766	MT1F	4494	3469	1939	322	224	2541	1539	896	447	-10.7816	0.0000	-8.6412	0.0000	-1.3654	0.0000	-1.2603	0.0007	-3.8737	0.0000	-4.3354	0.0000	metallothionein 1F
ILMN_2075065	FADS2	9415	566	631	358	408	259	392	128	132	-1.5810	0.0008	-1.5471	0.0010	-2.1797	0.0000	-1.6118	0.0005	-4.4219	0.0000	-4.7727	0.0000	fatty acid desaturase 2
ILMN_2371379	ACLY	47	10253	16055	4343	6508	5086	8250	2104	2800	-2.3607	0.0000	-2.4670	0.0000	-2.0157	0.0000	-1.9461	0.0000	-4.8723	0.0000	-5.7348	0.0000	ATP citrate lyase
ILMN_1670134	FADS1	3992	4279	5813	2122	2832	1865	3681	774	906	-2.0164	0.0000	-2.0526	0.0000	-2.2947	0.0000	-1.5791	0.0000	-5.5257	0.0000	-6.4188	0.0000	fatty acid desaturase 1
ILMN_1784871	FASN	2194	17814	27631	13089	22418	6580	15682	2818	3970	-1.3610	0.0003	-1.2325	0.0073	-2.7073	0.0000	-1.7620	0.0000	-6.3214	0.0000	-6.9601	0.0000	fatty acid synthase
ILMN_1813671	SLC25A1	6576	3389	4170	2208	1563	1400	1497	633	560	-1.5348	0.0001	-2.6674	0.0000	-2.4213	0.0000	-2.7858	0.0000	-5.3533	0.0000	-7.4448	0.0000	solute carrier family 25 (mitochondrial carrier; citrate transporter), member 1
ILMN_1671554	LPIN1	23175	11024	10909	5631	5957	3985	4863	1790	1325	-1.9579	0.0000	-1.8314	0.0000	-2.7664	0.0000	-2.2433	0.0000	-6.1591	0.0000	-8.2362	0.0000	lipin 1
ILMN_1686989	INSIG1	3638	1374	1646	782	860	308	552	143	147	-1.7574	0.0000	-1.9128	0.0000	-4.4669	0.0000	-2.9795	0.0000	-9.5965	0.0000	-11.1646	0.0000	insulin induced gene 1
ILMN_2053415	LDLR	3949	9315	12019	11082	14443	4462	9734	708	1073	1.1896	0.0185	1.2016	0.0132	-2.0875	0.0000	-1.2347	0.0055	-13.1621	0.0000	-11.2003	0.0000	low density lipoprotein receptor
ILMN_1715024	LSS	4047	6100	7072	4503	3717	1201	2281	566	565	-1.3546	0.0001	-1.9028	0.0000	-5.0783	0.0000	-3.1010	0.0000	-10.7749	0.0000	-12.5164	0.0000	lanosterol synthase (2,3-oxidosqualene-lanosterol cyclase)
ILMN_2165867	DHCR7	1717	4089	5079	4140	4072	862	1732	292	338	1.0123	0.8691	-1.2474	0.0065	-4.7449	0.0000	-2.9332	0.0000	-14.0114	0.0000	-15.0253	0.0000	7-dehydrocholesterol reductase
ILMN_1689329	SCD	6319	25369	27982	17519	20001	10777	15634	1498	1740	-1.4481	0.0001	-1.3990	0.0003	-2.3540	0.0000	-1.7898	0.0000	-16.9295	0.0000	-16.0804	0.0000	stearoyl-CoA desaturase (delta-9-desaturase)
