# Supplementary figures and images for: Sterol regulatory element binding protein-dependent regulation of lipid synthesis supports cell survival and tumor growth
Source: Cancer Metab. 2013 Jan 23;1:3. doi: 10.1186/2049-3002-1-3 (PMC3835903; doi:10.1186/2049-3002-1-3)

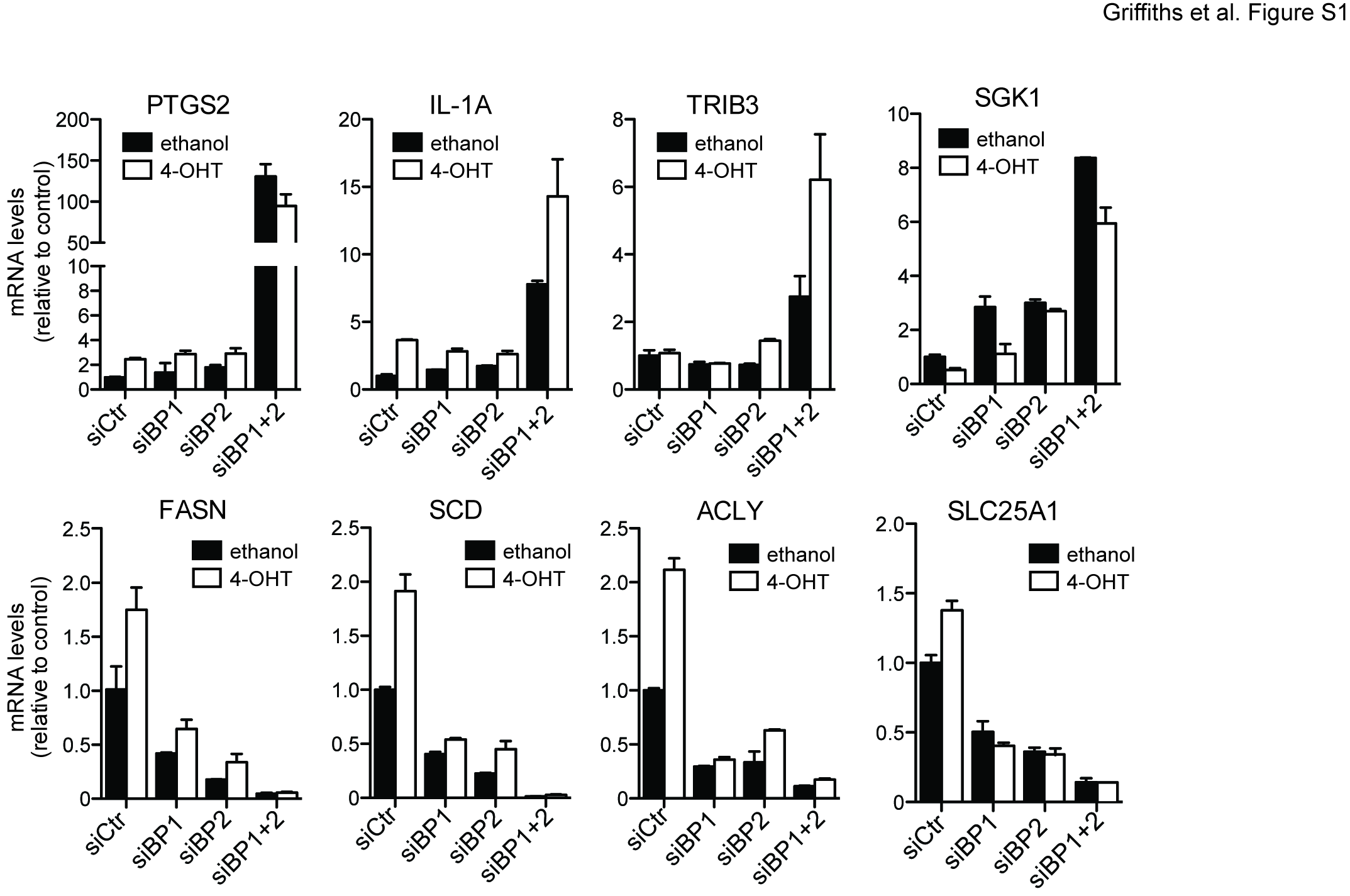

Supplement: Additional file 3 — Figure S1. Validation of microarray experiment. RNA from cells after single or combined silencing of SREBP1 and SREBP2 treated with 100 nM 4-OHT or solvent (ethanol) for 24 hours in medium containing 1% lipoprotein deficient serum (LPDS) was used to determine the expression of selected upregulated and downregulated genes. Graph shows mean ± SD of two independent experiments. [file 2049-3002-1-3-S3.tiff]

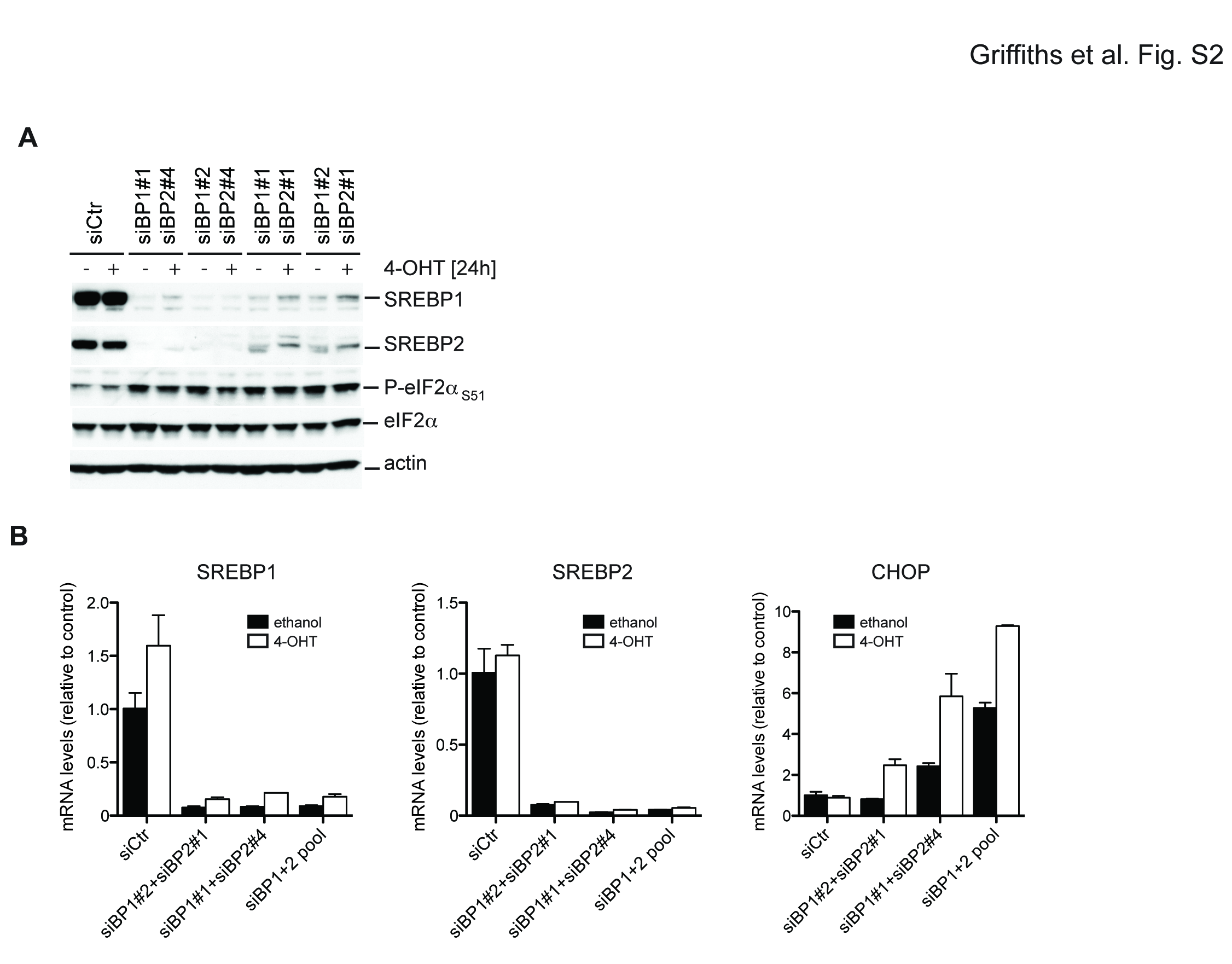

Supplement: Additional file 4 — Figure S2. Silencing of SREBP1 and SREBP2 using different siRNA sequences induces eIF2α phosphorylation and CHOP expression. (A) RPE-myrAkt-ER cells were transfected with different combinations of siRNA oligonucleotides specific for SREBP1 (siBP1#1 or siBP1#2) or SREBP2 (siBP2#1 or siBP2#4) or pools of four oligonucleotides targeting either gene (siBP1 + 2 pool). At 72 hours post-transfection, cells were placed into medium supplemented with 1% LPDS and treated with 100 nM 4-OHT or solvent (ethanol) for 24 hours. Lysates were analyzed for expression of SREBP1, SREBP2, phospho eIF2α (serine 51) and total eIF2α by immunoblotting. Actin was used as a loading control. (B) RNA from cells treated in parallel to A was used to determine expression of SREBP1, SREBP2 and CHOP by qRT-PCR. Graphs show mean ± SD of two independent experiments. [file 2049-3002-1-3-S4.tiff]

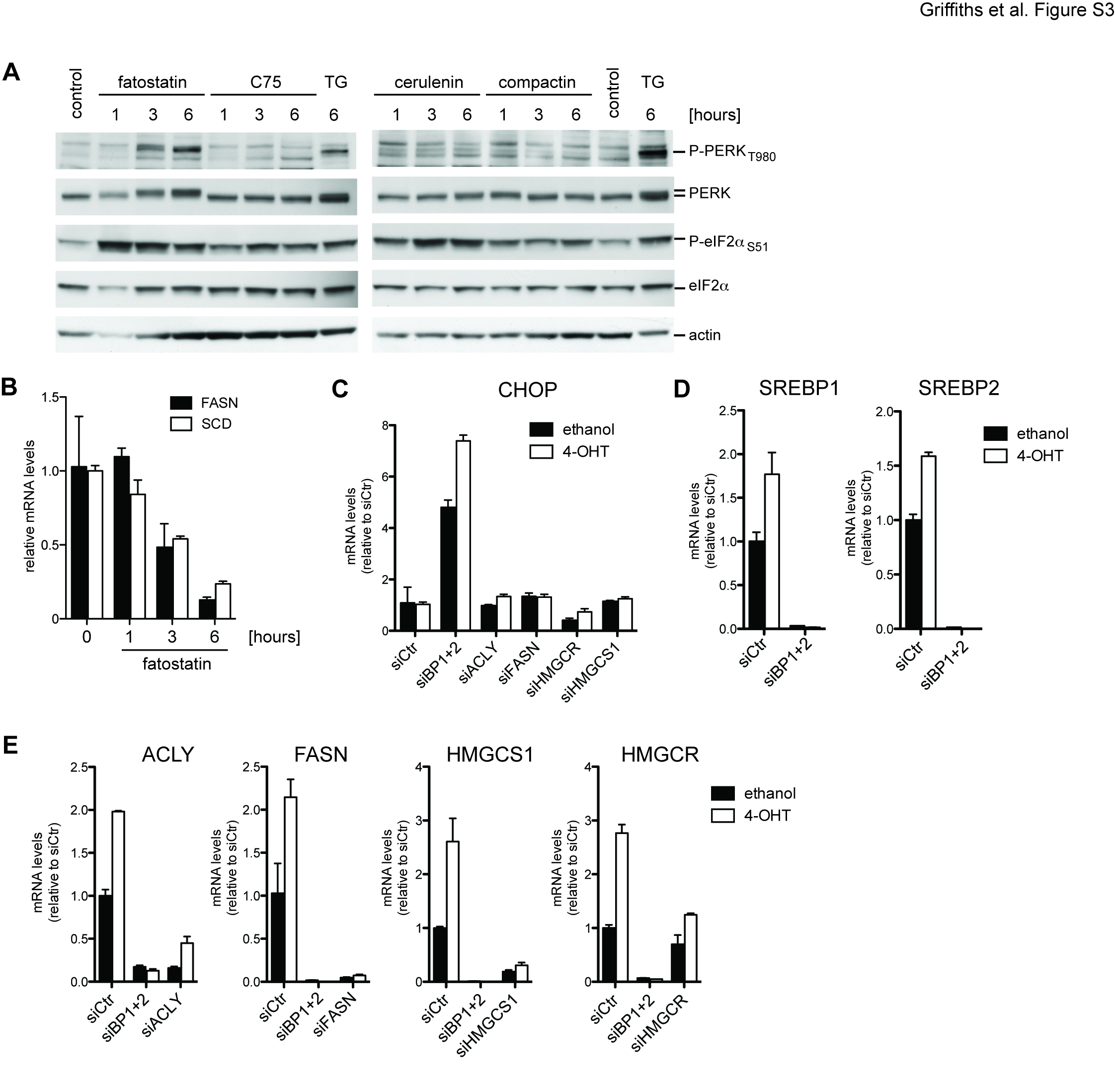

Supplement: Additional file 5 — Figure S3. Inhibition of fatty acid or cholesterol biosynthesis is not sufficient to induce ER-stress. (A) Parental RPE-hTERT cells were placed in medium containing 1% LPDS for 24 hours and treated with 20 μM fatostatin, 45 μM C75, 40 μM cerulenin or 10 μM compactin (mevastatin) for the final 1, 3 or 6 hours or with 50 nM thapsigargin (TG) for the last 6 hours. Whole cell lysates were analyzed for expression and phosphorylation of PERK and eIF2α. (B) Expression of the SREBP target genes FASN and SCD in cells treated with 20 μM fatostatin for 1, 3 or 6 hours in medium containing 1% LPDS. Graph shows mean ± SD of two independent experiments. (C) RPE-myrAkt-ER cells were transfected with siRNA oligonucleotides targeting the indicated genes. At 72 hours post-transfection, cells were placed into medium supplemented with 1% LPDS and treated with 100 nM 4-OHT or solvent for 24 hours. Expression of CHOP was determined by qRT-PCR. Graph shows mean ± SD of two independent experiments. (D) Efficiency of downregulation of SREBP1 and SREBP2 after siRNA transfection was determined by qRT-PCR. Graphs show mean ± SD of two independent experiments. (E) Efficiency of downregulation of each target gene following SREBP depletion or gene-specific siRNA transfection was determined by qRT-PCR. Graphs show mean ± SD of two independent experiments. [file 2049-3002-1-3-S5.tiff]

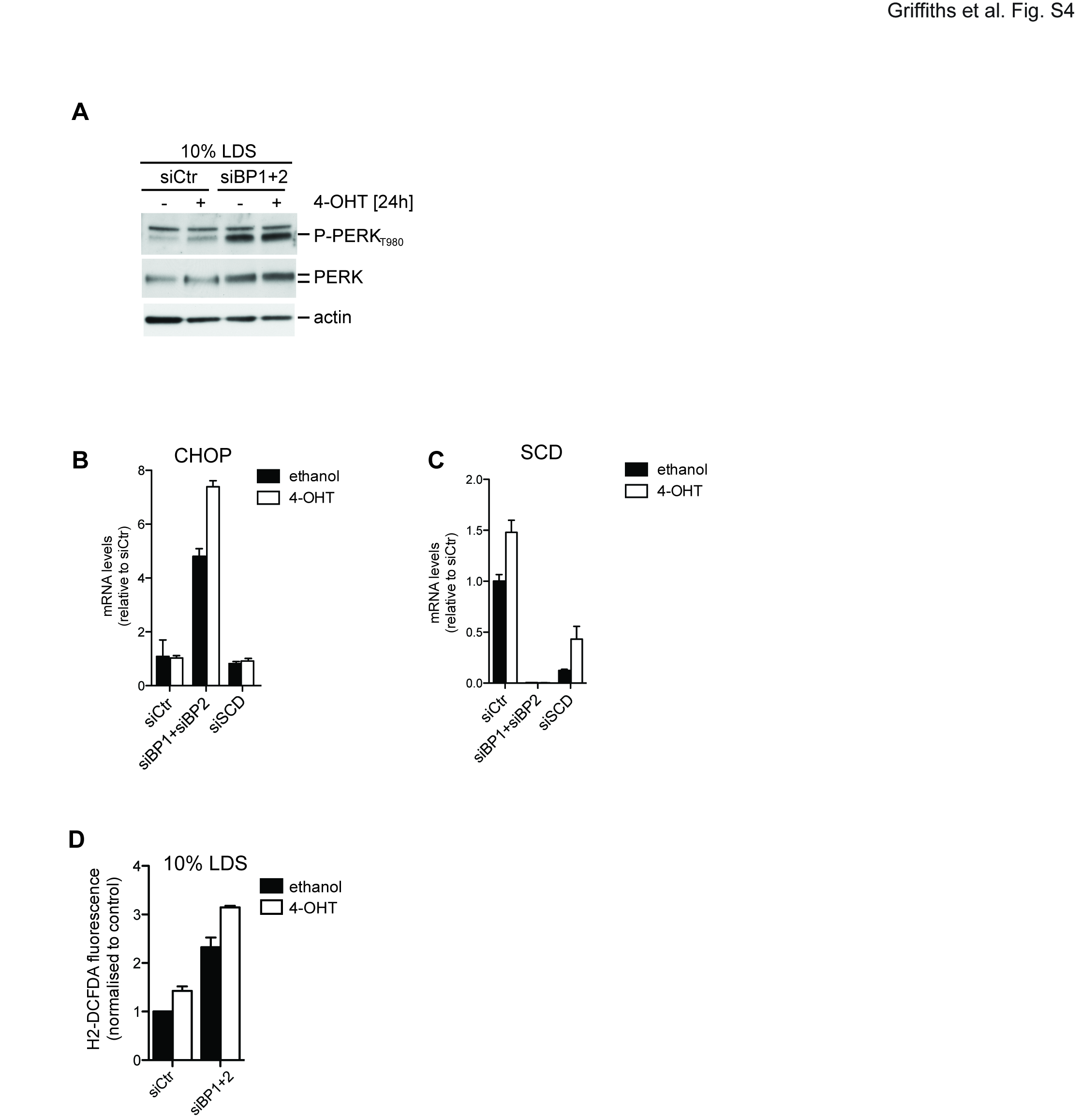

Supplement: Additional file 9 — Figure S4. Silencing of SREBP1 and SREBP2 induces PERK phosphorylation and ROS in medium supplemented with lipid depleted serum. (A) Cells depleted of SREBP1 and SREBP2 were placed in medium supplemented with 10% lipid depleted serum (LDS), treated with 100 nM 4-OHT or solvent (ethanol) for 24 hours. Lysates were analyzed for phosphorylation of Perk. Actin is used as loading control. (B) RPE-myrAkt-ER cells were transfected with siRNA oligonucleotides targeting SREBP1 and SREBP2 (siBP1+2) or SCD (siSCD). At 72 hours post-transfection, cells were placed into medium supplemented with 1% LPDS and treated with 100 nM 4-OHT or solvent for 24 hours. Expression of CHOP was determined by qRT-PCR. Graph shows mean ± range of two independent experiments. (C) Efficiency of downregulation of SCD after siRNA transfection was determined by qRT-PCR. Graphs show mean ± range of two independent experiments. (D) Cells treated as in A were used to determine ROS levels by CM-H2DCFDA staining and FACS analysis. Graph shows mean ± range of two independent experiments. [file 2049-3002-1-3-S9.tiff]

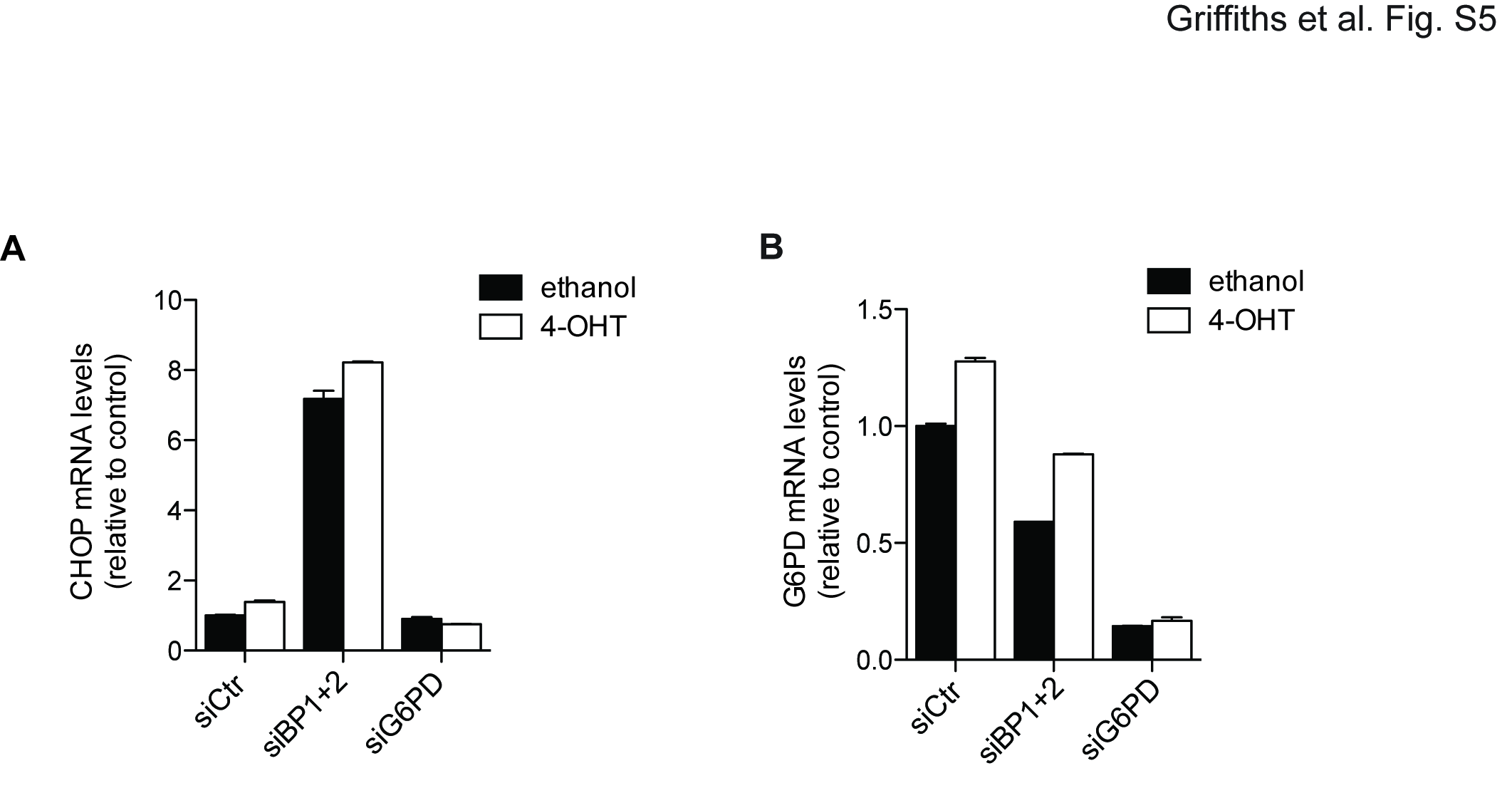

Supplement: Additional file 10 — Figure S5. Silencing of glucose-6-phosphate dehydrogenase does not induce ER-stress. RPE-myrAkt-ER cells were transfected with siRNA oligonucleotides targeting SREBP1 and SREBP2 or glucose-6-phosphate dehydrogenase (G6PD). At 72 hours post-transfection, cells were placed into medium supplemented with 1% LPDS and treated with 100 nM 4-OHT or solvent (ethanol) for 24 hours. Graphs show mean ± SD of two independent experiments. (A) RNA was used to determine expression of CHOP by qRT-PCR. (B) Efficient depletion of G6PD was determined by qRT-PCR. [file 2049-3002-1-3-S10.tiff]

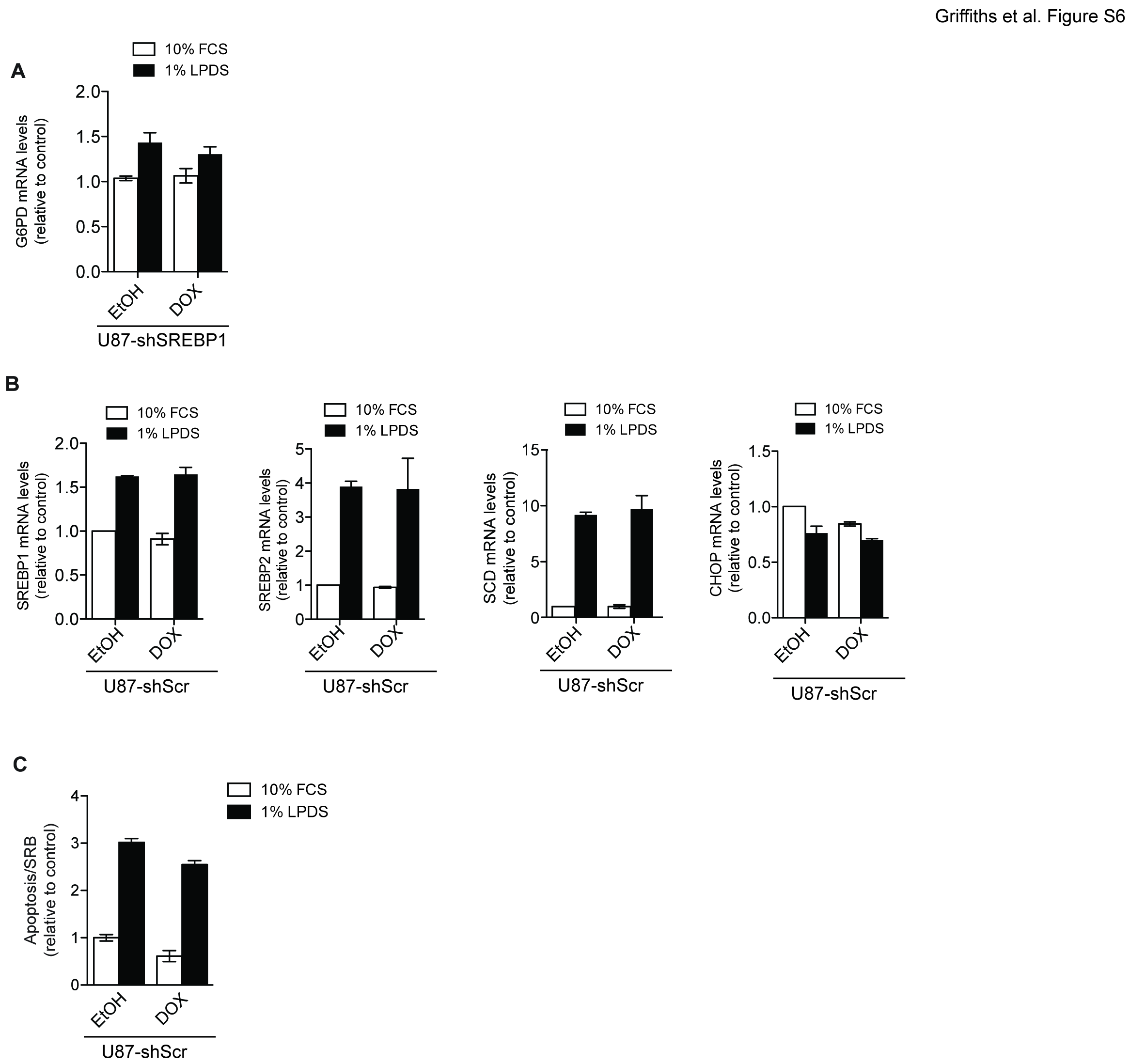

Supplement: Additional file 11 — Figure S6. Expression of G6PD in U87 cells after silencing of SREBP1 and controls using a non-targeting shRNA sequence. (A) U87 cells expressing inducible shRNA targeting SREBP1 (U87-shSREBP1) were treated with 1 μg/ml doxycycline or solvent for 48 hours and then placed in medium containing either 10% FCS or 1% LPDS for a further 24 hours. Expression of G6PD mRNA was determined by qPCR. Graphs show mean ± SEM of three independent experiments. (B) U87 cells expressing a scrambled shRNA sequence (U87-shScr) were treated as in A. Expression of SREBP1, SREBP2, SCD and CHOP was determined by qRT-PCR. Graphs show mean ± SD of two independent experiments. (C) Induction of apoptosis (caspase 3/7 activity) was determined in U87 cells expressing a scrambled shRNA sequence. Cells were treated with 1 μg/ml doxycycline or solvent for 48 hours before being placed in medium containing either 10% FCS or 1% LPDS for a further 48 hours. Graph shows mean ± SEM of three independent experiments. *P < 0.05; n.s. = non significant. [file 2049-3002-1-3-S11.tiff]
