# Supplementary material for: Sterol regulatory element binding protein-dependent regulation of lipid synthesis supports cell survival and tumor growth
Source: Cancer Metab. 2013 Jan 23;1:3. doi: 10.1186/2049-3002-1-3 (PMC3835903; doi:10.1186/2049-3002-1-3)
Supplement: Additional file 6 — Table S2. SREBP depletion causes marked changes in cellular lipid composition. Lipid concentrations in RPE-myrAkt-ER were analyzed in cells following silencing of SREBP1 or SREBP2 or after combined ablation of both genes. Cells were placed in medium supplemented with 1% LPDS and treated with 100 nM 4-OHT or solvent (ethanol) for 24 hours. Lipid concentrations were determined by mass spectrometry and normalized to protein concentration. The two values represent biologically independent experiments. [file 2049-3002-1-3-S6.pdf]

Table S2: Lipid concentrations in RPE-myrAkt-ER cells after depletion of SREBP

	**concentration [ng/µg protein]**															
**lipid species**	**siControl ethanol**		**siControl 4-OHT**		**siBP1 ethanol**		**siBP1 4-OHT**		**siBP2 ethanol**		**siBP2 4-OHT**		**siBP1+2 ethanol**		**siBP1+2 4-OHT**	
free fatty acid	6568.03	4006.54	6746.66	4523.21	4662.29	2305.41	3006.82	2695.09	2874.88	2275.89	2308.89	1718.84	2386.6268	2010.0922	1812.4859	1694.9776
monoacylglycerol	193.85	167.27	205.31	232.02	274.20	164.48	293.76	212.65	55.06	87.87	82.46	88.26	99.594076	106.63392	71.822883	90.289657
diacylglycerol	571.35	506.43	1302.84	1000.64	445.03	333.88	524.36	522.87	290.40	343.27	507.13	364.79	336.78577	334.34706	436.92953	407.23694
triacylglycerol	243.05	239.63	483.70	360.59	509.83	279.46	446.95	424.46	242.52	225.01	285.73	265.84	178.60037	181.47083	212.87762	196.07989
cholesterol ester	855.27	569.00	970.47	680.43	760.96	433.42	771.94	626.53	541.19	501.96	620.56	534.80	442.88071	319.41683	449.75205	443.22113
cholesterol	29584.59	20783.97	24026.84	18114.93	26266.81	21594.37	16815.79	15704.74	16624.64	18018.18	15471.10	15883.00	10450.134	9460.9724	9170.5695	8858.1005
phosphatidic acid	179.66	105.71	248.95	177.86	223.37	197.04	276.91	493.98	356.70	348.53	301.22	364.20	648.50719	752.15065	814.13215	735.31712
phosphatidylglycerol	873.27	634.95	910.68	412.88	458.85	348.84	395.42	388.27	276.14	373.77	453.88	428.17	375.18371	347.98277	376.56588	314.93667
cardiolipin	6187.19	4635.35	6677.48	4723.24	3838.33	3341.03	5207.79	3531.35	3800.77	4726.98	5002.52	5852.68	5551.5666	5055.313	4760.4459	5006.4511
phosphatidylcholine	7208.45	4574.36	10910.07	5987.31	6481.41	3285.85	7713.37	6189.34	5575.77	6273.12	7901.92	5353.55	4742.2376	6944.9916	6375.1895	5495.8499
phosphatidylethanolamine	9620.47	4693.50	9543.06	5209.75	5615.51	4930.90	5239.92	3855.24	3923.26	4837.97	4649.72	5473.94	5640.6586	4565.9241	4762.0111	4582.0495
phosphatidylinositol	20363.66	14609.31	23586.253	19419.784	16890.514	14153.306	19207.539	19705.052	12252.371	13787.842	15889.845	15875.753	11632.56	10590.985	11586.317	11066.676
phosphatidylserine	7242.91	5902.82	9617.71	6182.72	3681.79	4177.04	4030.56	3149.76	2735.73	3249.21	3744.92	3373.37	3245.5793	3096.3237	3337.3884	3467.0058
lysophosphatidylcholine	305.46	226.84	202.85	215.18	392.86	263.36	245.57	221.61	179.76	237.48	116.27	118.02	196.96799	175.72466	162.88775	154.30365
sphingosine	4.47	2.78	4.06	2.75	1.63	1.39	1.57	1.82	3.94	4.08	2.90	3.10	1.1684402	1.0116043	1.9613448	2.0205162
ceramide	316.26	226.59	318.26	235.94	246.96	158.80	191.54	178.92	193.95	194.54	133.69	130.30	474.931	389.17584	128.74613	138.15863
sphingomyelin	7221.00	5589.39	9973.76	7433.29	6665.03	4450.49	6212.00	5818.92	4514.07	4855.71	5473.55	5539.62	4396.9653	4004.3542	4433.9032	4237.6034
**total lipids**	**97538.95**	**67474.46**	**105728.94**	**74912.54**	**77415.39**	**60419.06**	**70581.81**	**63720.60**	**54441.14**	**60341.43**	**62946.31**	**61368.21**	**50800.948**	**48336.871**	**48893.985**	**46890.278**
