# Supplementary material for: Sterol regulatory element binding protein-dependent regulation of lipid synthesis supports cell survival and tumor growth
Source: Cancer Metab. 2013 Jan 23;1:3. doi: 10.1186/2049-3002-1-3 (PMC3835903; doi:10.1186/2049-3002-1-3)
Supplement: Additional file 7 — Table S3. SREBP depletion causes a shift from unsaturated to saturated lipid species. Composition of the major lipid species was analyzed in RPE-myrAkt-ER cells after silencing of SREBP1 or SREBP2 or after combined ablation of both genes. Cells were placed in medium supplemented with 1% LPDS and treated with 100 nM 4-OHT or solvent (ethanol) for 24 hours. Values represent the amount of a given lipid as % of total lipids within its class. The two values represent biologically independent experiments. [file 2049-3002-1-3-S7.pdf]

Table S3: Lipid composition in RPE-myrAkt-ER cells after depletion of SREBP

lipid	% of total															
**Ceramide**	**siCtr ethanol**		**siCtr 4-OHT**		**siBP1 ethanol**		**siBP1 4-OHT**		**siBP2 ethanol**		**siBP2 4-OHT**		**siBP1+2 ethanol**		**siBP1+2 4-OHT**	
14:0-Cer	3.1	2.8	3.9	4.8	1.9	1.7	2.0	2.0	2.9	3.6	4.2	4.9	3.0	2.7	2.2	3.2
16:1-Cer	3.3	3.3	3.0	2.6	2.9	2.7	1.5	1.8	3.3	3.7	2.8	2.9	2.1	2.0	2.1	3.0
16:0-Cer	38.2	39.1	32.4	34.1	35.5	32.9	27.4	28.5	45.3	42.0	36.6	35.1	46.9	47.4	41.3	41.5
18:0-Cer	1.1	1.1	0.9	1.0	1.8	1.7	0.7	0.9	2.6	2.8	1.5	1.6	3.0	2.9	1.5	1.6
22:1-Cer	1.2	1.5	2.1	1.6	1.1	1.0	0.9	1.0	1.2	1.3	1.8	1.5	1.3	1.0	1.1	1.2
22:0-Cer	2.5	2.4	2.6	2.2	4.4	3.6	2.6	2.9	4.8	5.4	4.7	4.1	6.0	6.0	5.1	4.9
24:1-Cer	18.8	18.6	21.7	20.4	13.3	13.9	17.6	17.0	11.9	11.9	14.5	16.0	8.8	8.4	8.6	8.4
24:0-Cer	20.4	19.4	20.1	19.1	29.2	31.1	36.0	33.4	19.4	19.7	20.4	19.6	22.2	23.1	29.3	28.1
26:1-Cer	6.9	6.7	8.4	9.1	4.1	5.1	5.5	6.2	3.7	4.4	7.2	7.7	2.4	2.5	3.1	2.5
26:0-Cer	4.6	5.0	5.0	5.1	5.8	6.4	5.9	6.5	4.8	5.3	6.2	6.6	4.2	4.1	5.7	5.6
**Total**	100.0	100.0	100.0	100.0	100.0	100.0	100.0	100.0	100.0	100.0	100.0	100.0	100.0	100.0	100.0	100.0

**Cardiolipin**	**siCtr ethanol**		**siCtr 4-OHT**		**siBP1 ethanol**		**siBP1 4-OHT**		**siBP2 ethanol**		**siBP2 4-OHT**		**siBP1+2 ethanol**		**siBP1+2 4-OHT**	
64:3-CL	0.1	0.4	0.4	0.3	0.1	0.0	0.1	0.3	0.1	0.2	0.2	0.2	0.1	0.1	0.1	0.1
64:2-CL	0.2	0.2	0.3	0.3	0.2	0.2	0.2	0.2	0.1	0.1	0.3	0.4	0.1	0.2	0.1	0.1
66:4-CL	1.5	1.2	2.6	3.2	0.5	0.6	0.7	1.3	0.6	0.8	1.0	1.3	0.4	0.5	0.5	0.5
66:3-CL	1.4	1.5	2.0	2.8	0.9	1.2	1.1	1.7	0.9	1.1	1.5	1.9	0.6	0.6	0.7	0.7
66:2-CL	1.0	1.2	0.8	1.0	0.8	0.8	0.7	0.9	1.0	1.0	1.3	1.3	0.3	0.6	0.5	0.4
68:6-CL	0.8	0.2	0.5	0.7	0.2	0.1	0.4	0.3	0.2	0.5	0.4	0.4	0.2	0.3	0.2	0.3
68:5-CL	3.0	3.3	3.9	4.9	1.4	2.0	2.8	2.4	2.1	2.1	2.3	2.8	1.3	1.5	1.6	1.8
68:4-CL	8.7	7.8	11.0	12.1	5.0	5.3	6.7	7.8	4.6	5.4	6.1	6.8	3.8	4.0	3.7	3.6
68:3-CL	6.2	6.9	6.5	7.3	3.6	5.0	4.7	5.2	3.7	3.8	4.6	5.0	2.6	2.9	2.9	3.4
68:2-CL	4.2	3.9	3.7	3.2	3.1	3.8	3.7	4.3	3.6	3.5	4.1	3.8	2.1	2.4	2.4	2.1
68:1-CL	0.8	0.9	0.5	0.5	0.7	1.3	0.9	0.7	1.0	0.8	0.9	1.0	0.5	0.5	0.7	0.6
70:7-CL	0.4	0.4	0.8	0.4	0.5	0.4	0.6	0.2	0.8	0.8	0.6	0.6	0.3	0.5	0.6	0.6
70:6-CL	2.4	3.9	2.4	3.2	3.7	2.0	3.0	2.7	3.0	2.9	3.3	2.9	2.8	2.8	2.8	2.8
70:5-CL	13.7	12.8	12.4	10.9	11.8	11.2	12.2	11.7	9.1	9.4	9.9	9.6	8.8	9.0	8.2	8.7
70:4-CL	14.8	14.2	15.1	13.7	13.0	12.9	12.3	12.6	11.4	10.9	10.6	10.5	9.5	9.6	9.4	10.3
70:3-CL	6.5	6.7	6.3	5.1	6.4	6.1	5.0	6.1	5.8	5.8	5.9	6.1	4.9	5.1	5.1	5.5
70:2-CL	1.0	1.2	0.9	0.6	0.9	1.8	1.2	1.3	1.8	1.8	1.6	1.9	1.5	1.6	2.3	1.8
72:8-CL	1.0	0.6	0.5	0.5	0.9	0.7	0.8	0.5	1.2	0.8	1.0	1.1	1.3	1.0	1.2	1.3
72:7-CL	1.4	1.5	1.9	1.6	2.6	2.9	2.2	2.7	3.1	3.0	2.9	2.6	3.8	3.9	3.7	4.0
72:6-CL	8.1	9.2	6.0	6.2	13.5	10.4	10.6	10.8	11.6	11.6	10.7	10.7	13.7	13.7	13.8	14.3
72:5-CL	12.5	10.4	10.5	9.2	15.5	14.5	14.7	13.1	15.0	15.3	14.2	13.1	16.9	17.0	16.8	15.8
72:4-CL	5.2	6.4	4.2	4.7	6.2	5.1	5.7	5.9	7.6	6.8	5.9	6.3	7.8	7.7	7.5	7.4
72:3-CL	1.3	1.7	1.3	0.9	1.4	1.2	1.7	1.5	2.5	2.9	2.1	2.2	2.6	2.9	3.0	2.7
74:8-CL	1.4	0.9	2.1	3.6	3.2	4.4	3.5	1.8	3.3	2.6	2.8	2.5	4.5	4.3	4.3	3.4
74:7-CL	1.1	1.4	1.5	1.3	3.0	2.9	2.4	2.3	3.1	2.5	2.9	2.4	5.1	3.8	4.1	3.9
74:6-CL	1.0	1.3	1.4	1.6	0.7	2.5	2.0	1.7	2.2	2.9	2.2	2.2	3.5	2.8	2.9	3.4
74:5-CL	0.3	0.2	0.3	0.2	0.2	0.7	0.1	0.3	0.5	0.8	0.5	0.6	0.8	0.7	0.8	0.7
**Total**	100.0	100.0	100.0	100.0	100.0	100.0	100.0	100.0	100.0	100.0	100.0	100.0	100.0	100.0	100.0	100.0

**Diacylglycerol**	**siCtr ethanol**		**siCtr 4-OHT**		**siBP1 ethanol**		**siBP1 4-OHT**		**siBP2 ethanol**		**siBP2 4-OHT**		**siBP1+2 ethanol**		**siBP1+2 4-OHT**	
30:2-DG	0.3	0.3	0.7	1.0	0.1	0.1	0.3	0.3	0.1	0.1	0.4	0.6	0.1	0.0	0.1	0.1
30:1-DG	1.8	1.9	2.6	2.9	1.1	1.4	1.8	1.8	0.9	1.2	1.9	2.4	0.4	0.5	0.6	0.8
30:0-DG	2.7	2.4	2.2	2.3	3.2	3.2	2.6	2.6	2.9	3.0	3.0	3.2	3.1	3.1	2.8	3.4
32:3-DG	0.7	0.6	0.8	0.9	1.9	1.5	1.1	0.8	0.7	0.4	0.5	0.5	1.0	1.3	0.4	0.4
32:2-DG	4.4	4.2	6.5	7.3	1.8	2.1	3.3	3.5	1.6	1.8	3.8	4.2	0.9	0.9	1.0	1.2
32:1-DG	10.8	11.0	10.9	12.0	9.2	10.0	10.5	10.5	7.4	8.9	11.1	11.1	6.3	6.0	6.8	7.5
32:0-DG	6.2	5.6	4.6	4.6	12.8	11.7	8.1	7.4	9.9	8.5	7.6	6.4	11.6	11.0	11.3	13.0
34:3-DG	1.4	1.3	2.8	2.8	0.4	0.5	1.0	1.1	0.6	0.7	1.4	1.5	0.5	0.5	0.6	0.7
34:2-DG	15.5	15.1	19.5	20.8	6.5	6.9	10.7	11.7	6.5	7.5	12.9	13.5	4.6	5.0	5.8	6.7
34:1-DG	20.8	22.5	18.9	19.9	21.7	22.8	22.4	22.7	21.3	22.1	22.1	19.8	19.7	21.5	21.3	20.6
34:0-DG	3.9	4.1	2.9	3.0	12.1	10.6	7.6	7.2	9.8	7.9	5.6	4.3	13.7	15.6	15.6	14.8
36:5-DG	0.4	0.2	0.3	0.3	0.1	0.3	0.3	0.2	0.4	0.5	0.5	0.6	0.4	0.4	0.4	0.4
36:4-DG	1.1	0.8	1.3	1.0	0.8	0.8	0.9	0.8	1.4	1.3	1.3	1.6	1.0	0.9	1.1	1.1
36:3-DG	2.7	2.8	4.0	3.7	1.3	1.3	2.1	2.2	1.9	2.0	2.6	3.0	1.7	2.1	2.0	1.9
36:2-DG	11.8	12.5	11.6	9.4	7.3	7.7	10.4	10.9	8.7	9.6	9.6	10.4	7.3	7.3	7.6	6.9
36:1-DG	4.5	4.9	3.2	2.5	7.0	7.3	6.2	6.2	8.1	7.8	4.7	4.6	10.2	9.2	8.8	7.9
36:0-DG	0.6	0.5	0.2	0.2	1.2	1.1	0.9	0.8	1.3	1.1	0.6	0.5	2.1	1.6	1.7	1.5
38:6-DG	0.5	0.4	0.3	0.2	0.5	0.4	0.5	0.4	0.8	0.9	0.5	0.7	0.9	0.9	0.7	0.6
38:5-DG	1.2	1.0	0.7	0.6	1.3	1.1	1.0	0.9	2.1	2.0	1.5	1.5	1.7	1.8	1.6	1.6
38:4-DG	2.7	2.2	1.6	1.2	4.2	3.4	2.4	2.1	6.1	5.2	3.0	3.1	4.5	3.5	3.3	2.8
38:3-DG	3.0	2.8	2.2	1.6	2.6	2.6	2.6	3.0	3.6	3.5	2.5	2.9	3.2	2.6	2.6	2.2
38:2-DG	1.3	1.2	0.9	0.7	0.9	0.9	1.1	1.2	1.0	1.2	0.9	1.1	1.0	0.8	0.7	0.7
38:1-DG	0.2	0.2	0.1	0.1	0.2	0.3	0.3	0.3	0.2	0.2	0.1	0.2	0.3	0.4	0.3	0.2
40:7-DG	0.2	0.2	0.1	0.1	0.1	0.1	0.1	0.1	0.2	0.3	0.3	0.2	0.3	0.2	0.2	0.2
40:6-DG	0.5	0.4	0.3	0.3	0.5	0.4	0.5	0.4	1.0	0.8	0.6	0.7	1.1	0.9	0.9	0.9
40:5-DG	0.6	0.5	0.5	0.4	0.8	0.7	0.7	0.6	1.1	0.9	0.7	0.8	1.7	1.3	1.3	1.1
40:4-DG	0.4	0.3	0.4	0.4	0.4	0.5	0.5	0.4	0.5	0.5	0.5	0.5	0.8	0.7	0.6	0.5
**Total**	100.0	100.0	100.0	100.0	100.0	100.0	100.0	100.0	100.0	100.0	100.0	100.0	100.0	100.0	100.0	100.0

**Free fatty acids**	**siCtr ethanol**		**siCtr 4-OHT**		**siBP1 ethanol**		**siBP1 4-OHT**		**siBP2 ethanol**		**siBP2 4-OHT**		**siBP1+2 ethanol**		**siBP1+2 4-OHT**	
14:0-FFA	0.4	0.2	0.3	0.3	0.2	0.2	0.2	0.3	0.2	0.3	0.3	0.3	0.3	0.3	0.4	0.3
16:1-FFA	3.1	3.2	3.9	4.0	1.6	1.9	2.1	2.0	1.5	2.0	2.8	3.2	1.0	0.8	1.0	1.1
16:0-FFA	7.3	7.3	5.7	6.6	7.5	11.2	10.3	11.5	7.7	8.9	8.0	9.9	8.9	6.3	9.1	8.5
18:3-FFA	0.4	0.5	0.9	1.0	0.4	0.4	0.5	0.4	0.4	0.4	0.5	0.5	0.6	0.5	0.5	0.5
18:2-FFA	3.3	3.0	6.0	6.3	2.6	2.9	2.8	2.7	2.6	2.9	3.0	3.3	2.8	2.7	2.4	2.2
18:1-FFA	33.0	32.3	35.1	35.7	23.4	26.6	28.9	29.6	26.2	26.0	28.2	29.2	17.9	16.2	16.0	17.2
18:0-FFA	10.7	12.2	8.3	9.5	16.4	19.8	17.0	16.7	15.7	16.4	15.5	16.1	21.2	19.6	21.4	20.8
20:5-FFA	0.7	0.8	0.4	0.4	1.2	1.1	0.5	0.5	1.0	1.1	0.7	0.6	1.2	1.4	1.2	0.9
20:4-FFA	10.5	10.0	5.5	5.0	16.3	12.0	8.4	7.2	16.2	14.2	11.7	8.9	15.1	17.3	12.4	12.2
20:3-FFA	5.0	4.6	7.0	6.8	3.5	2.6	3.8	3.9	3.1	3.1	4.1	4.0	3.1	3.4	2.5	2.9
20:2-FFA	2.3	2.0	3.5	3.6	1.4	1.2	2.1	2.1	1.2	1.2	1.7	1.4	0.9	0.8	0.8	0.8
20:1-FFA	1.0	1.1	1.7	1.6	0.9	0.9	1.4	1.3	0.9	0.9	0.9	0.8	0.5	0.5	0.4	0.6
20:0-FFA	0.2	0.1	0.2	0.2	0.3	0.3	0.4	0.3	0.2	0.3	0.2	0.2	0.5	0.4	0.4	0.4
22:6-FFA	6.2	5.9	3.6	3.1	6.0	4.7	4.3	5.0	6.9	6.4	6.2	5.5	6.6	7.5	8.5	8.6
22:5-FFA	5.9	6.2	4.2	4.2	8.5	6.4	6.3	6.3	7.3	6.9	6.5	5.9	8.3	9.7	9.2	8.3
22:4-FFA	3.5	3.8	4.0	3.6	5.0	3.6	3.7	3.8	3.9	3.7	4.3	4.3	4.8	5.1	5.1	4.8
22:3-FFA	1.1	1.0	2.5	2.3	0.5	0.3	0.8	1.0	0.5	0.4	1.0	1.1	0.4	0.4	0.4	0.5
24:6-FFA	0.8	0.6	0.5	0.3	0.6	0.3	0.5	0.4	0.6	0.5	0.6	0.4	0.3	0.6	0.4	0.4
24:5-FFA	0.5	0.3	0.4	0.3	0.4	0.2	0.3	0.3	0.4	0.2	0.4	0.3	0.4	0.4	0.4	0.4
24:4-FFA	0.3	0.4	1.4	1.0	0.2	0.0	0.2	0.3	0.1	0.1	0.2	0.2	0.2	0.3	0.2	0.3
24:1-FFA	0.5	0.6	1.1	0.9	0.3	0.4	0.7	0.7	0.4	0.6	0.5	0.6	0.2	0.2	0.4	0.4
24:0-FFA	0.7	0.9	0.9	0.8	1.2	1.3	1.8	1.3	1.2	1.4	1.2	1.4	1.9	1.9	3.5	3.7
26:2-FFA	0.4	0.5	1.0	0.7	0.1	0.1	0.3	0.4	0.1	0.2	0.2	0.2	0.1	0.1	0.1	0.1
26:1-FFA	1.3	1.5	1.5	1.3	0.6	0.5	1.2	1.1	0.7	0.7	0.6	0.7	0.6	0.8	0.6	0.7
26:0-FFA	0.8	0.9	0.6	0.5	1.1	1.1	1.3	1.0	0.9	1.2	0.7	0.8	2.2	2.7	2.9	3.4
**Total**	100.0	100.0	100.0	100.0	100.0	100.0	100.0	100.0	100.0	100.0	100.0	100.0	100.0	100.0	100.0	100.0

**Lysophosphatidic acid**	**siCtr ethanol**		**siCtr 4-OHT**		**siBP1 ethanol**		**siBP1 4-OHT**		**siBP2 ethanol**		**siBP2 4-OHT**		**siBP1+2 ethanol**		**siBP1+2 4-OHT**	
14:0-LPC	1.9	2.0	2.5	2.3	1.8	1.8	1.7	1.9	1.8	1.6	2.2	2.3	1.1	1.4	1.2	1.2
16:1-LPC	7.2	6.4	11.6	11.1	4.8	5.0	7.2	6.6	4.1	4.4	7.6	8.0	2.4	3.0	2.9	3.1

16:0-LPC	34.5	36.3	32.8	31.1	37.3	38.3	35.4	36.4	36.9	34.9	37.1	35.4	32.9	32.5	33.2	32.6
18:3-LPC	0.3	0.3	0.3	0.3	0.3	0.2	0.2	0.3	0.3	0.1	0.1	0.2	0.1	0.2	0.1	0.1
18:2-LPC	1.7	1.5	2.6	2.6	1.4	1.1	1.4	1.4	1.1	1.5	1.5	1.4	1.4	1.5	1.5	1.5
18:1-LPC	29.5	30.2	30.0	32.6	24.2	25.4	29.5	27.2	24.2	30.1	28.5	28.5	20.0	22.6	19.7	21.1
18:0-LPC	11.6	11.5	7.5	6.9	19.0	17.2	15.6	15.2	19.2	16.0	13.0	12.2	30.8	27.0	30.6	28.7
20:4-LPC	1.6	1.2	1.6	1.9	1.4	1.1	1.0	0.9	1.4	1.5	1.5	1.4	1.5	1.8	1.4	1.6
20:3-LPC	1.0	0.8	1.0	1.3	0.8	0.6	0.7	0.6	0.6	0.7	0.5	0.7	1.0	1.1	1.1	0.9
20:2-LPC	0.3	0.3	0.4	0.5	0.3	0.3	0.2	0.4	0.2	0.2	0.1	0.2	0.2	0.2	0.2	0.2
20:1-LPC	0.9	0.9	0.6	0.9	0.9	0.8	0.7	0.6	0.8	0.6	0.5	0.4	0.5	0.6	0.5	0.4
20:0-LPC	0.2	0.3	0.2	0.2	0.3	0.2	0.1	0.3	0.3	0.2	0.1	0.1	0.4	0.0	0.4	0.3
22:6-LPC	0.5	0.3	0.2	0.4	0.4	0.3	0.2	0.2	0.5	0.6	0.2	0.4	0.6	0.4	0.4	0.5
22:5-LPC	0.4	0.3	0.3	0.3	0.5	0.4	0.3	0.4	0.5	0.5	0.3	0.4	0.6	0.8	0.6	0.7
22:4-LPC	0.1	0.1	0.2	0.2	0.3	0.1	0.1	0.1	0.2	0.2	0.2	0.2	0.2	0.7	0.2	0.2
16:2-aLPC	0.2	0.1	0.1	0.1	0.2	0.2	0.1	0.2	0.1	0.1	0.1	0.1	0.2	0.2	0.1	0.1
16:1-aLPC	0.7	0.7	0.5	0.5	0.6	0.4	0.4	0.5	0.6	0.5	0.4	0.5	0.4	0.1	0.3	0.4
16:0-aLPC	3.3	3.1	3.5	2.9	2.5	3.1	2.2	2.7	2.5	2.5	2.9	2.8	2.2	0.5	2.8	3.2
18:2-aLPC	0.1	0.1	0.1	0.1	0.1	0.1	0.2	0.1	0.1	0.1	0.1	0.0	0.1	2.2	0.1	0.1
18:1-aLPC	1.1	1.0	0.8	0.9	0.8	0.8	0.5	0.7	1.1	0.9	0.7	0.7	0.8	0.1	0.7	0.8
20:0-aLPC	0.1	0.2	0.1	0.2	0.2	0.2	0.1	0.1	0.3	0.2	0.1	0.1	0.2	0.6	0.1	0.1
22:0-aLPC	0.5	0.5	0.6	0.6	0.5	0.4	0.4	0.7	0.6	0.4	0.4	0.6	0.5	0.2	0.4	0.4
24:1-aLPC	0.7	0.7	0.8	0.8	0.3	0.4	0.2	0.6	0.7	0.6	0.4	0.8	0.5	0.5	0.3	0.4
24:0-aLPC	0.9	0.9	1.2	0.9	1.1	1.1	1.3	1.8	1.5	1.2	1.6	1.9	1.2	0.4	1.1	1.1
26:1-aLPC	0.4	0.4	0.5	0.4	0.1	0.2	0.2	0.4	0.5	0.4	0.3	0.7	0.2	1.2	0.3	0.3
**Total**	100.0	100.0	100.0	100.0	100.0	100.0	100.0	100.0	100.0	100.0	100.0	100.0	100.0	100.0	100.0	100.0

**Monoacylglycerol**	**siCtr ethanol**		**siCtr 4-OHT**		**siBP1 ethanol**		**siBP1 4-OHT**		**siBP2 ethanol**		**siBP2 4-OHT**		**siBP1+2 ethanol**		**siBP1+2 4-OHT**	
16:1-MG	4.2	5.1	6.3	8.9	3.9	5.3	6.1	5.5	2.80	2.3	3.6	3.1	1.0	0.9	0.8	1.4
16:0-MG	44.3	33.6	46.8	38.2	35.7	24.9	26.9	28.8	40.47	33.4	46.2	37.0	31.3	26.6	30.9	35.2
18:1-MG	40.4	51.1	39.0	46.6	48.8	58.9	59.2	55.9	59.87	49.4	34.2	44.5	41.4	53.7	41.4	44.2
18:0-MG	11.1	10.2	8.0	6.4	11.6	10.9	7.8	9.8	17.99	14.8	16.0	15.4	26.3	18.8	26.9	19.1
**Total**	100.0	100.0	100.0	100.0	100.0	100.0	100.0	100.0	121.12	100.0	100.0	100.0	100.0	100.0	100.0	100.0

**Phosphatidic acid**	**siCtr ethanol**		**siCtr 4-OHT**		**siBP1 ethanol**		**siBP1 4-OHT**		**siBP2 ethanol**		**siBP2 4-OHT**		**siBP1+2 ethanol**		**siBP1+2 4-OHT**	
30:1-PA	1.1	0.9	1.6	2.0	1.44	0.8	0.9	0.9	0.3	0.4	1.2	0.9	0.1	0.1	0.1	0.1
30:0-PA	2.3	2.3	2.4	2.7	7.34	4.1	1.7	1.6	2.4	2.9	2.6	1.6	2.9	3.1	2.0	2.8
32:2-PA	1.7	1.6	3.6	3.9	1.28	0.7	2.1	2.1	0.6	0.8	1.8	2.3	0.1	0.1	0.2	0.2
32:1-PA	8.1	8.6	11.1	11.1	14.26	7.9	8.8	9.8	7.0	7.8	9.1	8.7	2.4	2.7	3.4	3.6
32:0-PA	6.4	6.9	6.4	6.8	33.51	18.5	9.2	9.4	16.7	15.4	12.2	9.1	22.1	25.4	20.2	27.1
34:3-PA	1.2	1.1	2.8	2.8	0.87	0.5	1.5	1.3	0.6	0.6	1.1	1.8	0.4	0.5	0.5	0.4
34:2-PA	5.7	5.5	9.4	9.4	5.92	3.3	6.7	7.3	3.7	4.6	7.4	8.3	1.6	1.8	2.5	2.1
34:1-PA	21.5	21.7	22.9	22.5	36.42	20.1	26.1	25.9	23.1	26.6	26.2	23.8	14.6	15.3	17.9	15.1
34:0-PA	3.4	3.1	2.9	2.9	18.81	10.4	7.2	7.0	12.6	12.0	6.9	5.1	22.7	27.3	24.4	24.6
36:4-PA	0.7	0.7	0.8	1.2	1.05	0.6	1.0	0.8	1.1	0.6	0.7	1.9	0.9	0.6	0.9	0.9
36:3-PA	1.6	1.2	2.4	2.9	1.51	0.8	2.0	1.7	1.3	1.0	1.4	3.0	1.3	1.4	1.6	1.2
36:2-PA	20.6	25.0	19.1	17.4	21.49	11.9	9.0	9.5	7.3	7.2	9.0	10.3	4.7	4.5	5.7	4.3
36:1-PA	13.1	13.2	8.3	6.9	16.83	9.3	13.5	11.5	12.4	12.5	12.7	10.8	11.4	9.6	11.2	8.7
36:0-PA	0.6	0.3	0.2	0.3	1.54	0.9	0.8	0.6	1.2	1.1	0.7	0.6	2.4	2.8	2.8	2.3
38:6-PA	0.3	0.2	0.1	0.3	0.79	0.4	0.3	0.4	0.8	0.6	0.2	0.9	1.4	0.5	0.7	0.8
38:5-PA	0.7	0.5	0.4	0.6	1.09	0.6	0.8	1.0	1.4	1.0	0.8	1.9	1.9	0.8	1.1	1.1
38:4-PA	1.3	0.9	0.6	1.1	2.07	1.1	1.2	1.2	2.7	1.9	1.4	2.3	2.3	1.1	1.3	1.3
38:3-PA	1.2	1.0	0.7	1.0	2.09	1.2	1.1	1.3	1.5	0.8	1.1	2.3	1.6	0.9	1.1	0.8
38:2-PA	0.5	0.2	0.2	0.2	0.46	0.3	0.3	0.3	0.3	0.2	0.3	0.6	0.4	0.2	0.3	0.2
40:6-PA	1.5	1.1	0.6	1.4	3.42	1.9	1.6	1.6	1.4	1.0	1.5	1.7	1.8	0.6	1.0	1.0
40:5-PA	2.5	1.5	1.3	1.3	3.86	2.1	2.1	2.1	1.3	0.8	1.8	1.6	2.4	0.6	0.8	0.8
40:4-PA	4.0	2.3	2.3	1.2	5.03	2.8	2.1	2.5	0.4	0.2	0.0	0.6	0.7	0.3	0.4	0.4
**Total**	100.0	100.0	100.0	100.0	181.08	100.0	100.0	100.0	100.0	100.0	100.0	100.0	100.0	100.0	100.0	100.0

**Phosphatidylcholine**	**siCtr ethanol**		**siCtr 4-OHT**		**siBP1 ethanol**		**siBP1 4-OHT**		**siBP2 ethanol**		**siBP2 4-OHT**		**siBP1+2 ethanol**		**siBP1+2 4-OHT**	
26:0-PC	0.0	0.0	0.0	0.0	0.0	0.1	0.0	0.0	0.1	0.1	0.0	0.1	0.1	0.1	0.1	0.1
28:1-PC	0.1	0.2	0.1	0.1	0.1	0.1	0.1	0.1	0.1	0.1	0.1	0.2	0.1	0.1	0.0	0.1
28:0-PC	1.1	1.8	0.9	1.0	1.5	2.2	0.9	1.3	1.1	1.1	1.0	1.4	1.2	0.8	0.7	0.8
30:2-PC	0.3	0.2	0.2	0.2	0.1	0.1	0.1	0.1	0.0	0.1	0.1	0.2	0.0	0.0	0.0	0.0
30:1-PC	3.9	3.2	2.0	2.3	3.1	2.3	1.7	2.5	1.4	1.5	1.5	2.8	1.1	0.8	0.7	0.8
30:0-PC	12.7	13.3	5.3	5.9	16.5	12.3	7.0	9.1	9.0	8.3	6.8	11.1	10.8	6.9	6.6	7.3
32:3-PC	0.1	0.1	0.3	0.3	0.0	0.0	0.1	0.1	0.1	0.1	0.1	0.1	0.1	0.0	0.0	0.0
32:2-PC	2.0	2.0	3.5	4.6	1.1	1.1	1.5	2.2	1.2	1.1	1.4	2.1	0.6	0.5	0.5	0.7
32:1-PC	13.8	12.3	10.7	14.0	12.1	11.7	9.4	14.8	9.3	7.8	8.1	11.2	7.2	5.5	5.3	5.5
32:0-PC	13.2	10.7	6.2	7.7	12.3	9.8	6.4	9.9	8.9	8.1	7.8	11.1	12.2	8.5	9.1	9.8
34:4-PC	0.1	0.1	0.1	0.1	0.0	0.1	0.1	0.0	0.1	0.1	0.1	0.1	0.0	0.1	0.1	0.1
34:3-PC	0.6	0.6	1.8	1.2	0.3	0.3	0.9	0.5	0.6	0.7	0.9	0.8	0.4	0.4	0.4	0.5
34:2-PC	4.1	4.3	10.9	8.8	2.6	2.8	5.6	4.6	4.3	4.6	5.1	5.3	2.5	2.3	2.3	3.0
34:1-PC	16.0	16.7	23.0	25.9	17.0	17.3	19.8	22.7	24.3	20.7	16.0	18.2	16.3	13.8	12.4	14.8
34:0-PC	2.5	2.5	2.9	3.5	3.2	3.0	3.0	3.6	4.2	3.5	2.5	3.0	4.6	3.6	3.3	3.6
36:5-PC	0.4	0.4	0.4	0.3	0.4	0.4	0.4	0.3	0.4	0.5	0.9	0.4	0.4	0.6	0.6	0.6
36:4-PC	1.2	1.3	1.1	0.9	1.4	1.4	1.7	0.9	1.5	2.0	2.9	1.3	1.5	2.5	2.6	2.6
36:3-PC	1.7	1.7	2.4	1.7	1.5	1.5	2.8	1.4	1.7	2.8	3.2	1.8	1.8	2.5	2.6	2.9
36:2-PC	5.6	5.7	8.1	5.1	4.8	5.0	11.4	5.5	6.3	9.8	9.2	5.8	4.8	6.1	6.3	7.2
36:1-PC	2.6	2.7	3.3	2.0	4.5	4.1	7.8	3.9	5.7	7.1	4.7	3.1	6.8	7.4	7.6	8.3
36:0-PC	0.2	0.2	0.2	0.1	0.5	0.5	0.8	0.4	0.6	0.8	0.4	0.3	0.9	1.0	1.0	1.1
38:7-PC	0.1	0.1	0.1	0.1	0.1	0.1	0.1	0.1	0.1	0.1	0.2	0.2	0.1	0.2	0.2	0.1
38:6-PC	0.8	0.9	0.6	0.5	0.9	1.0	0.8	0.7	0.9	1.0	1.7	0.8	1.2	2.0	1.9	1.6
38:5-PC	1.6	1.8	1.3	1.2	1.6	1.9	1.7	1.3	1.6	1.8	3.2	1.6	1.9	3.2	3.3	2.9
38:4-PC	1.6	1.8	1.5	1.3	1.9	2.2	2.1	1.5	1.9	2.1	3.6	1.7	2.8	4.6	4.8	4.2
38:3-PC	0.8	0.9	0.8	0.7	0.9	0.9	1.2	0.8	0.8	1.1	1.7	0.8	1.2	2.0	2.1	2.0
38:2-PC	0.4	0.4	0.4	0.4	0.4	0.4	0.8	0.4	0.4	0.6	0.8	0.4	0.5	0.8	0.8	0.8
38:1-PC	0.1	0.1	0.1	0.0	0.1	0.1	0.2	0.1	0.1	0.2	0.2	0.1	0.2	0.2	0.3	0.2
40:8-PC	0.1	0.1	0.1	0.1	0.1	0.1	0.1	0.1	0.1	0.1	0.1	0.1	0.2	0.2	0.2	0.1
40:7-PC	1.0	1.1	0.7	0.6	0.8	1.2	0.7	0.8	0.9	0.8	1.0	1.0	1.2	1.7	1.6	1.0
40:6-PC	1.1	1.3	0.9	0.7	1.3	1.8	1.2	1.1	1.2	1.2	1.7	1.2	2.0	3.3	3.2	2.2
40:5-PC	0.8	0.9	0.7	0.6	1.0	1.2	1.0	0.8	0.8	0.8	1.3	0.9	1.6	2.7	2.8	1.9
40:4-PC	0.4	0.4	0.4	0.4	0.4	0.5	0.5	0.4	0.3	0.3	0.6	0.4	0.7	1.2	1.2	1.0
40:3-PC	0.1	0.1	0.2	0.1	0.1	0.1	0.1	0.1	0.1	0.1	0.2	0.1	0.2	0.3	0.3	0.3
40:2-PC	0.1	0.1	0.2	0.0	0.0	0.1	0.2	0.2	0.2	0.1	0.3	0.2	0.1	0.1	0.1	0.1
42:9-PC	0.1	0.1	0.1	0.1	0.1	0.2	0.1	0.1	0.1	0.1	0.1	0.1	0.3	0.3	0.3	0.2
42:8-PC	0.1	0.1	0.1	0.1	0.1	0.3	0.1	0.1	0.1	0.1	0.1	0.2	0.2	0.2	0.2	0.1
42:7-PC	0.2	0.3	0.2	0.2	0.2	0.3	0.2	0.2	0.2	0.2	0.2	0.3	0.3	0.3	0.4	0.2
42:6-PC	0.2	0.2	0.2	0.2	0.2	0.3	0.2	0.2	0.2	0.2	0.3	0.2	0.4	0.6	0.6	0.4
42:5-PC	0.1	0.1	0.2	0.2	0.2	0.2	0.2	0.1	0.2	0.2	0.2	0.2	0.3	0.5	0.5	0.3
42:4-PC	0.2	0.2	0.2	0.2	0.2	0.2	0.2	0.2	0.2	0.2	0.4	0.2	0.3	0.4	0.4	0.2
44:9-PC	0.1	0.1	0.1	0.0	0.1	0.2	0.1	0.1	0.1	0.1	0.1	0.1	0.1	0.5	0.6	0.2
44:8-PC	0.0	0.1	0.0	0.0	0.0	0.1	0.0	0.0	0.1	0.0	0.0	0.1	0.1	0.1	0.1	0.1
44:7-PC	0.1	0.1	0.1	0.1	0.1	0.1	0.1	0.1	0.1	0.1	0.1	0.1	0.2	0.2	0.2	0.1
44:6-PC	0.1	0.1	0.1	0.1	0.1	0.1	0.1	0.1	0.1	0.1	0.2	0.1	0.2	0.3	0.2	0.2
44:5-PC	0.3	0.3	0.3	0.0	0.2	0.1	0.3	0.3	0.4	0.4	0.6	0.3	0.4	0.6	0.6	0.5
46:9-PC	0.0	0.0	0.0	0.0	0.0	0.0	0.0	0.0	0.0	0.0	0.0	0.0	0.1	0.1	0.1	0.0
46:8-PC	0.0	0.0	0.0	0.0	0.0	0.0	0.0	0.0	0.0	0.0	0.0	0.0	0.1	0.0	0.0	0.0
46:7-PC	0.1	0.1	0.1	0.1	0.0	0.0	0.1	0.0	0.1	0.1	0.1	0.1	0.1	0.1	0.1	0.1

26:0-aPC	0.0	0.0	0.0	0.0	0.1	0.1	0.1	0.1	0.1	0.1	0.1	0.1	0.1	0.1	0.1	0.1
28:1-aPC	0.0	0.0	0.0	0.0	0.0	0.0	0.0	0.0	0.0	0.0	0.0	0.0	0.0	0.0	0.0	0.0
28:0-aPC	0.1	0.1	0.1	0.2	0.2	0.1	0.3	0.3	0.1	0.1	0.2	0.3	0.1	0.1	0.3	0.3
30:1-aPC	0.2	0.1	0.1	0.1	0.2	0.2	0.1	0.1	0.1	0.1	0.0	0.0	0.2	0.1	0.2	0.1
30:0-aPC	0.1	0.2	0.1	0.1	0.1	0.1	0.0	0.1	0.1	0.1	0.1	0.1	0.1	0.1	0.2	0.1
32:2-aPC	0.1	0.1	0.1	0.1	0.1	0.1	0.0	0.1	0.1	0.1	0.1	0.1	0.1	0.1	0.1	0.0
32:1-aPC	0.1	0.1	0.3	0.3	0.1	0.1	0.1	0.1	0.1	0.2	0.1	0.2	0.1	0.1	0.1	0.1
32:0-aPC	0.3	0.3	0.4	0.5	0.2	0.3	0.2	0.3	0.3	0.3	0.3	0.4	0.4	0.3	0.4	0.5
34:2-aPC	0.4	0.4	0.2	0.3	0.1	0.3	0.1	0.4	0.6	0.1	0.6	0.4	0.4	0.4	0.5	0.3
34:1-aPC	0.5	0.5	0.6	0.4	0.3	0.4	0.7	0.4	0.5	0.8	0.8	0.5	0.4	0.6	0.7	0.8
34:0-aPC	0.1	0.1	0.2	0.1	0.1	0.1	0.1	0.1	0.2	0.3	0.2	0.2	0.3	0.3	0.3	0.4
36:5-aPC	0.2	0.2	0.1	0.1	0.1	0.2	0.1	0.1	0.2	0.2	0.3	0.1	0.2	0.2	0.2	0.2
36:4-aPC	0.3	0.3	0.2	0.2	0.2	0.3	0.2	0.2	0.2	0.2	0.4	0.2	0.2	0.4	0.4	0.4
36:3-aPC	0.2	0.2	0.2	0.2	0.2	0.2	0.2	0.2	0.2	0.2	0.4	0.2	0.2	0.3	0.4	0.4
36:2-aPC	0.4	0.5	0.2	0.3	0.2	0.5	0.2	0.2	0.6	0.2	0.4	0.5	0.5	0.4	0.5	0.4
36:1-aPC	0.3	0.3	0.1	0.1	0.1	0.4	0.1	0.1	0.1	0.1	0.2	0.1	0.6	0.2	0.3	0.3
38:6-aPC	0.4	0.5	0.3	0.3	0.3	0.5	0.3	0.3	0.3	0.4	0.4	0.4	0.4	0.7	0.7	0.5
38:5-aPC	0.8	0.8	0.6	0.5	0.6	0.9	0.5	0.5	0.6	0.6	0.8	0.7	0.8	1.1	1.2	0.8
38:4-aPC	0.5	0.6	0.5	0.4	0.4	0.7	0.4	0.4	0.5	0.5	0.7	0.5	0.7	1.0	1.1	0.8
38:3-aPC	0.2	0.2	0.3	0.3	0.1	0.2	0.1	0.2	0.1	0.1	0.1	0.2	0.2	0.3	0.2	0.2
40:7-aPC	0.3	0.3	0.2	0.2	0.2	0.5	0.2	0.2	0.3	0.3	0.2	0.3	0.3	0.3	0.3	0.2
40:6-aPC	0.4	0.5	0.4	0.3	0.3	0.7	0.3	0.3	0.4	0.4	0.4	0.4	0.6	0.7	0.7	0.5
40:5-aPC	0.4	0.4	0.4	0.3	0.3	0.5	0.3	0.3	0.4	0.3	0.3	0.4	0.6	0.7	0.6	0.6
40:4-aPC	0.2	0.2	0.3	0.3	0.2	0.3	0.2	0.2	0.2	0.2	0.2	0.2	0.3	0.4	0.3	0.3
40:3-aPC	0.1	0.1	0.1	0.1	0.0	0.1	0.0	0.1	0.0	0.0	0.0	0.1	0.1	0.1	0.1	0.1
40:2-aPC	0.0	0.0	0.0	0.0	0.0	0.0	0.0	0.0	0.0	0.0	0.0	0.0	0.0	0.0	0.0	0.0
42:7-aPC	0.1	0.1	0.1	0.1	0.1	0.1	0.1	0.1	0.1	0.1	0.1	0.1	0.1	0.1	0.1	0.1
42:6-aPC	0.1	0.1	0.1	0.1	0.1	0.2	0.2	0.1	0.1	0.3	0.2	0.2	0.2	0.2	0.2	0.2
42:5-aPC	0.1	0.1	0.1	0.1	0.1	0.2	0.1	0.1	0.1	0.1	0.1	0.1	0.2	0.1	0.2	0.1
42:4-aPC	0.1	0.1	0.1	0.1	0.1	0.2	0.1	0.1	0.0	0.1	0.1	0.1	0.1	0.1	0.1	0.1
44:7-aPC	0.0	0.0	0.0	0.0	0.0	0.0	0.0	0.0	0.0	0.1	0.0	0.0	0.0	0.0	0.0	0.0
44:6-aPC	0.1	0.1	0.1	0.1	0.1	0.2	0.1	0.1	0.1	0.2	0.0	0.2	0.2	0.0	0.0	0.0
44:5-aPC	0.1	0.1	0.1	0.2	0.2	0.3	0.2	0.2	0.2	0.2	0.0	0.2	0.2	0.0	0.0	0.0
44:4-aPC	0.1	0.1	0.1	0.0	0.1	0.2	0.1	0.2	0.2	0.2	0.0	0.1	0.1	0.0	0.1	0.0
44:3-aPC	0.0	0.0	0.0	0.0	0.0	0.0	0.1	0.1	0.1	0.1	0.0	0.0	0.0	0.0	0.0	0.0
44:2-aPC	0.0	0.0	0.0	0.0	0.0	0.0	0.0	0.0	0.0	0.0	0.0	0.0	0.0	0.0	0.0	0.0
46:7-aPC	0.0	0.1	0.0	0.0	0.0	0.1	0.0	0.0	0.1	0.1	0.0	0.1	0.1	0.0	0.0	0.0
46:6-aPC	0.1	0.1	0.0	0.0	0.0	0.1	0.0	0.0	0.0	0.0	0.0	0.1	0.1	0.0	0.1	0.1
46:5-aPC	0.1	0.0	0.0	0.0	0.1	0.1	0.0	0.0	0.1	0.1	0.0	0.1	0.1	0.0	0.0	0.1
46:4-aPC	0.0	0.0	0.0	0.0	0.0	0.1	0.0	0.0	0.0	0.0	0.0	0.0	0.0	0.0	0.0	0.0
46:3-aPC	0.0	0.0	0.0	0.0	0.0	0.0	0.0	0.0	0.0	0.0	0.0	0.0	0.0	0.0	0.0	0.0
**Total**	100.0	100.0	100.0	100.0	100.0	100.0	100.0	100.0	100.0	100.0	100.0	100.0	100.0	100.0	100.0	100.0

**Phosphatidylethanolamine**	**siCtr ethanol**		**siCtr 4-OHT**		**siBP1 ethanol**		**siBP1 4-OHT**		**siBP2 ethanol**		**siBP2 4-OHT**		**siBP1+2 ethanol**		**siBP1+2 4-OHT**	
30:1-PE	0.0	0.0	0.1	0.1	0.1	0.1	0.0	0.1	0.0	0.0	0.1	0.0	0.0	0.0	0.0	0.0
30:0-PE	0.0	0.0	0.0	0.1	0.0	0.0	0.0	0.0	0.0	0.0	0.1	0.0	0.0	0.0	0.0	0.0
32:2-PE	0.1	0.2	0.2	0.4	0.1	0.2	0.2	0.2	0.1	0.1	0.2	0.2	0.1	0.0	0.0	0.0
32:1-PE	0.7	1.0	0.9	1.6	1.2	1.2	1.0	1.0	0.5	0.6	0.9	0.7	0.7	0.4	0.3	0.5
32:0-PE	0.1	0.2	0.2	0.4	0.2	0.2	0.2	0.2	0.2	0.2	0.3	0.2	0.3	0.1	0.1	0.2
34:3-PE	0.1	0.1	0.2	0.2	0.1	0.1	0.1	0.1	0.1	0.1	0.1	0.1	0.0	0.1	0.0	0.0
34:2-PE	1.9	2.4	2.3	3.0	1.8	1.8	2.1	2.2	1.3	1.4	1.7	1.8	1.5	1.2	0.8	1.0
34:1-PE	5.3	6.6	5.2	7.5	8.1	8.4	7.2	7.3	4.9	4.9	5.5	5.0	6.6	4.5	3.2	3.8
34:0-PE	0.5	0.6	0.5	0.7	0.8	0.8	0.7	0.7	0.5	0.5	0.6	0.5	0.8	0.5	0.4	0.5
36:4-PE	1.6	1.9	1.3	1.2	1.1	1.3	1.2	1.2	0.9	0.9	0.9	0.9	0.8	0.9	0.5	1.0
36:3-PE	1.0	1.2	1.2	1.2	0.8	0.9	1.1	1.0	1.0	1.0	0.9	1.0	0.9	0.9	0.6	1.0
36:2-PE	7.2	8.3	7.4	7.9	5.9	7.4	8.0	8.0	7.4	7.6	6.7	7.1	6.8	6.8	4.4	6.4
36:1-PE	7.0	8.0	6.3	7.0	9.7	11.0	9.9	9.5	9.5	8.8	7.9	7.7	12.6	11.7	8.2	9.3
36:0-PE	0.7	0.7	0.6	0.7	1.1	1.1	1.0	1.0	1.0	0.9	0.8	0.7	1.4	1.2	0.8	0.9
38:6-PE	1.3	1.4	1.4	1.2	1.1	1.0	1.2	1.2	1.3	1.4	1.4	1.5	0.9	1.0	1.0	1.1
38:5-PE	4.2	4.7	4.6	4.4	3.0	2.9	3.9	3.8	3.6	3.5	3.7	3.9	2.6	3.1	2.7	3.5
38:4-PE	7.1	8.1	8.1	7.4	6.9	7.4	8.5	8.5	7.5	6.9	7.0	7.3	7.9	8.6	6.8	9.6
38:3-PE	1.9	2.3	2.2	2.0	1.9	2.2	2.3	2.4	2.3	2.5	2.2	2.4	2.4	2.6	1.7	2.9
38:2-PE	0.4	0.4	0.4	0.4	0.3	0.4	0.4	0.5	0.4	0.5	0.4	0.5	0.4	0.5	0.2	0.4
38:1-PE	0.1	0.1	0.1	0.1	0.1	0.1	0.1	0.1	0.1	0.1	0.1	0.1	0.1	0.1	0.0	0.1
40:8-PE	0.1	0.1	0.1	0.1	0.0	0.0	0.0	0.0	0.0	0.0	0.1	0.1	0.1	0.0	0.1	0.0
40:7-PE	2.2	1.7	1.9	1.8	0.9	0.9	1.1	1.2	1.5	1.7	1.9	2.0	1.0	1.0	1.6	1.2
40:6-PE	4.1	3.8	3.9	3.6	3.2	3.0	3.4	3.5	4.4	4.3	4.4	4.3	3.8	4.1	6.1	4.6
40:5-PE	2.8	2.9	3.1	2.9	3.2	3.1	3.5	3.5	3.8	3.5	3.6	3.3	3.4	3.8	4.4	4.0
40:4-PE	1.2	1.3	1.4	1.4	1.7	1.5	1.8	1.7	1.7	1.5	1.6	1.5	1.6	1.8	1.9	1.8
40:3-PE	0.1	0.2	0.3	0.2	0.2	0.2	0.3	0.3	0.2	0.3	0.2	0.2	0.2	0.3	0.2	0.3
40:2-PE	0.0	0.0	0.1	0.1	0.0	0.0	0.0	0.0	0.0	0.0	0.0	0.0	0.0	0.0	0.0	0.0
40:1-PE	0.0	0.0	0.0	0.0	0.0	0.0	0.0	0.0	0.0	0.0	0.0	0.0	0.0	0.0	0.0	0.1
42:8-PE	0.1	0.0	0.0	0.0	0.0	0.0	0.0	0.0	0.0	0.0	0.0	0.0	0.0	0.0	0.0	0.0
42:7-PE	0.1	0.1	0.1	0.1	0.1	0.0	0.0	0.0	0.1	0.1	0.1	0.1	0.0	0.1	0.1	0.1
42:6-PE	0.2	0.1	0.2	0.1	0.1	0.1	0.3	0.3	0.5	0.3	0.1	0.1	0.1	0.2	0.1	0.1
42:5-PE	0.2	0.1	0.1	0.1	0.1	0.0	0.3	0.3	0.6	0.2	0.0	0.1	0.1	0.1	0.1	0.1
42:4-PE	0.1	0.0	0.0	0.0	0.1	0.0	0.1	0.1	0.1	0.0	0.0	0.0	0.1	0.0	0.1	0.0
32:2-aPE	0.1	0.1	0.1	0.1	0.1	0.1	0.1	0.1	0.1	0.1	0.1	0.1	0.1	0.0	0.0	0.0
32:1-aPE	0.1	0.1	0.1	0.1	0.2	0.1	0.1	0.1	0.1	0.2	0.2	0.1	0.1	0.0	0.0	0.1
32:0-aPE	0.0	0.0	0.0	0.0	0.0	0.0	0.0	0.0	0.0	0.0	0.0	0.0	0.0	0.0	0.0	0.0
34:3-aPE	0.1	0.1	0.1	0.1	0.1	0.1	0.2	0.1	0.1	0.1	0.1	0.1	0.1	0.1	0.1	0.1
34:2-aPE	1.2	1.5	1.1	1.3	1.5	1.6	1.6	1.5	1.4	1.4	1.2	1.3	1.2	1.2	0.8	1.0
34:1-aPE	0.3	0.4	0.2	0.4	0.5	0.6	0.4	0.4	0.5	0.5	0.5	0.4	0.4	0.3	0.2	0.3
36:6-aPE	0.7	0.8	0.7	0.6	0.6	0.6	0.6	0.6	0.6	0.7	0.6	0.6	0.5	0.5	0.4	0.6
36:5-aPE	3.4	4.0	3.7	3.5	3.3	3.1	3.7	3.6	3.7	3.6	3.2	3.4	2.7	3.2	2.5	3.3
36:4-aPE	0.7	0.9	1.0	0.8	0.7	0.8	0.8	0.8	0.8	0.8	0.7	0.8	0.6	0.7	0.5	0.8
36:3-aPE	0.3	0.3	0.4	0.3	0.3	0.3	0.4	0.4	0.3	0.4	0.3	0.4	0.3	0.3	0.2	0.4
36:2-aPE	0.5	0.7	0.6	0.6	0.6	0.8	0.8	0.8	0.7	0.8	0.6	0.7	0.8	0.8	0.5	0.9
38:7-aPE	4.6	3.7	3.8	3.3	2.8	2.6	2.5	2.8	3.1	3.5	3.2	3.4	2.3	2.9	3.8	2.9
38:6-aPE	7.4	6.7	7.0	6.2	6.2	5.7	5.9	6.0	6.6	6.8	6.4	6.3	4.8	5.6	7.0	5.9
38:5-aPE	7.4	6.8	7.3	6.6	7.6	6.6	7.2	7.5	7.9	8.0	8.0	7.9	7.8	6.8	10.0	7.4
38:4-aPE	1.6	1.6	2.1	1.9	1.3	1.2	1.4	1.6	1.5	1.4	1.7	2.0	3.1	1.3	2.1	1.7
40:8-aPE	2.5	1.6	2.2	2.1	2.0	1.9	1.4	1.2	1.5	1.7	2.1	2.1	1.5	1.8	1.9	1.7
40:7-aPE	6.6	4.5	5.4	4.8	6.5	6.0	4.3	4.2	5.6	6.2	6.7	6.4	6.3	7.5	8.3	7.2
40:6-aPE	5.1	3.8	4.5	4.1	5.4	4.5	4.1	4.0	4.8	5.0	5.5	5.0	5.1	5.7	8.0	5.9
40:5-aPE	2.1	1.6	2.2	2.0	2.4	1.8	1.9	1.8	2.1	2.1	2.4	2.2	2.1	2.3	3.4	2.5
40:4-aPE	0.7	0.6	0.8	0.7	0.6	0.5	0.5	0.5	0.5	0.6	0.7	0.7	0.6	0.6	0.9	0.7
42:8-aPE	0.2	0.1	0.2	0.2	0.2	0.2	0.1	0.1	0.1	0.1	0.2	0.2	0.1	0.2	0.1	0.1
42:7-aPE	0.7	0.5	0.6	0.6	0.8	0.8	0.5	0.4	0.5	0.6	0.6	0.7	0.7	0.8	0.8	0.8
42:6-aPE	0.6	0.4	0.6	0.5	0.8	0.7	0.5	0.4	0.5	0.5	0.6	0.6	0.6	0.6	0.8	0.6
42:5-aPE	0.3	0.2	0.3	0.3	0.4	0.4	0.3	0.3	0.2	0.3	0.3	0.3	0.3	0.4	0.5	0.3
42:4-aPE	0.0	0.0	0.0	0.0	0.0	0.0	0.0	0.0	0.0	0.0	0.0	0.0	0.0	0.0	0.1	0.0
44:8-aPE	0.1	0.0	0.0	0.1	0.1	0.1	0.1	0.1	0.1	0.1	0.0	0.1	0.0	0.1	0.1	0.0
44:7-aPE	0.1	0.1	0.1	0.1	0.3	0.3	0.2	0.2	0.2	0.2	0.2	0.2	0.1	0.2	0.2	0.1
44:6-aPE	0.2	0.2	0.2	0.2	0.4	0.4	0.3	0.3	0.4	0.4	0.3	0.3	0.2	0.2	0.2	0.2
44:5-aPE	0.1	0.1	0.1	0.1	0.2	0.1	0.1	0.1	0.1	0.1	0.1	0.1	0.1	0.1	0.1	0.1
46:9-aPE	0.0	0.0	0.0	0.0	0.0	0.0	0.0	0.0	0.0	0.0	0.0	0.0	0.0	0.0	0.0	0.0

46:8-aPE	0.1	0.1	0.1	0.1	0.2	0.2	0.1	0.1	0.0	0.0	0.0	0.0	0.0	0.0	0.0	0.0
46:7-aPE	0.1	0.0	0.1	0.1	0.2	0.2	0.1	0.1	0.0	0.0	0.0	0.0	0.0	0.0	0.0	0.0
46:6-aPE	0.0	0.0	0.0	0.0	0.1	0.1	0.1	0.1	0.0	0.0	0.0	0.1	0.0	0.0	0.0	0.0
46:5-aPE	0.0	0.0	0.0	0.0	0.0	0.0	0.0	0.0	0.0	0.0	0.0	0.0	0.1	0.0	0.0	0.0
**Total**	100.0	100.0	100.0	100.0	100.0	100.0	100.0	100.0	100.0	100.0	100.0	100.0	100.0	100.0	100.0	100.0

**Phosphatidylglycerol**	**siCtr ethanol**		**siCtr 4-OHT**		**siBP1 ethanol**		**siBP1 4-OHT**		**siBP2 ethanol**		**siBP2 4-OHT**		**siBP1+2 ethanol**		**siBP1+2 4-OHT**	
32:2-PG	0.3	0.4	0.7	0.9	0.3	0.4	0.4	0.5	0.2	0.3	0.5	0.6	0.1	0.2	0.2	0.2
32:1-PG	0.9	1.0	1.2	1.5	1.4	1.4	1.5	1.7	0.9	1.2	1.0	1.3	0.9	1.1	1.0	1.2
32:0-PG	0.4	0.4	0.4	0.5	1.0	1.0	0.9	1.0	0.4	0.4	0.3	0.4	0.9	1.1	0.9	1.2
34:4-PG	0.0	0.1	0.1	0.1	0.1	0.0	0.1	0.0	0.0	0.1	0.1	0.2	0.1	0.1	0.0	0.0
34:3-PG	0.8	0.8	1.1	1.4	0.5	0.7	1.0	1.0	0.6	0.8	1.1	1.2	0.4	0.5	0.4	0.5
34:2-PG	3.8	4.0	5.4	6.3	3.4	4.1	5.2	5.7	3.5	4.3	5.3	5.6	2.5	2.9	2.6	3.2
34:1-PG	4.8	5.1	6.2	8.4	7.6	9.1	8.1	8.5	6.6	6.3	6.1	6.2	6.2	6.6	6.0	6.0
34:0-PG	0.7	0.8	1.1	1.2	1.8	2.1	1.7	1.8	1.5	1.4	1.1	1.1	2.4	2.7	2.4	2.4
36:4-PG	0.9	1.0	1.4	1.6	1.1	1.0	1.3	1.2	1.0	1.2	1.6	1.6	1.3	1.6	1.5	1.5
36:3-PG	2.9	3.4	4.4	4.7	2.8	2.9	3.9	4.1	3.1	3.4	4.2	4.5	3.2	3.4	3.1	3.4
36:2-PG	10.3	10.0	10.7	12.4	7.7	9.0	12.1	13.2	8.4	10.6	11.3	11.3	6.8	8.3	7.2	7.1
36:1-PG	3.9	3.8	4.4	5.5	7.3	8.0	7.9	7.7	6.1	5.7	5.2	5.1	9.4	9.4	8.6	8.0
38:7-PG	2.4	2.2	2.2	2.0	1.8	1.9	1.9	1.9	2.0	2.2	2.6	2.6	1.7	1.8	1.7	2.0
38:6-PG	3.6	3.6	3.0	2.8	4.1	4.6	4.0	3.9	4.3	4.5	4.3	4.0	5.1	5.1	5.1	5.2
38:5-PG	4.0	4.0	4.4	3.8	3.9	3.8	4.6	4.2	4.0	4.2	4.7	4.8	4.2	4.5	4.7	5.1
38:4-PG	2.8	2.8	3.7	3.5	2.4	2.3	2.8	3.1	2.4	2.4	3.0	3.0	2.9	3.0	3.0	3.3
38:3-PG	1.6	1.6	2.1	2.3	1.1	1.1	1.5	1.7	1.0	1.2	1.6	1.6	1.1	1.4	1.3	1.1
38:2-PG	0.6	0.6	1.0	1.1	0.5	0.8	0.7	0.9	0.6	0.8	0.7	0.8	0.6	0.6	0.5	0.5
40:9-PG	0.2	0.2	0.2	0.1	0.2	0.2	0.1	0.1	0.2	0.2	0.2	0.2	0.3	0.2	0.3	0.3
40:8-PG	3.0	2.9	2.7	2.1	2.1	1.8	2.0	1.9	2.6	2.7	2.6	2.6	3.1	2.9	3.1	3.0
40:7-PG	17.8	17.0	12.8	10.0	11.7	11.2	11.5	11.0	14.2	14.1	13.0	12.7	10.8	10.4	10.7	11.1
40:6-PG	7.3	7.1	5.7	4.5	6.8	6.1	6.5	6.0	6.5	6.6	5.9	6.0	6.8	6.5	7.1	6.9
40:5-PG	2.0	1.9	1.8	1.5	2.0	1.8	2.1	2.0	2.0	1.8	1.8	1.8	2.3	2.1	2.1	2.0
40:4-PG	0.5	0.5	0.6	0.5	0.4	0.4	0.5	0.4	0.2	0.3	0.3	0.3	0.6	0.6	0.5	0.4
42:11-PG	0.6	0.7	0.5	0.5	0.7	0.5	0.4	0.4	0.8	0.6	0.6	0.5	0.7	0.7	0.8	0.8
42:10-PG	2.7	2.8	2.8	2.6	2.8	2.3	2.2	1.9	3.1	2.4	2.8	2.7	3.2	2.9	3.6	3.4
42:9-PG	2.7	2.7	2.8	2.3	2.5	2.1	2.1	1.8	2.4	2.1	2.3	2.2	2.8	2.5	2.9	2.8
42:8-PG	1.8	1.9	1.7	1.6	1.5	1.2	1.3	1.1	1.3	1.1	1.3	1.3	1.5	1.3	1.3	1.2
42:7-PG	0.9	0.8	0.7	0.6	0.6	0.6	0.6	0.5	0.4	0.5	0.5	0.6	0.5	0.4	0.4	0.5
42:6-PG	0.2	0.2	0.2	0.1	0.2	0.1	0.1	0.1	0.1	0.1	0.1	0.1	0.1	0.1	0.1	0.1
44:12-PG	8.0	8.2	7.2	7.3	9.4	8.7	5.0	4.9	10.1	8.8	7.2	6.8	8.9	7.9	8.4	8.0
44:11-PG	5.0	5.2	4.6	4.4	6.8	6.1	4.0	3.6	6.4	5.3	4.7	4.3	6.0	4.9	5.8	5.2
44:10-PG	1.9	1.9	1.7	1.5	2.8	2.2	1.6	1.4	2.4	1.9	1.7	1.5	2.2	1.8	2.0	1.9
44:9-PG	0.5	0.6	0.5	0.4	0.8	0.6	0.5	0.4	0.5	0.4	0.4	0.4	0.5	0.5	0.5	0.5
**Total**	100.0	100.0	100.0	100.0	100.0	100.0	100.0	100.0	100.0	100.0	100.0	100.0	100.0	100.0	100.0	100.0

**Phosphtidylinositol**	**siCtr ethanol**		**siCtr 4-OHT**		**siBP1 ethanol**		**siBP1 4-OHT**		**siBP2 ethanol**		**siBP2 4-OHT**		**siBP1+2 ethanol**		**siBP1+2 4-OHT**	
30:1-PI	0.1	0.0	0.1	0.2	0.0	0.1	0.1	0.1	0.0	0.0	0.1	0.1	0.0	0.0	0.0	0.0
30:0-PI	0.1	0.1	0.1	0.1	0.3	0.3	0.2	0.3	0.1	0.1	0.1	0.1	0.3	0.4	0.3	0.4
32:2-PI	0.5	0.5	0.8	0.9	0.2	0.2	0.3	0.4	0.2	0.2	0.5	0.5	0.0	0.0	0.0	0.1
32:1-PI	2.7	2.8	2.8	3.0	2.7	2.8	2.5	3.1	2.0	2.0	2.2	2.4	1.3	1.5	1.4	1.5
32:0-PI	1.7	1.8	1.4	1.5	5.1	5.0	3.5	4.1	2.7	2.4	1.8	1.7	3.9	4.8	4.1	4.2
34:3-PI	0.4	0.4	0.7	0.8	0.1	0.1	0.3	0.3	0.2	0.2	0.4	0.4	0.1	0.1	0.1	0.1
34:2-PI	7.1	7.7	8.7	9.1	3.6	3.8	5.5	5.7	3.9	4.5	6.9	7.0	1.7	2.0	2.1	2.3
34:1-PI	15.5	16.3	13.4	15.6	17.8	18.2	18.5	18.0	13.5	13.3	13.2	13.5	14.3	13.6	14.0	14.0
36:4-PI	1.3	1.2	1.0	1.0	1.4	1.1	0.8	0.8	1.4	1.4	1.3	1.2	0.9	1.0	0.9	0.9
36:3-PI	2.7	2.7	3.8	3.8	1.5	1.6	2.1	2.1	1.9	2.0	2.5	2.7	1.6	1.4	1.4	1.6
36:2-PI	11.9	12.1	12.1	12.5	7.6	7.6	10.3	10.3	9.4	9.9	11.7	10.8	7.5	7.6	8.0	7.8
36:1-PI	9.3	9.5	9.3	9.8	10.0	9.5	12.4	12.2	9.6	10.0	9.3	8.5	13.2	13.5	14.0	13.3
36:0-PI	1.2	1.1	1.1	1.3	1.5	1.4	1.7	1.6	1.3	1.3	1.1	0.9	2.4	2.4	2.5	2.4
38:6-PI	0.6	0.6	0.5	0.5	0.6	0.7	0.5	0.5	0.7	0.7	0.7	0.7	0.6	0.6	0.6	0.6
38:5-PI	5.1	5.0	3.3	3.0	4.4	4.6	3.0	2.9	7.6	7.2	6.2	6.0	4.4	4.2	4.1	4.2
38:4-PI	14.7	14.3	11.3	10.0	19.7	18.3	12.5	11.4	21.3	19.8	15.8	15.2	19.1	17.9	16.5	16.0
38:3-PI	12.0	12.1	14.3	13.0	9.6	9.8	11.0	11.1	9.4	9.6	9.9	10.9	9.4	9.3	9.5	9.6
38:2-PI	3.2	3.2	4.4	4.2	2.1	2.4	3.3	3.4	1.9	2.1	2.4	2.6	1.9	2.0	2.1	2.3
38:1-PI	0.4	0.4	0.5	0.5	0.3	0.4	0.6	0.6	0.3	0.3	0.3	0.3	0.3	0.4	0.4	0.5
40:7-PI	0.8	0.7	0.7	0.6	0.6	0.6	0.5	0.6	1.2	1.3	1.5	1.6	0.6	0.6	0.7	0.7
40:6-PI	2.7	2.3	2.6	2.3	2.8	3.1	2.6	2.6	3.8	3.9	3.9	4.1	4.6	4.7	5.0	5.1
40:5-PI	3.3	2.9	3.1	2.8	4.5	4.7	4.0	4.0	4.4	4.4	4.3	4.5	6.9	6.9	6.9	6.9
40:4-PI	2.0	1.9	2.6	2.3	2.8	2.7	2.8	2.7	2.4	2.2	2.6	2.9	3.8	3.8	3.9	4.0
40:3-PI	0.6	0.6	1.1	1.0	0.6	0.7	0.8	0.8	0.5	0.5	0.7	0.8	0.7	0.8	0.8	0.9
42:7-PI	0.1	0.0	0.1	0.0	0.0	0.1	0.0	0.0	0.1	0.1	0.1	0.2	0.0	0.0	0.0	0.1
42:6-PI	0.1	0.0	0.1	0.1	0.1	0.1	0.1	0.1	0.2	0.2	0.3	0.3	0.2	0.2	0.2	0.3
42:5-PI	0.1	0.0	0.1	0.1	0.1	0.1	0.1	0.1	0.1	0.1	0.1	0.2	0.2	0.2	0.2	0.2
42:4-PI	0.0	0.0	0.1	0.1	0.0	0.0	0.0	0.0	0.0	0.1	0.1	0.1	0.1	0.1	0.1	0.1
**Total**	100.0	100.0	100.0	100.0	100.0	100.0	100.0	100.0	100.0	100.0	100.0	100.0	100.0	100.0	100.0	100.0

**Phosphatidylserine**	**siCtr ethanol**		**siCtr 4-OHT**		**siBP1 ethanol**		**siBP1 4-OHT**		**siBP2 ethanol**		**siBP2 4-OHT**		**siBP1+2 ethanol**		**siBP1+2 4-OHT**	
32:1-PS	0.8	0.7	1.8	2.1	0.3	0.4	0.6	0.9	0.3	0.4	0.7	0.9	0.1	0.1	0.1	0.2
32:0-PS	0.6	0.5	1.0	1.3	0.1	0.2	0.2	0.2	0.6	0.5	0.8	0.9	0.2	0.1	0.2	0.2
34:2-PS	0.7	1.0	1.1	1.6	0.2	0.3	0.4	0.4	0.3	0.3	0.5	0.7	0.1	0.1	0.1	0.2
34:1-PS	8.7	8.0	9.2	9.9	6.1	6.2	7.2	7.4	6.1	5.8	6.7	7.8	4.0	3.8	3.8	4.1
34:0-PS	1.6	1.3	1.5	1.5	1.0	0.9	0.9	0.9	2.2	1.8	2.0	2.0	0.9	0.8	0.6	0.7
36:3-PS	0.3	0.2	0.3	0.3	0.1	0.1	0.1	0.1	0.1	0.1	0.2	0.2	0.1	0.1	0.1	0.1
36:2-PS	4.9	5.7	5.1	4.7	3.2	3.1	3.5	3.5	3.5	3.6	3.9	4.2	3.0	3.0	2.9	3.0
36:1-PS	25.8	24.6	17.3	16.3	28.8	25.2	24.5	23.2	25.7	24.1	22.3	21.7	28.0	26.3	24.7	24.1
36:0-PS	3.1	2.7	1.8	1.8	3.3	2.8	2.7	2.8	3.1	2.9	2.7	2.6	3.3	3.3	3.0	3.0
38:6-PS	0.3	0.3	0.0	0.0	0.1	0.0	0.0	0.2	0.0	0.0	0.0	0.0	0.0	0.0	0.0	0.0
38:5-PS	1.6	1.7	0.4	0.4	1.6	0.2	0.2	0.2	0.1	0.1	0.2	0.2	0.1	0.1	0.1	0.1
38:4-PS	2.2	2.2	1.6	1.5	1.1	1.6	1.6	1.8	1.1	1.1	1.3	1.2	1.1	1.4	1.4	1.4
38:3-PS	2.2	2.3	1.8	1.8	2.2	2.6	2.0	2.1	2.1	2.0	1.9	1.8	1.8	2.3	2.4	2.3
38:2-PS	0.7	0.6	0.5	0.5	0.3	0.4	0.3	0.4	0.5	0.5	0.5	0.5	0.4	0.5	0.4	0.5
38:1-PS	0.3	0.2	0.1	0.2	0.1	0.1	0.1	0.1	0.3	0.3	0.2	0.2	0.3	0.2	0.2	0.2
40:7-PS	0.1	0.2	0.3	0.2	0.1	0.2	0.2	0.1	0.2	0.1	0.2	0.3	0.0	0.1	0.1	0.1
40:6-PS	2.4	2.8	3.9	3.2	2.5	6.2	3.8	4.5	5.6	5.5	4.4	5.1	3.0	2.1	2.5	1.6
40:5-PS	3.2	3.7	4.3	3.9	4.9	7.1	5.0	5.7	6.2	5.7	5.2	4.9	5.8	5.8	5.0	4.8
40:4-PS	0.8	1.3	2.8	1.8	2.9	3.6	2.8	2.9	2.7	2.6	2.5	2.2	2.6	2.6	3.0	2.2
40:3-PS	0.3	0.3	0.5	0.4	0.4	0.7	0.3	0.4	0.4	0.4	0.4	0.4	0.4	0.4	0.3	0.5
42:8-PS	0.6	0.6	0.5	0.6	0.6	0.6	0.5	0.4	0.7	0.7	0.7	0.6	0.5	0.5	0.5	0.5
42:7-PS	1.4	1.4	2.4	2.7	0.8	0.8	1.4	1.3	0.9	1.1	1.4	1.5	0.8	0.8	1.0	0.9
42:6-PS	6.9	7.4	7.8	8.9	4.6	4.5	7.0	7.1	4.8	5.7	7.2	6.0	4.5	5.3	5.4	5.6
42:5-PS	4.6	5.1	5.4	6.1	5.8	5.1	7.4	7.3	4.5	5.1	4.8	4.1	7.6	8.1	8.7	8.4
42:4-PS	0.2	0.2	0.2	0.3	0.3	0.2	0.4	0.3	0.2	0.2	0.2	0.1	0.6	0.7	0.9	0.8
44:10-PS	0.3	0.3	0.3	0.3	0.3	0.3	0.3	0.2	0.3	0.4	0.4	0.4	0.3	0.3	0.3	0.3
44:9-PS	3.2	3.0	2.2	2.2	3.0	2.8	2.1	2.0	4.5	4.5	4.1	3.9	2.9	2.8	2.9	3.1
44:8-PS	9.0	8.8	7.9	7.5	12.8	11.2	8.6	7.6	11.9	11.8	9.9	9.8	12.1	11.8	11.4	11.8
44:7-PS	6.4	6.8	9.4	9.2	4.8	4.9	7.0	7.2	4.2	4.8	5.6	6.2	4.6	4.9	5.4	5.9
44:6-PS	1.3	1.3	2.1	2.1	0.9	0.9	1.5	1.6	0.6	0.8	0.9	1.0	0.8	0.9	0.9	1.1
44:5-PS	0.1	0.1	0.1	0.2	0.1	0.2	0.2	0.2	0.0	0.1	0.0	0.0	0.1	0.1	0.2	0.2
46:11-PS	0.4	0.3	0.4	0.4	0.3	0.4	0.3	0.4	0.6	0.7	0.9	0.9	0.3	0.4	0.4	0.5

46:10-PS	1.7	1.4	1.9	1.8	1.9	2.0	1.9	2.0	2.2	2.5	2.7	2.7	3.1	3.3	3.5	3.9
46:9-PS	2.0	1.8	2.1	2.0	2.8	2.8	2.8	2.7	2.4	2.5	2.8	2.8	4.3	4.5	4.8	4.9
46:8-PS	1.1	0.9	1.6	1.6	1.6	1.5	1.7	1.6	1.0	1.1	1.5	1.6	2.0	2.2	2.4	2.5
46:7-PS	0.3	0.2	0.5	0.6	0.2	0.2	0.3	0.4	0.1	0.1	0.3	0.4	0.2	0.2	0.3	0.3
**Total**	100.0	100.0	100.0	100.0	100.0	100.0	100.0	100.0	100.0	100.0	100.0	100.0	100.0	100.0	100.0	100.0

**Sphingosine**	**siCtr ethanol**		**siCtr 4-OHT**		**siBP1 ethanol**		**siBP1 4-OHT**		**siBP2 ethanol**		**siBP2 4-OHT**		**siBP1+2 ethanol**		**siBP1+2 4-OHT**	
C18-SG	3.55	3.02	3.19	3.22	0.83	1.28	1.08	1.51	4.35	4.65	3.00	3.85	0.91	0.91	1.61	1.98

**Sphingomyelin**	**siCtr ethanol**		**siCtr 4-OHT**		**siBP1 ethanol**		**siBP1 4-OHT**		**siBP2 ethanol**		**siBP2 4-OHT**		**siBP1+2 ethanol**		**siBP1+2 4-OHT**	
12:0-SM	0.2	0.2	0.2	0.2	0.1	0.1	0.1	0.1	0.1	0.1	0.1	0.2	0.0	0.0	0.0	0.0
14:1-SM	0.1	0.1	0.1	0.1	0.0	0.1	0.0	0.1	0.1	0.1	0.1	0.0	0.0	0.0	0.0	0.0
14:0-SM	9.2	9.5	10.2	10.7	8.9	9.5	8.3	9.6	10.0	9.1	9.1	9.2	6.3	6.5	6.1	6.2
16:1-SM	4.9	4.6	4.4	4.3	4.1	4.0	4.0	4.0	3.3	3.4	3.2	3.3	2.2	2.3	2.3	2.4
16:0-SM	49.7	50.0	47.9	47.0	51.8	52.0	51.4	53.7	48.3	48.5	47.3	46.1	50.0	48.5	49.3	48.7
18:1-SM	0.7	0.6	0.5	0.6	0.7	0.7	0.5	0.5	0.8	0.9	0.6	0.7	0.4	0.4	0.4	0.5
18:0-SM	2.2	2.2	1.8	1.7	2.6	2.7	2.2	2.1	4.4	4.2	3.4	3.3	3.7	3.7	3.4	3.5
20:1-SM	0.0	0.1	0.1	0.1	0.0	0.0	0.0	0.0	0.1	0.0	0.0	0.1	0.0	0.0	0.0	0.0
20:0-SM	0.5	0.5	0.4	0.5	0.6	0.5	0.4	0.4	0.8	0.7	0.7	0.7	0.6	0.6	0.6	0.6
22:1-SM	1.0	1.1	1.3	1.3	0.7	0.7	0.7	0.8	0.9	0.8	1.0	1.0	0.4	0.3	0.4	0.4
22:0-SM	4.0	4.1	4.4	4.5	5.2	5.4	5.0	5.3	5.9	5.4	5.4	5.3	5.7	5.5	5.6	5.7
24:3-SM	0.2	0.2	0.3	0.3	0.1	0.1	0.1	0.1	0.2	0.2	0.3	0.3	0.1	0.1	0.1	0.1
24:2-SM	2.6	2.6	2.8	2.9	1.6	1.5	1.6	1.4	1.9	1.9	2.5	2.8	1.1	1.1	1.0	1.1
24:1-SM	12.4	12.3	13.4	13.4	9.6	9.5	11.0	9.3	10.0	10.5	11.7	12.3	7.8	8.2	7.9	8.1
24:0-SM	9.5	9.3	9.4	9.3	12.7	11.9	13.3	11.8	10.4	10.7	10.6	10.4	18.4	19.1	19.6	19.1
26:4-SM	0.1	0.1	0.2	0.3	0.0	0.0	0.0	0.0	0.1	0.1	0.4	0.4	0.1	0.1	0.0	0.1
26:3-SM	0.2	0.2	0.3	0.2	0.0	0.1	0.0	0.0	0.3	0.3	0.4	0.5	0.2	0.2	0.2	0.2
26:2-SM	0.7	0.7	0.8	0.8	0.2	0.2	0.2	0.2	0.5	0.6	0.8	0.9	0.4	0.5	0.4	0.4
26:1-SM	1.3	1.3	1.3	1.2	0.6	0.7	0.7	0.5	1.3	1.5	1.5	1.7	1.2	1.3	1.2	1.3
26:0-SM	0.6	0.5	0.4	0.5	0.4	0.5	0.4	0.3	0.7	0.9	0.7	0.9	1.3	1.5	1.5	1.5
**Total**	100.0	100.0	100.0	100.0	100.0	100.0	100.0	100.0	100.0	100.0	100.0	100.0	100.0	100.0	100.0	100.0

**Triacylglycerol**	**siCtr ethanol**		**siCtr 4-OHT**		**siBP1 ethanol**		**siBP1 4-OHT**		**siBP2 ethanol**		**siBP2 4-OHT**		**siBP1+2 ethanol**		**siBP1+2 4-OHT**	
42:1-TG	0.0	0.0	0.0	0.0	0.0	0.0	0.0	0.0	0.0	0.0	0.0	0.0	0.0	0.0	0.0	0.0
42:0-TG	0.0	0.0	0.0	0.0	0.0	0.0	0.0	0.0	0.0	0.0	0.0	0.0	0.0	0.1	0.0	0.0
44:1-TG	0.2	0.2	0.3	0.4	0.2	0.2	0.3	0.3	0.1	0.1	0.2	0.3	0.1	0.1	0.1	0.2
44:0-TG	0.2	0.2	0.2	0.2	0.4	0.4	0.3	0.3	0.2	0.2	0.2	0.2	0.1	0.3	0.3	0.3
46:3-TG	0.2	0.2	0.8	0.9	0.0	0.1	0.2	0.2	0.0	0.0	0.2	0.3	0.0	0.0	0.1	0.0
46:2-TG	0.9	0.9	1.6	1.6	0.5	0.4	0.9	0.9	0.4	0.5	0.7	0.9	0.1	0.2	0.2	0.3
46:1-TG	1.5	1.5	1.4	1.5	1.6	1.8	1.6	1.9	1.2	1.3	1.4	1.4	0.8	1.0	1.0	1.0
48:4-TG	0.2	0.2	0.7	0.9	0.1	0.0	0.2	0.2	0.0	0.0	0.2	0.3	0.0	0.0	0.0	0.1
48:3-TG	1.6	1.4	2.8	3.1	0.5	0.5	1.2	1.3	0.4	0.6	1.1	1.4	0.2	0.4	0.4	0.6
48:2-TG	4.3	4.0	4.8	4.8	2.4	3.1	3.6	3.9	2.1	2.6	3.5	3.9	1.5	1.7	1.6	1.9
48:1-TG	5.0	5.1	3.9	3.8	6.7	7.5	5.7	6.1	5.2	5.3	5.1	4.9	4.7	4.7	4.1	4.4
48:0-TG	2.1	2.1	1.3	1.2	5.9	5.9	3.2	3.2	3.8	3.5	2.7	2.3	4.7	4.6	4.2	4.2
50:5-TG	0.2	0.2	0.5	0.6	0.1	0.0	0.2	0.2	0.1	0.2	0.3	0.3	0.0	0.1	0.1	0.2
50:4-TG	1.2	1.0	2.3	2.5	0.4	0.4	0.9	0.9	0.5	0.5	1.1	1.2	0.2	0.4	0.5	0.6
50:3-TG	5.1	4.8	6.5	6.8	1.9	2.2	3.5	4.0	1.8	2.3	3.7	4.5	1.3	1.7	1.8	2.1
50:2-TG	10.6	10.7	9.8	9.6	7.4	8.5	9.1	10.3	6.7	7.7	9.0	9.6	5.4	5.4	5.3	5.7
50:1-TG	9.0	9.4	6.8	6.7	15.4	15.6	12.3	12.1	12.3	12.2	10.0	9.1	12.5	11.8	10.7	11.1
50:0-TG	2.0	2.0	1.3	1.1	6.9	6.3	4.0	3.5	5.2	4.7	3.0	2.6	7.0	6.9	6.2	6.2
52:6-TG	0.3	0.2	0.4	0.5	0.1	0.2	0.2	0.2	0.3	0.4	0.5	0.5	0.2	1.2	0.4	0.4
52:5-TG	1.0	0.8	1.4	1.6	0.5	0.5	0.6	0.7	0.8	0.8	1.3	1.2	0.6	1.2	0.9	1.1
52:4-TG	2.5	2.4	3.7	4.0	1.1	1.1	1.8	1.8	1.6	1.6	2.3	2.6	1.4	1.7	1.7	1.8
52:3-TG	9.5	8.6	9.2	9.1	3.5	4.2	5.8	6.8	4.2	4.7	6.4	7.2	3.6	3.8	3.7	4.3
52:2-TG	12.3	12.5	10.5	10.1	9.9	10.7	11.7	11.9	9.9	10.8	10.8	10.8	8.7	8.0	7.8	7.8
52:1-TG	4.5	4.8	3.3	3.1	10.2	9.0	7.8	6.9	9.8	8.7	6.5	5.4	11.1	9.6	9.7	9.0
52:0-TG	0.8	0.8	0.4	0.4	3.8	2.9	2.3	1.7	3.4	2.7	1.6	1.4	5.1	4.3	4.7	4.4
54:7-TG	0.3	0.2	0.3	0.3	0.2	0.1	0.2	0.1	0.5	0.6	0.6	0.5	0.3	0.7	0.6	0.6
54:6-TG	0.9	0.8	0.8	1.0	0.6	0.6	0.6	0.6	1.4	1.4	1.5	1.3	1.3	1.4	1.5	1.6
54:5-TG	1.6	1.5	1.9	2.0	1.0	1.0	1.0	1.0	1.9	1.8	2.1	2.0	1.9	2.0	2.2	2.3
54:4-TG	3.1	2.6	3.2	3.2	1.4	1.4	1.7	1.9	2.1	2.2	2.6	2.9	2.6	2.5	2.6	2.7
54:3-TG	7.4	7.4	7.4	6.9	3.2	3.7	5.1	5.3	4.3	4.6	5.3	5.9	4.3	3.9	4.0	4.1
54:2-TG	3.6	4.1	3.1	3.0	4.3	3.9	4.6	4.2	4.6	4.4	4.0	3.8	4.5	4.1	4.1	3.9
54:1-TG	0.8	1.1	0.7	0.7	3.0	2.4	2.5	1.9	3.1	2.6	1.8	1.4	3.6	3.3	4.0	3.6
54:0-TG	0.1	0.1	0.1	0.1	0.8	0.5	0.5	0.3	0.6	0.5	0.4	0.2	1.0	0.9	1.3	1.1
56:8-TG	0.2	0.1	0.1	0.2	0.1	0.1	0.1	0.1	0.3	0.2	0.4	0.3	0.2	0.3	0.4	0.4
56:7-TG	0.6	0.6	0.4	0.4	0.4	0.4	0.3	0.3	1.2	1.1	1.1	0.9	1.2	1.3	1.4	1.2
56:6-TG	1.0	0.9	0.8	0.7	0.8	0.7	0.6	0.5	1.8	1.7	1.6	1.3	1.9	1.9	2.3	2.0
56:5-TG	1.2	1.1	1.3	1.3	0.9	0.7	0.7	0.7	1.7	1.5	1.3	1.5	2.3	1.9	2.1	2.0
56:4-TG	1.1	1.1	1.4	1.3	0.5	0.5	0.8	0.7	1.2	1.2	1.1	1.2	1.7	1.6	1.5	1.4
56:3-TG	1.0	1.2	1.3	1.2	0.6	0.5	0.8	0.8	0.9	0.8	0.9	1.0	0.9	1.0	0.9	0.9
56:2-TG	0.4	0.6	0.5	0.6	0.6	0.4	0.6	0.6	0.6	0.6	0.6	0.5	0.4	0.4	0.6	0.6
56:1-TG	0.1	0.2	0.1	0.1	0.5	0.3	0.4	0.3	0.4	0.4	0.3	0.2	0.2	0.3	0.6	0.4
56:0-TG	0.0	0.0	0.0	0.0	0.2	0.1	0.1	0.1	0.0	0.1	0.0	0.0	0.0	0.1	0.2	0.2
58:8-TG	0.1	0.2	0.1	0.1	0.1	0.0	0.1	0.1	0.4	0.4	0.4	0.3	0.3	0.4	0.4	0.4
58:7-TG	0.2	0.3	0.2	0.2	0.2	0.2	0.2	0.1	0.7	0.6	0.6	0.5	0.6	0.7	0.8	0.7
58:6-TG	0.3	0.3	0.4	0.3	0.2	0.2	0.2	0.1	0.8	0.7	0.6	0.6	0.9	0.9	1.1	0.9
58:5-TG	0.2	0.3	0.5	0.5	0.1	0.1	0.1	0.1	0.4	0.4	0.4	0.3	0.7	0.6	0.8	0.6
58:4-TG	0.1	0.2	0.4	0.4	0.0	0.0	0.1	0.1	0.2	0.1	0.2	0.2	0.2	0.2	0.4	0.2
58:3-TG	0.2	0.3	0.5	0.4	0.1	0.1	0.2	0.2	0.1	0.1	0.2	0.2	0.0	0.1	0.1	0.1
58:2-TG	0.2	0.4	0.3	0.4	0.3	0.2	0.5	0.3	0.2	0.2	0.2	0.3	0.0	0.1	0.1	0.1
58:1-TG	0.1	0.2	0.1	0.1	0.5	0.4	0.5	0.4	0.3	0.3	0.1	0.2	0.1	0.1	0.2	0.2
58:0-TG	0.0	0.0	0.0	0.0	0.2	0.1	0.2	0.1	0.1	0.1	0.0	0.1	0.0	0.0	0.1	0.1
**Total**	100.0	100.0	100.0	100.0	100.0	100.0	100.0	100.0	100.0	100.0	100.0	100.0	100.0	100.0	100.0	100.0
