# Supplementary material for: Sterol regulatory element binding protein-dependent regulation of lipid synthesis supports cell survival and tumor growth
Source: Cancer Metab. 2013 Jan 23;1:3. doi: 10.1186/2049-3002-1-3 (PMC3835903; doi:10.1186/2049-3002-1-3)
Supplement: Additional file 8 — Table S4. Saturation levels. [file 2049-3002-1-3-S8.pdf]

Table S4: Saturation levels

Diacylglycerol	percentages															
No of double bonds	siControl ethanol		siControl 4-OHT		siBP1 ethanol		siBP1 4-OHT		siBP2 ethanol		siBP2 4-OHT		siBP1+2 ethanol		siBP1+2 4-OHT	
0	13.2	12.7	9.9	10.1	29.3	26.7	19.2	18.0	23.9	20.6	16.7	14.5	30.5	31.3	31.4	32.8
1	38.0	40.5	35.6	37.4	39.2	41.9	41.3	41.4	37.9	40.2	39.9	38.1	36.9	37.5	37.8	37.1
2	33.3	33.3	39.2	39.1	16.5	17.7	25.8	27.6	17.9	20.2	27.6	29.8	13.8	14.0	15.2	15.7
3	7.9	7.5	9.8	8.9	6.2	5.9	6.9	7.0	6.8	6.6	6.9	7.9	6.4	6.6	5.5	5.2
4	4.2	3.3	3.3	2.6	5.4	4.7	3.8	3.3	8.0	7.0	4.8	5.2	6.3	5.2	5.1	4.5
5	2.1	1.8	1.5	1.3	2.3	2.1	2.0	1.7	3.6	3.4	2.7	2.9	3.8	3.4	3.3	3.1
6	1.0	0.8	0.6	0.5	1.0	0.8	0.9	0.9	1.7	1.7	1.1	1.4	2.0	1.8	1.5	1.5
7	0.2	0.2	0.1	0.1	0.1	0.1	0.1	0.1	0.2	0.3	0.3	0.2	0.3	0.2	0.2	0.2

free fatty acids	percentages															
No of double bonds	siControl ethanol		siControl 4-OHT		siBP1 ethanol		siBP1 4-OHT		siBP2 ethanol		siBP2 4-OHT		siBP1+2 ethanol		siBP1+2 4-OHT	
0	20.1	21.6	15.9	17.9	26.7	34	31	31	25.8	28.5	25.9	28.8	35.1	31.2	37.6	37
1	38.9	38.7	43.2	43.6	26.7	30.3	34.4	34.6	29.7	30.3	33	34.5	20.1	18.6	18.4	20.2
2	6.1	5.6	10.4	10.6	4.1	4.2	5.2	5.3	3.9	4.2	5	5	3.8	3.7	3.2	3.1
3	6.5	6	10.3	10	4.4	3.3	5.1	5.3	4	3.9	5.5	5.6	4.1	4.3	3.5	3.9
4	14.3	14.3	10.9	9.6	21.5	15.6	12.4	11.4	20.3	18	16.3	13.5	20.1	22.6	17.7	17.3
5	7	7.3	5.1	4.9	10	7.6	7.1	7.1	8.7	8.2	7.6	6.8	9.8	11.5	10.8	9.6
6	7	6.5	4.1	3.5	6.6	5	4.7	5.3	7.6	6.9	6.8	5.9	6.9	8.1	8.9	9

lysophosphocholine	percentages															
No of double bonds	siControl ethanol		siControl 4-OHT		siBP1 ethanol		siBP1 4-OHT		siBP2 ethanol		siBP2 4-OHT		siBP1+2 ethanol		siBP1+2 4-OHT	
0	53	54.8	48.4	45.1	62.6	62.3	56.8	59	63	57	57.2	55.4	69.3	62.7	69.7	67.6
1	40.7	40.3	44.9	47.2	31.7	33.1	38.7	36.6	32	37.5	38.2	39.7	24.9	28.1	24.7	26.4
2	2.3	2	3.1	3.3	1.9	1.8	1.9	2	1.6	1.9	1.7	1.8	1.8	4.1	1.9	1.9
3	1.3	1	1.3	1.6	1.1	0.8	0.9	0.9	0.9	0.9	0.6	0.9	1.1	1.4	1.2	1
4	1.8	1.3	1.8	2.1	1.6	1.2	1.1	1	1.6	1.6	1.7	1.6	1.7	2.5	1.6	1.8
5	0.4	0.3	0.3	0.3	0.5	0.4	0.3	0.4	0.5	0.5	0.3	0.4	0.6	0.8	0.6	0.7
6	0.5	0.3	0.2	0.4	0.4	0.3	0.2	0.2	0.5	0.6	0.2	0.4	0.6	0.4	0.4	0.5

phosphatidic acid	percentages															
No of double bonds	siControl ethanol		siControl 4-OHT		siBP1 ethanol		siBP1 4-OHT		siBP2 ethanol		siBP2 4-OHT		siBP1+2 ethanol		siBP1+2 4-OHT	
0	12.7	12.6	11.9	12.8	nd	33.8	18.8	18.6	32.8	31.5	22.4	16.3	50.1	58.5	49.5	56.8
1	43.8	44.5	43.9	42.6	nd	38.1	49.3	48.1	42.8	47.3	49.1	44.2	28.5	27.8	32.6	27.5
2	28.5	32.2	32.3	30.8	nd	16.1	18.1	19.1	11.9	12.9	18.4	21.5	6.9	6.6	8.7	6.9
3	3.9	3.3	5.9	6.6	nd	2.5	4.6	4.4	3.4	2.4	3.6	7.1	3.2	2.8	3.1	2.5
4	6	3.9	3.6	3.6	nd	4.5	4.4	4.6	4.3	2.7	2.1	4.8	3.9	2	2.6	2.6
5	3.2	2.1	1.8	1.9	nd	2.7	2.9	3.1	2.7	1.8	2.6	3.5	4.3	1.3	1.9	1.9
6	1.8	1.3	0.7	1.7	nd	2.3	1.9	2.1	2.2	1.5	1.8	2.7	3.2	1.1	1.7	1.8

phosphatidylcholine	percentages															
No of double bonds	siControl ethanol		siControl 4-OHT		siBP1 ethanol		siBP1 4-OHT		siBP2 ethanol		siBP2 4-OHT		siBP1+2 ethanol		siBP1+2 4-OHT	
0	30.3	29.3	16.3	19.1	34.6	28.6	18.9	25.3	24.7	22.6	19.4	28.1	30.9	21.7	22.1	24.1
1	37.5	36.3	40.1	45.3	37.7	36.8	40.0	44.7	41.8	38.5	31.8	36.4	33.1	28.9	27.6	31.1

2	13.3	13.7	23.9	19.8	9.5	10.5	20.0	13.6	13.7	16.7	18.0	15.2	9.5	10.7	11.1	12.6
3	3.7	4.0	6.2	4.5	3.2	3.4	5.7	3.4	3.8	5.2	6.7	4.1	4.2	6.0	6.1	6.4
4	4.6	5.1	4.6	3.9	5.0	6.0	5.5	4.1	5.1	5.9	8.9	4.8	6.8	10.8	11.2	9.6
5	4.7	5.2	4.2	3.4	4.8	6.0	4.9	4.1	4.9	5.1	7.9	5.0	6.6	9.8	9.9	8.0
6	3.4	3.7	2.7	2.3	3.4	5.1	3.2	2.9	3.6	3.8	4.8	3.7	5.4	7.7	7.7	5.5
7	1.9	2.1	1.5	1.3	1.5	2.6	1.5	1.5	1.9	1.8	1.9	2.0	2.4	2.9	2.9	1.9
8	0.3	0.3	0.3	0.2	0.3	0.5	0.3	0.3	0.3	0.3	0.3	0.4	0.6	0.6	0.6	0.4
9	0.2	0.2	0.1	0.1	0.2	0.4	0.2	0.2	0.3	0.2	0.3	0.2	0.5	0.9	0.9	0.5

**phosphatidylethanolam**	**percentages**															
**No of double bonds**	**siControl ethanol**		**siControl 4-OHT**		**siBP1 ethanol**		**siBP1 4-OHT**		**siBP2 ethanol**		**siBP2 4-OHT**		**siBP1+2 ethanol**		**siBP1+2 4-OHT**	
0	1.3	1.5	1.3	1.9	2.2	2.3	1.8	1.9	1.7	1.7	1.8	1.5	2.6	1.9	1.3	1.6
1	13.5	16.3	13	16.8	19.8	21.5	18.7	18.5	15.7	15.1	15.2	14.2	20.6	17.2	12	14.1
2	11.3	13.5	12.1	13.7	10.4	12.3	13.2	13.2	11.4	11.8	10.9	11.6	10.9	10.5	6.8	9.9
3	3.5	4.2	4.3	4.1	3.3	3.8	4.3	4.3	4.1	4.3	3.8	4.2	4	4.3	2.8	4.6
4	12.9	14.5	14.8	13.6	12.3	12.8	14.3	14.4	13	12.2	12.6	13.1	14.7	13.9	12.8	15.6
5	20.4	20.4	21.4	19.9	20.2	18.1	20.9	20.9	22	21.3	21.2	21.3	19.1	19.7	23.7	21.1
6	19.6	17.1	18.5	16.7	17.8	16.1	16.3	16.5	19.2	19.4	19.3	18.8	15.9	17.9	23.7	19
7	14.3	10.5	12	10.7	11.4	10.7	8.7	8.8	11	12.3	12.7	12.8	10.5	12.4	14.8	12.3
8	3	2	2.6	2.6	2.5	2.4	1.7	1.4	1.8	2	2.4	2.4	1.8	2.1	2.2	2
9	0	0	0	0	0	0	0	0	0	0	0	0	0	0	0	0

**phosphatidylglycerol**	**percentages**															
**No of double bonds**	**siControl ethanol**		**siControl 4-OHT**		**siBP1 ethanol**		**siBP1 4-OHT**		**siBP2 ethanol**		**siBP2 4-OHT**		**siBP1+2 ethanol**		**siBP1+2 4-OHT**	
0	1.1	1.2	1.5	1.7	2.8	3.1	2.6	2.8	1.9	1.8	1.4	1.4	3.3	3.8	3.3	3.6
1	9.6	9.9	11.8	15.5	16.2	18.4	17.4	18.0	13.6	13.2	12.3	12.6	16.6	17.1	15.6	15.2
2	15.0	14.9	17.8	20.6	12.0	14.3	18.5	20.3	12.6	16.0	17.7	18.4	10.1	12.0	10.4	10.9
3	5.3	5.8	7.6	8.5	4.4	4.8	6.3	6.8	4.7	5.4	6.9	7.3	4.7	5.4	4.8	5.0
4	4.3	4.3	5.8	5.7	3.9	3.7	4.6	4.8	3.6	4.0	5.0	5.1	4.8	5.2	5.0	5.3
5	6.0	5.9	6.2	5.3	5.9	5.7	6.6	6.2	6.0	6.0	6.5	6.6	6.5	6.6	6.9	7.1
6	11.2	10.9	8.9	7.4	11.1	10.8	10.7	10.1	11.0	11.3	10.3	10.1	11.9	11.7	12.3	12.2
7	21.1	20.1	15.7	12.6	14.1	13.6	14.1	13.4	16.7	16.9	16.0	15.9	12.9	12.6	12.8	13.6
8	4.8	4.8	4.5	3.7	3.6	3.0	3.3	3.0	3.9	3.8	3.8	3.9	4.6	4.2	4.4	4.2
9	3.4	3.5	3.5	2.8	3.4	2.9	2.7	2.3	3.1	2.8	3.0	2.9	3.6	3.2	3.7	3.6
10	4.6	4.7	4.5	4.1	5.6	4.5	3.8	3.3	5.6	4.3	4.6	4.2	5.4	4.7	5.5	5.3
11	5.7	5.9	5.1	4.9	7.5	6.6	4.5	4.0	7.2	5.9	5.3	4.8	6.7	5.6	6.6	6.0
12	8.0	8.2	7.2	7.3	9.4	8.7	5.0	4.9	10.1	8.8	7.2	6.8	8.9	7.9	8.4	8.0

**phosphatidylinositol**	**percentages**															
**No of double bonds**	**siControl ethanol**		**siControl 4-OHT**		**siBP1 ethanol**		**siBP1 4-OHT**		**siBP2 ethanol**		**siBP2 4-OHT**		**siBP1+2 ethanol**		**siBP1+2 4-OHT**	
0	2.9	3	2.6	2.9	6.9	6.7	5.4	6	4.1	3.8	3.1	2.7	6.5	7.5	7	6.9
1	28	29	26.1	29.1	30.9	31	34.1	34	25.3	25.7	25	24.8	29.2	29.1	29.9	29.3
2	22.7	23.4	25.9	26.7	13.5	14	19.5	19.9	15.4	16.8	21.5	20.9	11.1	11.7	12.2	12.5
3	15.7	15.7	19.9	18.6	11.9	12.2	14.1	14.3	12	12.4	13.5	14.8	11.8	11.6	11.8	12.2
4	18	17.5	14.9	13.3	23.9	22.2	16.2	15	25.1	23.6	19.8	19.4	23.9	22.8	21.3	21
5	8.5	7.9	6.5	5.9	9	9.4	7.1	7	12.1	11.6	10.6	10.7	11.4	11.3	11.2	11.3
6	3.4	2.9	3.2	2.9	3.5	3.9	3.1	3.3	4.7	4.8	4.8	5	5.4	5.5	5.9	6

7	0.8	0.7	0.8	0.6	0.6	0.7	0.5	0.6	1.3	1.4	1.6	1.7	0.6	0.6	0.7	0.7

phosphatidylserine	percentages															
No of double bonds	siControl ethanol		siControl 4-OHT		siBP1 ethanol		siBP1 4-OHT		siBP2 ethanol		siBP2 4-OHT		siBP1+2 ethanol		siBP1+2 4-OHT	
0	5.2	4.5	4.4	4.6	4.3	3.9	3.9	4	5.9	5.2	5.4	5.6	4.4	4.2	3.8	3.9
1	35.6	33.6	28.3	28.5	35.3	31.9	32.5	31.6	32.3	30.6	29.9	30.7	32.4	30.4	28.9	28.6
2	6.3	7.2	6.7	6.8	3.8	3.7	4.2	4.3	4.3	4.4	5	5.4	3.6	3.6	3.4	3.7
3	2.8	2.8	2.6	2.6	2.8	3.4	2.5	2.5	2.6	2.5	2.5	2.4	2.3	2.8	2.9	2.9
4	3.1	3.7	4.5	3.6	4.3	5.4	4.8	5	4	4	3.9	3.5	4.3	4.7	5.3	4.3
5	9.5	10.6	10.1	10.6	12.4	12.5	12.9	13.3	10.8	11	10.3	9.3	13.6	14.2	14	13.5
6	10.8	11.8	13.8	14.3	8	11.6	12.3	13.4	11.1	12	12.6	12.2	8.2	8.3	8.9	8.4
7	8.3	8.6	12.7	12.7	5.9	6.1	8.9	8.9	5.4	6.1	7.5	8.3	5.7	6	6.8	7.3
8	10.6	10.4	10.1	9.8	15	13.3	10.8	9.7	13.6	13.6	12.1	12	14.6	14.5	14.3	14.8
9	5.2	4.9	4.3	4.2	5.7	5.5	4.9	4.8	6.9	7	6.9	6.7	7.2	7.2	7.6	8
10	2	1.7	2.2	2	2.2	2.3	2.2	2.2	2.6	2.8	3.1	3.1	3.4	3.6	3.9	4.2
11	0.4	0.3	0.4	0.4	0.3	0.4	0.3	0.4	0.6	0.7	0.9	0.9	0.3	0.4	0.4	0.5

sphingomyelin	percentages															
No of double bonds	siControl ethanol		siControl 4-OHT		siBP1 ethanol		siBP1 4-OHT		siBP2 ethanol		siBP2 4-OHT		siBP1+2 ethanol		siBP1+2 4-OHT	
0	75.7	76.3	74.6	74.4	82.4	82.5	81	83.2	80.6	79.6	77.5	76	86.1	85.5	86.1	85.5
1	20.5	20	21	21	15.7	15.7	17	15.1	16.4	17.2	18.2	19.1	12.1	12.5	12.2	12.8
2	3.3	3.2	3.6	3.8	1.8	1.6	1.8	1.6	2.4	2.5	3.3	3.7	1.5	1.6	1.4	1.5
3	0.4	0.3	0.6	0.6	0.1	0.2	0.2	0.1	0.5	0.5	0.7	0.8	0.3	0.3	0.3	0.2
4	0.1	0.1	0.2	0.3	0	0	0	0	0.1	0.1	0.4	0.4	0.1	0.1	0	0.1

triacylglycerides	percentages															
No of double bonds	siControl ethanol		siControl 4-OHT		siBP1 ethanol		siBP1 4-OHT		siBP2 ethanol		siBP2 4-OHT		siBP1+2 ethanol		siBP1+2 4-OHT	
0	5.3	5.2	3.3	3.1	18.1	16.2	10.6	9.2	13.3	11.7	8	6.8	17.9	17.2	17.1	16.5
1	21.3	22.6	16.8	16.5	38.1	37.2	31.1	29.9	32.4	30.8	25.4	23	32.9	31	30.3	29.9
2	32.3	33.2	30.7	30.1	25.3	27.2	31	32.1	24.5	26.8	28.8	29.8	20.5	19.8	19.8	20.3
3	24.9	23.8	28.5	28.5	9.8	11.2	16.7	18.5	11.8	13.2	17.7	20.5	10.4	10.9	11	12.1
4	8.2	7.5	11.7	12.2	3.5	3.4	5.4	5.6	5.6	5.7	7.4	8.3	6.1	6.4	6.7	6.9
5	4.2	3.9	5.7	6.1	2.6	2.2	2.6	2.6	4.9	4.6	5.4	5.4	5.4	5.8	6.1	6.1
6	2.4	2.2	2.3	2.5	1.8	1.7	1.6	1.4	4.4	4.2	4.2	3.7	4.2	5.4	5.3	4.9
7	1.1	1.1	0.8	0.9	0.7	0.7	0.7	0.6	2.4	2.3	2.3	1.9	2	2.7	2.8	2.5
8	0.3	0.3	0.2	0.2	0.2	0.1	0.2	0.2	0.7	0.6	0.7	0.6	0.5	0.7	0.8	0.8
